# Chiral anion recognition using calix[4]arene-based ureido receptors in a *1,3-alternate* conformation

**DOI:** 10.3762/bjoc.16.249

**Published:** 2020-12-07

**Authors:** Tereza Horáčková, Jan Budka, Vaclav Eigner, Wen-Sheng Chung, Petra Cuřínová, Pavel Lhoták

**Affiliations:** 1 Department of Organic Chemistry, University of Chemistry and Technology, Prague (UCTP), Technicka 5, 166 28 Prague 6, Czech Republic; 2 Department of Solid State Chemistry, UCTP, 166 28 Prague 6, Czech Republic; 3 Department of Applied Chemistry, National Chiao Tung University, Hsinchu, 30050, Taiwan; 4 Institute of Chemical Process Fundamentals of Czech Academy of Sciences v.v.i., Rozvojová 135, 165 02 Prague 6, Czech Republic

**Keywords:** anion recognition, calixarene, chiral receptor, complexation, enantiodiscrimination

## Abstract

The introduction of chiral alkyl substituents into the lower rim of calix[4]arene immobilised in the *1,3-alternate* conformation led to a system possessing a preorganised ureido cavity hemmed with chiral alkyl units in the near proximity. As shown by the ^1^H NMR titration experiments, these compounds can be used as receptors for chiral anions in DMSO-*d*_6_. The chiral recognition ability can be further strengthened by the introduction of another chiral moiety directly onto the urea N atoms. The systems with double chiral units being located around the binding ureido cavity showed better stereodiscrimination, with the highest selectivity factor being 3.33 (*K*_L_/*K*_D_) achieved for *N*-acetyl-ʟ-phenylalaninate. The structures of some receptors were confirmed by single crystal X-ray analysis.

## Introduction

The recognition and complexation of anions has become undoubtedly one of the most important branches of modern supramolecular chemistry, as can easily be demonstrated by an immense number of recent reviews [1–6] and books [7–11] devoted to this topic. Due to the omnipresence of anions in biological systems, their irreplaceable roles in cell functioning have gradually been revealed and are well recognised to date. Consequently, given the importance of anions in many areas of everyday life, including, e.g., biology, medicine, environmental pollution issues, or industrial processes, the design and development of novel artificial receptors/sensors for anions is becoming more and more significant [12–14].

There are many strategies aiming at anion recognition in the literature. Most of the receptors, however, rely on electrostatic interactions. These systems are represented by positively charged molecules, such as quaternary N-, S-, and P-containing onium salts, protonated or alkylated aza-crown ethers and azacryptands, amidinium and guanidinium cations, etc. [15–18]. Due to the low directionality of the Coulomb force, the successful application of purely ionic interactions in the design of selective anion receptors is rather limited. The shapes and geometries of anions are widely different, and therefore the design of corresponding tailor-made receptors is based mostly on more directional interactions, such as hydrogen bonds. Indeed, an incredible number of neutral receptors bearing amide, sulfonamide, urea, thiourea, pyrrole, or triazole functional groups (to name at least some of them) has appeared during the last two decades [19–21].

Due to well-established functionalisation approaches, calix[4]arenes [22–24] are frequently used as molecular platform in the design of more complex receptor systems. The existence of four basic conformations (*cone*, *partial cone*, *1,3-alternate*, and *1,2-alternate*) offers the combination of a precisely defined 3D structure, with functional groups being introduced at exactly defined mutual positions. This makes calix[4]arenes an ideal molecular scaffold [25–26] for the construction of highly sophisticated molecules, including anionic receptors [27–39].

**Figure 1:**
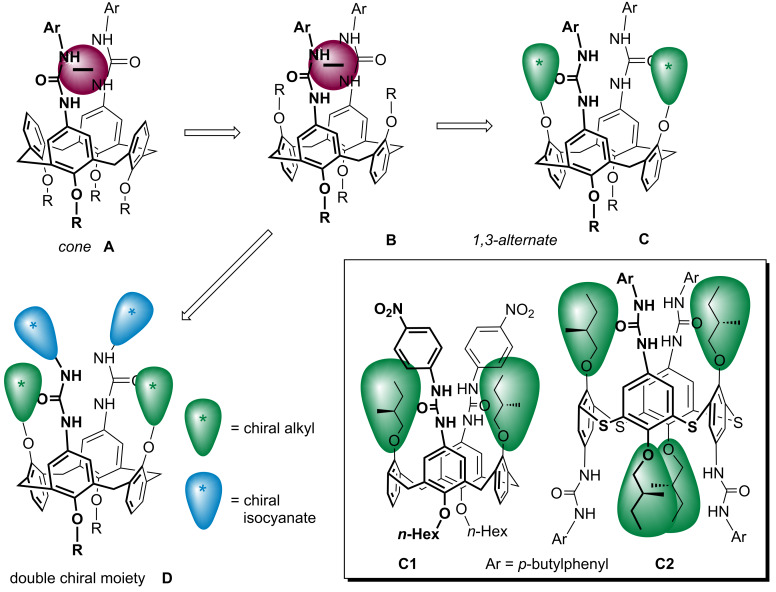
Design of chiral calix[4]arene-based receptors for anions.

During our ongoing research on anion complexation, we have reported various calix[4]arene receptors based mainly on amide, urea, or thiourea groups [40–41], some of which are available in different conformations. Although the overwhelming majority of calixarene-based receptors makes use of the *cone* conformer **A** (Figure 1), the corresponding diureidocalix[4]arenes in the *1,3-alternate* conformation **B** showed [42–43] surprisingly good complexation abilities towards selected anions. Especially for chiral anion recognition, contrary to the *cone* receptor, a design based on the *1,3-alternate* conformer enables the introduction of chiral units into the phenolic functions of the inverted aromatic moieties nearby the ureido cavity responsible for the binding, as in **C**. This design can be exemplified by our previously published receptors **C1** based on a calix[4]arene moiety or by **C2** using thiacalix[4]arene as the core scaffold [44–45]. Moreover, the introduction of the *tert*-butyl groups into the *1,3-alternate* conformer should lead to the overall increase rigidity of the molecule, possibly enhancing the interactions within the binding cavity. In this context, we realised that further strengthening of the chiral induction can be reached via the synchronous application of chiral units on the ureido moieties as well, as in **D**. In this paper, we report the preparation and complexation study of the latter type of receptor, bearing double chiral units in the immediate proximity to the preorganised ureido cavity.

## Results and Discussion

**Scheme 1:**
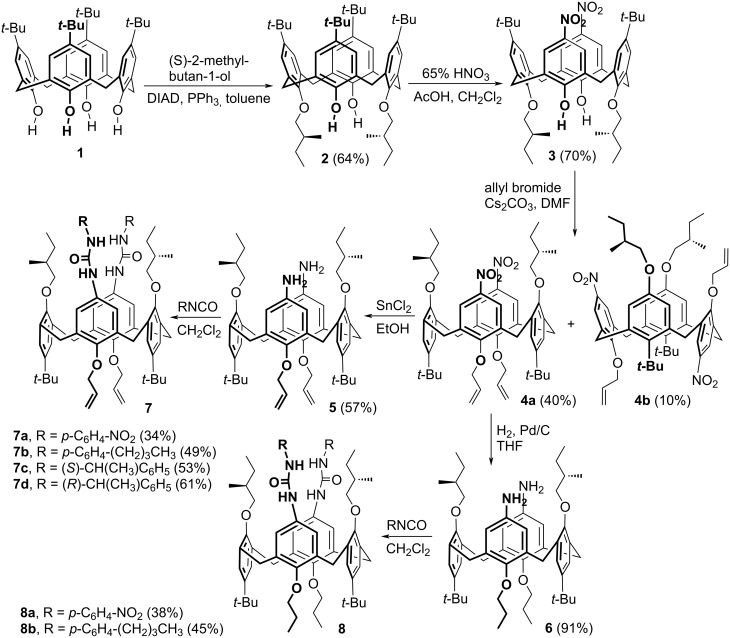
Synthesis of the calix[4]arene-based chiral anionic receptors **7** and **8**.

The introduction of the chiral alkyl moiety based on (*S*)-2-methylbutan-1-ol into the starting calix[4]arene **1** was carried out using recently described Mitsunobu reaction conditions [44]. Refluxing the reaction mixture of PPh_3_, DIAD, and toluene for two days provided the distally dialkylated calixarene **2** in 64% yield (Scheme 1). Compound **2** was regioselectively *ipso*-nitrated with 30 equiv of 65% aq HNO_3_ in an AcOH and CH_2_Cl_2_ mixture, making use of the higher reactivity of the nonalkylated phenolic moieties [45]. The fast reaction afforded the corresponding dinitro derivative **3** within a few minutes with 70% yield.

As we found in our previous attempts [43,45], the alkylation of dinitrocalixarenes to form the *1,3-alternate* conformation is a synthetic challenge. In fact, irrespective of the base or solvent used, the alkylation with appropriate *n*-propyl or *n*-hexyl halides always led to the *partial cone* conformation as the main product. To overcome this problematic step, we used the conditions described by Böhmer et al. [46] for a similar system bearing propyl groups on the lower rim. Indeed, one week of stirring **3** with allyl bromide in the presence of Cs_2_CO_3_ provided the expected *1,3-alternate* conformer **4a** in 40% yield accompanied by a small amount of the *partial cone* conformer **4b** (10%).

**Figure 2:**
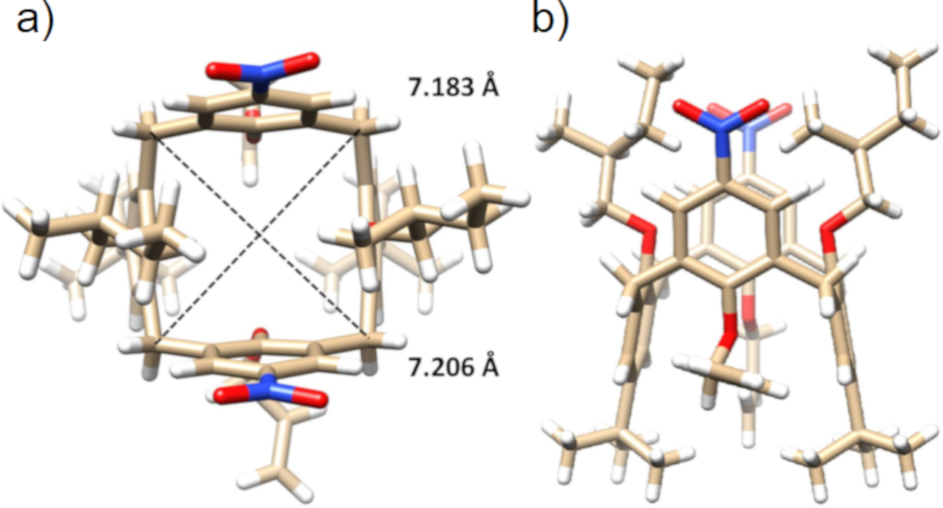
X-ray structure of **4a**: (a) Top view into the cavity. (b) Side view of the same cavity.

The unequivocal proof of the structure of the isomer **4a** was provided by single crystal X-ray analysis. The compound crystallised in a tetragonal system, space group *P*4_1_2_1_2 as a 1:1 complex with methanol used as a solvent for crystallisation. As shown in Figure 2, the calixarene clearly adopts the *1,3-alternate* conformation with an almost ideal tetragonal shape of the cavity. The lengths of the two main diagonals (the distances between opposite bridging CH_2_ moieties) are essentially identical (7.183 Å and 7.206 Å). If the main plane of the molecule is defined by the four bridging C atoms, all phenolic subunits are almost perpendicular to this plane, with the aromatic parts being slightly tilted out of the cavity. The corresponding interplanar angles Φ with the aromatic subunits are 81.55°, 80.78°, 77.08°, and 80.57°, respectively, starting counterclockwise from the upper subunit bearing a nitro group (Figure 2a).

The *1,3-alternate* isomer **4a** represents quite an interesting synthetic intermediate as the presence of the allyl groups immobilizes the required conformation and at the same time enables the potential incorporation of the macrocycle into a polymeric matrix. Consequently, the subsequent reduction step was carried out in two alternative ways: (i) exclusive reduction of the nitro groups or (ii) concomitant reduction of the nitro groups and the allyl moieties. Thus, the reaction of **4a** with SnCl_2_∙2H_2_O in ethanol gave the corresponding amine **5** in 57% yield after column chromatography on alumina. On the other hand, the four-day stirring of **4a** with Pd/C under a H_2_ atmosphere (5 atm) in an autoclave at room temperature provided compound **6** in 91% yield.

The starting compound **5** was then reacted with commercially available isocyanates comprising *p*-nitrophenyl isocyanate, *p*-*n*-butylphenyl isocyanate, (*S*)-α-methylbenzyl isocyanate, and (*R*)-α-methylbenzyl isocyanate. The reactions were carried out at room temperature in anhydrous dichloromethane, and the products **7a**–**d** were isolated in 40–60% yields. Similarly, the propyl-substituted analogues **8a** and **8b** were obtained from the reaction of **6** with the corresponding isocyanates in 38% and 45% yield, respectively.

The structures of final receptors **7a**–**d** and **8a**,**b** were confirmed by means of HRMS and NMR techniques. Thus, the HRESIMS (positive mode) analysis of **8** showed signals at *m*/*z* = 1141.59784 and 1157.57129, which is in a good agreement with the [M + Na]^+^ (1141.59846 Da) and [M + K]^+^ (1157.57240 Da) ions predicted for the product. The splitting pattern and multiplicity of the signals in the ^1^H NMR spectrum fully corresponded to the expected *1,3-alternate* conformation (see Supporting Information File 1). Thus, the two doublets with typical *ortho* substituent coupling constants (*J* = 7.3 Hz) at 7.64 and 8.14 ppm support the presence of *p*-nitrophenyl groups. At the same time, the singlets at 8.36 and 9.37 ppm reflected the ureido NH protons (DMSO-*d*_6_, 400 MHz, 298 K).

**Figure 3:**
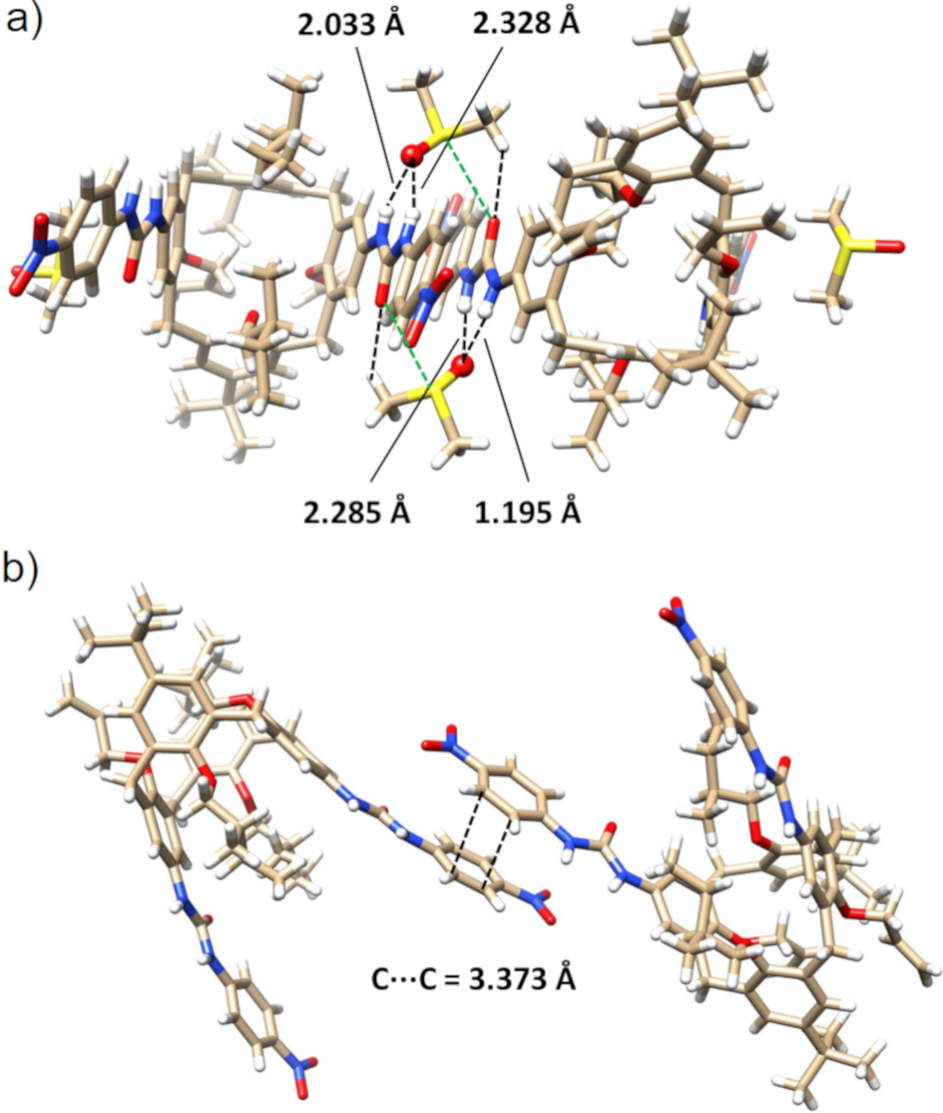
X-ray structure of **7a**: (a) Hydrogen bonding interactions (black) in a dimeric motif, chalcogen interactions are shown in green. (b) π–π interactions in the dimeric motif.

The structures of the selected receptors **7a** and **7d** were further proven by single crystal X-ray studies. The calixarene **7a** crystallised in a monoclinic system, space group *C*_2_, and the unit cell contained two receptors with four molecules of DMSO used as the crystallisation solvent (**7**·2DMSO complex). Both calixarene molecules exhibited an almost ideal square shape of the cavity, with the lengths of the main diagonals being 7.322 Å × 7.122 Å and 7.323 Å × 7.137 Å, respectively. Every ureido group held one molecule of DMSO via synchronous hydrogen bonding interactions between the two NH protons and a sulfoxide oxygen atom (Figure 3a). The S=O···H–N distances were 1.995, 2.285, 2.033, and 2.328 Å, indicating strong interactions in the solid state. At the same time, the carbonyl groups from the neighbouring receptor urea moieties interacted with the C–H bonds of the DMSO methyl group (the C=O···H–C distances were 2.486 and 2.452 Å), thus forming the calixarene dimer with a head-to-tail mutual orientation. The overall supramolecular binding motif was completed by the close contacts between carbonyl oxygen atoms (of the urea group) and S atoms (of DMSO), indicating possible chalcogen interactions [47–48], and the C=O···S=O distances were 3.269 and 3.308 Å (Figure 3a). The molecular packing was further strengthened by the π–π interactions of the *p*-nitrophenyl moieties, exhibiting several close C_Ar_···C_Ar_ contacts at a 3.373 Å distance (Figure 3b).

**Figure 4:**
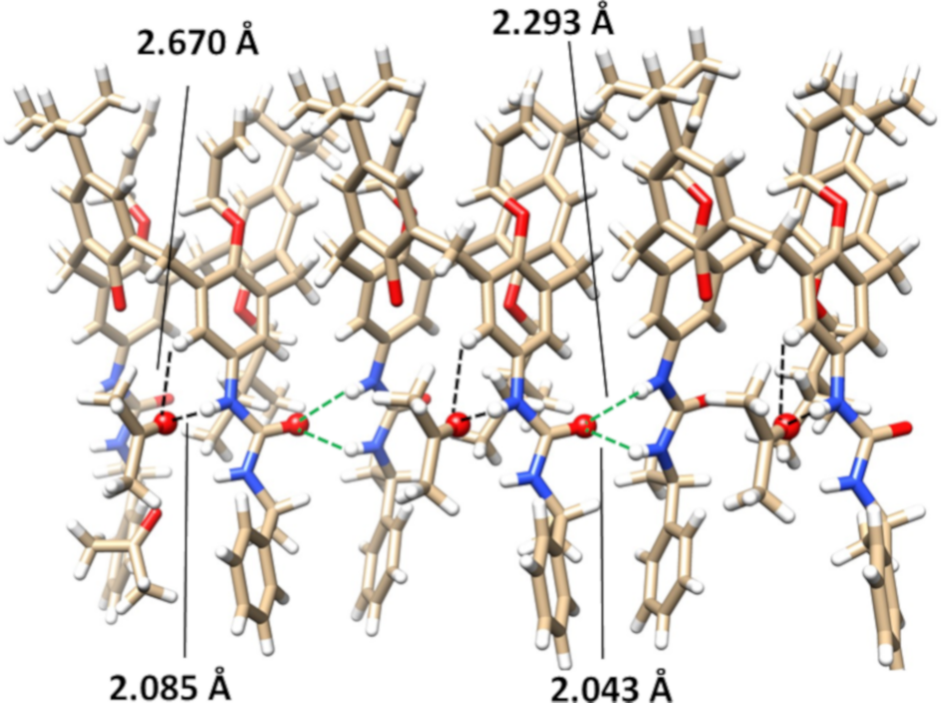
X-ray structure of **7d**, showing hydrogen bonds between the ureido units (green) and hydrogen bonding of acetone molecules (black). One alkyl group in each calixarene was removed for better clarity.

The receptor **7d** crystallised in a triclinic system, space group *P*_1_, as a 1:3 complex with acetone (used as solvent for crystallisation). The main packing motive (see Figure 4) was represented by an infinite chain of calixarene molecules joined together by intermolecular hydrogen bonds between the ureido groups (the C=O···H–N distances were 2.293 and 2.048 Å).

One of the ureido functions in the macrocycle also held acetone via a C=O···H–N hydrogen bond (2.085 Å) and via a C=O···H–C bond (2.670 Å) from the *meta*-position of the adjacent aromatic moiety (Figure 4).

The complexation ability of the novel receptors towards selected chiral anions was studied using standard ^1^H NMR titration experiments. There are several reasons why DMSO-*d*_6_ was selected as the solvent for the complexation studies: (i) it dissolves all anionic species tested, (ii) prevents the receptor molecule from self-association, and (iii) reduces the complexation constants to values easily measurable by ^1^H NMR titrations.

**Figure 5:**
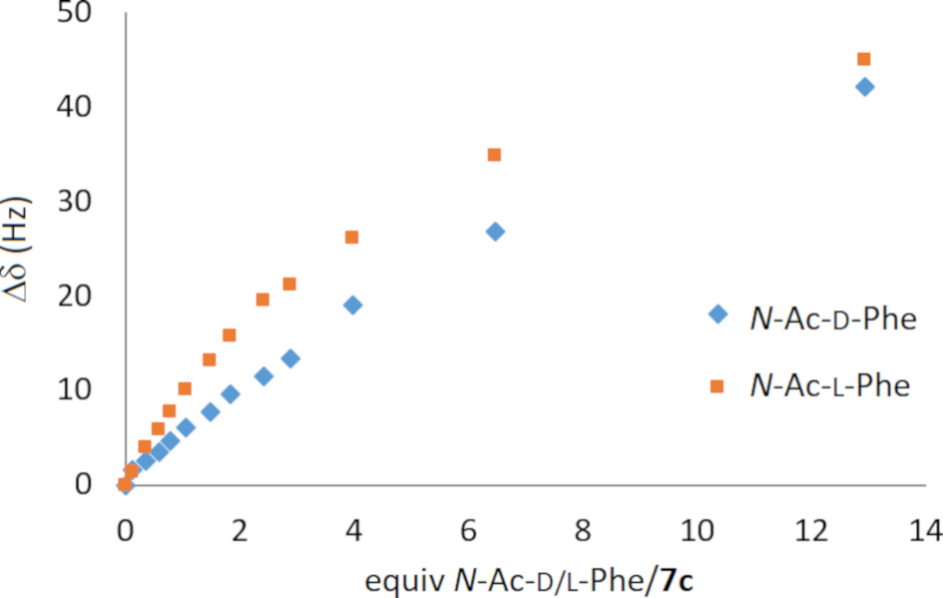
^1^H NMR titration of **7c** with *N*-acetyl-ᴅ-phenylalaninate and *N*-acetyl-ʟ-phenylalaninate (as TBA salts, 400 MHz, 298 K, DMSO-*d*_6_, the aromatic signal of the calixarene moiety was used).

A solution of an anion was gradually added to a solution of **7** or **8** in DMSO-*d*_6_ to obtain various calixarene/anion ratios of 1:0.3–1:15. Upon the addition of anions in the form of TBA salts, significant downfield shifts of the ureido NH signals were observed, indicating the complexation under fast exchange conditions [49]. The corresponding complexation constants were calculated based on the analysis of the binding isotherms obtained from the complexation-induced chemical shift (CIS) values of urea NH protons or aromatic CH signals (Figure 5) [50–52]. The nonlinear curve-fitting of the experimental data was performed using the freely available software Bindfit [53]. The stoichiometry of the complexes was determined based on the Bindfit output, where the 1:1 model provided the best fit among all tested stoichiometries (1:1, 1:2, and 2:1).

The results summarised in Table 1 and Table 2 revealed that the complexation properties of the receptors do not depend significantly on the lower-rim substitution of the calixarene. Comparing the complexation constants for the otherwise identical receptors (**7a** vs **8a** or **7b** vs **8b**), it is obvious that the presence of allyl vs propyl substituents does not impose much difference in terms of absolute *K* values and selectivity.

**Table 1:** Binding constants of the receptors **7a** and **8a** towards selected anions (^1^H NMR titration, 400 MHz, DMSO-*d*_6_, 298 K).

run^a^	anion	*K* (M^−1^) of **7a**	*s*^b^	*K* (M^−1^) of **8a**	*s*^b^

1	*N*-acetyl-ᴅ-phenylalaninate	279	1.18 (for ʟ)	290	1.10 (for ʟ)
2	*N*-acetyl-ʟ-phenylalaninate	330		320	
3	*N*-acetyl-ᴅ-leucinate	140	1.33 (for ʟ)	150	1.46 (for ʟ)
4	*N*-acetyl-ʟ-leucinate	190		220	
5	ᴅ-phenylalaninate	660	1.06 (for ʟ)	580	1.05 (for ʟ)
6	ʟ-phenylalaninate	700		610	
7	ᴅ-leucinate	480	1.02 (for ʟ)	320	1.12 (for ʟ)
8	ʟ-leucinate	490		360	
9	(*R*)-mandelate	260	1.03 (for ʟ)	250	1.04 (for ʟ)
10	(*S*)-mandelate	270		260	

^a^Runs 1–10: tetrabutylammonium (TBA) salts. ^b^Selectivity factor: *s* = *K*_D_/*K*_L_ or *K*_L_/K_D_ to obtain *s* ≥ 1.

**Table 2:** Binding constants of the receptors **7b** and **8b** towards selected anions (^1^H NMR titration, 400 MHz, DMSO-*d*_6_, 298 K).

run^a^	anion	*K* (M^−1^) of **7b**	*s*^b^	*K* (M^−1^) of **8b**	*s*^b^

11	*N*-acetyl-ᴅ-phenylalaninate	180	1.11 (for ʟ)	200	1.10 (for ʟ)
12	*N*-acetyl-ʟ-phenylalaninate	200		220	
13	*N*-acetyl-ᴅ-leucinate	20	2.00 (for ʟ)	16	1.87 (for ʟ)
14	*N*-acetyl-ʟ-leucinate	40		30	
15	ᴅ-phenylalaninate	90	1.11 (for ʟ)	105	1.04 (for ʟ)
16	ʟ-phenylalaninate	100		110	
17	ᴅ-leucinate	90	1.20 (for ʟ)	95	1.10 (for ʟ)
18	ʟ-leucinate	108		105	
19	(*R*)-mandelate	160	1.06 (for ʟ)	140	1.14 (for ʟ)
20	(*S*)-mandelate	170		160	

^a^Runs 11–20: TBA salts. ^b^Selectivity factor: *s* = *K*_D_/*K*_L_ or *K*_L_/*K*_D_ to obtain *s* ≥ 1.

As expected, the nitro-substituted receptors **7a** and **8a** exhibited complexation constants higher than the butyl-substituted analogues **7b** and **8b** for all anions – compare **7a** (Table 1, run 5, *K* = 660 M^−1^) vs **7b** (Table 2, run 15, *K* = 90 M^−1^). On the other hand, despite the differences in the *K* values, the enantioselectivity remained almost the same in both receptor series. Thus, the selectivity factor *s* = *K*_L_/*K*_D_ for phenylalaninate was 1.06 for **7a** and 1.11 for **7b**, and similar values were also obtained for **8a** (1.05) and **8b** (1.04), using the ʟ-enantiomer in each case. The highest chiral discrimination (for an ʟ-enantiomer) was achieved for *N*-acetyl-ʟ-leucinate, with a selectivity factor of *s* = 2.0 and 1.87 for **7b** and **8b**, respectively.

The above-mentioned results indicate that the binding cavity composed of two preorganised ureido groups and chiral alkyl substituents in the near proximity possesses some ability of enantioselective recognition. In fact, this assumption was already established by our previous receptors **C1** and **C2** (Figure 1) [44–45], although the direct comparison with our novel results is rather difficult due to the different types of derivatives (**C1** has a cavity without *tert*-butyl groups, and **C2** is based on thiacalix[4]arene). Nevertheless, the simultaneous introduction of chiral isocyanate to form chiral urea moieties has not been accomplished yet.

The formation of another chiral centre within the derivatives **7c** and **7d** led to the expected decrease [42] of the complexation constant values (by approximately one order of magnitude) due to the presence of alkyl instead of aryl urea receptors (Table 3). Thus, going from **7a** to **7c**, the complexation constant *K* for ᴅ-leucinate decreased from 480 to 50 (run 7, Table 1 vs run 27, Table 3), and the appropriate values for ᴅ-phenylalaninate are 660 vs 40 (run 5, Table 1 vs run 25, Table 3).

**Table 3:** Binding constants of the receptors **7c** and **7d** towards selected anions (^1^H NMR titration, 400 MHz, DMSO-*d*_6_, 298 K).

run^a^	anion	*K* (M^−1^) of **7c**	*s*^b^	*K* (M^−1^) of **7d**	*s*^b)^

21	*N*-acetyl-ᴅ-phenylalaninate	15	3.33 (for ʟ)	35	1.75 (for ᴅ)
22	*N*-acetyl-ʟ-phenylalaninate	50		20	
23	*N*-acetyl-ᴅ-leucinate	20	2.50 (for ʟ)	31	1.34 (for ᴅ)
24	*N*-acetyl-ʟ-leucinate	50		23	
25	ᴅ-phenylalaninate	40	1.37 (for ʟ)	25	1.28 (for ʟ)
26	ʟ-phenylalaninate	55		32	
27	ᴅ-leucinate	50	1.20 (for ʟ)	27	1.11 (for ʟ)
28	ʟ-leucinate	60		30	
29	(*R*)-mandelate	20	1.75 (for ʟ)	11	1.37 (for ʟ)
30	(*S*)-mandelate	35		8	

^a^Runs 21–30: TBA salts. ^b^Selectivity factor: *s* = *K*_D_/*K*_L_ or *K*_L_/*K*_D_ to obtain *s* ≥ 1.

On the other hand, the chiral recognition properties of the receptors **7c** and **7d** are accentuated compared to **7a** and **8a** or **7b** and **8b**. The introduction of another chiral centre leads to higher selectivity factors in almost all the cases. The stereodiscrimination of **7c** (bearing an (*S*)*-*α-methylbenzyl moiety on the urea group) for *N*-acetylphenylalaninates represents the maximum value achieved (*s* = 3.33 for ʟ). Interestingly, the diastereomeric isomer **7d**, possessing an (*R*)-chiral centre, prefers the ᴅ-isomer, with a selectivity factor of *s* = 1.75, and the same holds for *N*-acetyl-ᴅ-leucinate (*s* = 1.34). These results indicate that both chiral moieties (the alkyl group on the calixarene and the chiral centre on the urea moiety) function synergistically, and a proper choice of both substituents can lead to an even better stereoselectivity.

## Conclusion

In conclusion, the introduction of chiral alkyl groups into the lower rim of calix[4]arene immobilised in the *1,3-alternate* conformation resulted in a macrocycle with a preorganised ureido cavity bearing chiral alkyl substituents in the near proximity. As shown by ^1^H NMR titration experiments, these compounds function as receptors for chiral anions in DMSO-*d*_6_. The chiral recognition ability can further be strengthened by the introduction of another chiral moiety directly to the urea nitrogen atoms. The systems with double chiral units located around the binding ureido cavity showed a better stereodiscrimination, with the highest selectivity factor being 3.33 (for ʟ) achieved for *N*-acetylphenylalaninate.

## Supporting Information

File 1 Experimental details and characterisation data (including X-ray data for **4a**, **7a**, and **7d**, NMR, IR, and HRMS) as well as NMR titration data.
