# Supplementary material for: Chiral anion recognition using calix[4]arene-based ureido receptors in a 1,3-alternate conformation
Source: Beilstein J Org Chem. 2020 Dec 7;16:2999–3007. doi: 10.3762/bjoc.16.249 (PMC7736684; doi:10.3762/bjoc.16.249)

BEILSTEIN JOURNAL OF ORGANIC CHEMISTRY

Supporting Information

for

Chiral anion recognition using calix[4]arene-based ureido receptors in a *1,3-alternate* conformation

Tereza Horáčková, Jan Budka, Vaclav Eigner, Wen-Sheng Chung, Petra Cuřínová and Pavel Lhoták

Beilstein J. Org. Chem. **2020,** *16,* 2999–3007. doi:10.3762/bjoc.16.249

Experimental details and characterisation data (including X-ray data for 4a, 7a, and 7d, NMR, IR, and HRMS) as well as NMR titration data

License and Terms: This is a supporting information file under the terms of the Creative Commons Attribution License (https://creativecommons.org/licenses/by/4.0). Please note that the reuse, redistribution and reproduction in particular requires that the author(s) and source are credited and that individual graphics may be subject to special legal provisions.
The license is subject to the *Beilstein Journal of Organic Chemistry* terms and conditions: (https://www.beilstein-journals.org/bjoc/terms)

Table of contents

1. Experimental S2
2. X-ray measurements S15
3. Spectral characterisation of compounds S18
4. NMR titration data S70

Experimental

General experimental procedures

All chemicals were supplied by commercial sources and used without further purification. For TLC analysis, silica gel 60 F_{254} (Merck) and aluminium oxide 60 F_{254} neutral (Merck) foil sheets were used. Column chromatography was performed using silica gel Geduram 60 F_{254} (Merck) or aluminium oxide 90 standardised (Merck). The ^{1}H (400.1 MHz) and ^{13}C (100.6 MHz) NMR spectra were measured on an Agilent 400-MR DDR2 spectrometer and an Avance 400 spectrometer (Bruker, Germany) at 25 °C. The ^{1}H and ^{13}C NMR spectra were referenced to the line of the solvent (δ/ppm; δ_H/δ_C: $CDCl_3$ 7.26/77.16; DMSO-d_6 2.50/39.52). HRMS analyses were performed using Q-TOF (Micromass), using ESI ionisation in positive mode. Melting points were measured on a Heiztisch Mikroskop–Polytherm A (Wagner & Munz, Germany) and are not corrected. Polarimetry was performed on a Jasco P-2000 polarimeter (Jasco, Germany) with a Na lamp (589 nm, continuous) in the cuvette of 1 dm length at 20 °C, the measured values α are given in $deg \cdot mL \cdot dm^{-1} \cdot g^{-1}$. The concentration of samples measured was 0.01 g in 1 mL of solvent. Diffraction data were collected on a Bruker D8 VENTURE Kappa Duo PHOTON 100 CMOS with the monochromatic Mo/Cu-Kα radiation. The crystallographic data for the structures reported in this paper have been deposited with the Cambridge Crystallographic Data Centre as a supplementary publication. These data are provided free of charge by the joint Cambridge Crystallographic Data Centre and Fachinformationszentrum Karlsruhe Access Structures service www.ccdc.cam.ac.uk/structures. The FTIR analysis were performed on a FTIR spectrometer Nicolet iS10 Thermo Scientific in KBr transmission mode and on a Nicolet 6700 spectrometer (Thermo-Nicolet, U.S.A.) connected with a GladiATR diamond ATR adapter (PIKE, U.S.A.),

reflectance measurement, DTGS KBr detector, with the following parameters: spectral range: 4000–400 cm^{-1}, resolution: 4 cm^{-1}, number of spectra accumulations: 64, apodisation: Happ–Genzel. The spectra were processed by Omnic 9 (Thermo-Nicolet Instruments Co., U.S.A.) with baseline correction.

Synthetic procedures

5,11,17,23-Tetra-*tert*-butyl-26,28-bis[(*S*)-2-methylbutoxy]calix[4]arene-25,27-diol (**2**)

Starting 4-*tert*-butylcalix[4]arene (**1**, 5 g, 7.7 mmol) was dissolved in anhydrous toluene (300 mL), and then triphenylphosphine (12.1 g, 46.2 mmol) and (*S*)-2-methylbutan-1-ol (8.3 mL, 77.1 mmol) were added. The mixture was cooled to 0 °C and diisopropyl azodicarboxylate (9.2 mL, 46.7 mmol) was added dropwise. The solution was stirred at 0 °C for 5 minutes and then heated to 120 °C for 48 hours. After cooling to room temperature, the solvent was removed under reduced pressure and the liquid residue was precipitated from hot methanol (40 °C, 100 mL). The product was isolated by filtration and thoroughly washed with cold methanol to give 3.86 g of **2** (64%) in the form of a white solid (mp: 278 - 281 °C). ^{1}H NMR ($CDCl_3$, 400 MHz, 298 K) δ (ppm): 7.69 (s, 2H, -OH); 7.06 – 7.04 (m, 4H, Ar-H); 6.81 – 6.80 (m, 4H, Ar-H); 4.33 – 4.26 (m, 4H, Ar-CH_2-Ar); 3.85 – 3.75 (m, 4H, -O-CH_2-); 3.31 – 3.28 (m, 4H, Ar-CH_2-Ar); 2.09 – 2.01 (m, 2H,-CH-); 1.84 – 1.76 (m, 2H, -C*H*H-CH_3); 1.50 – 1.44 (m, 2H, -CH*H*-CH_3); 1.29 (s, 18H, *t*-Bu); 1.27 (d, 6H , *J* = 6.7 Hz, -CH_3); 1.04 – 1.00 (t, 6H, *J* = 7.4 Hz, -CH_3); 0.96 (s, 18H, *t*-Bu). ^{13}C NMR ($CDCl_3$, 101 MHz, 298 K) δ (ppm): 151.2; 149.9 (2x 2C, Ar-C, C-O-); 146.8; 141.3 (2x 2C, Ar-C, C-*t*-Bu); 132.9; 132.8; 127.8; 127.6 (4x 2C, Ar-C, *C*-CH_2-); 125.6; 125.5; 125.1; 125.0 (4x 2C, Ar-CH); 81.6 (2C, -O-CH_2-); 36.2 (2C, -CH-); 34.0; 33.9 (2x 2C, Ar-CH_2 -Ar); 31.8 (6C, -C(CH_3)$_3$); 31.6 (4C, -*C*(CH_3)$_3$); 31.1 (6C, -C(*C*H_3)$_3$); 26.3 (2C, -CH_2-); 16.9; 11.8 (2x 2C, -CH_3). HRMS-ESI:

($C_{54}H_{76}O_4$) m/z calcd: 811.5636 $[M+Na]^+$, found: 811.5636 $[M+Na]^+$. IR (KBr) ν (cm^{-1}): 3388, 2959, 2917, 2872, 1958, 1639, 1595, 1485, 1122. Optical rotation: $[\alpha]^{20}_{589}$ = 5.8° (c = 0.21 g/mL, $CHCl_3$).

5,17-Di-*tert*-butyl-26,28-bis[(*S*)-2-methylbutoxy]-11,23-dinitrocalix[4]arene-25,27-diol (**3**)

A mixture of glacial acetic acid (16.7 mL) and HNO_3 65% (7.4 mL, 172.8 mmol) was added to the vigorously stirred solution of the calix[4]arene **2** (4.4 g, 5.6 mmol) in dichloromethane (140 mL) at room temperature. Once the colour of the solution changed from dark purple to yellow (taking a few seconds to a few minutes), the reaction mixture was poured into water (300 mL). The organic phase was separated and the aqueous layer was extracted with dichloromethane (2×25 mL). The combined organic extracts were washed with water, dried over $MgSO_4$, and the solvent was then evaporated under reduced pressure. The product was isolated after crystallisation from CH_2Cl_2/MeOH to give 3 g (70%) of compound **3** in the form of a yellow powder (mp: 277 – 279 °C). ^{1}H NMR ($CDCl_3$, 400 MHz, 298 K) δ (ppm): 9.17 (s, 2H, -OH); 8.08 – 8.05 (m, 4H, Ar-H); 7.01 – 6.75 (m, 4H, Ar-H); 4.32 – 4.23 (m, 4H, Ar-CH_2-Ar); 3.89 – 3.78 (m, 4H, -O-CH_2-); 3.50 – 3.44 (m, 4H, Ar-CH_2-Ar); 2.15 – 2.04 (m, 2H, -CH-); 1.82 – 1.72 (m, 2H, -C*H*H-CH_3); 1.53 – 1.44 (m, 2H, -CH*H*-CH_3); 1.29 (d, 6H, *J* = 6.8 Hz, -CH_3); 1.05 (t, 6H, *J* = 7.5 Hz, -CH_3); 1.01 (s, 18H, *t*-Bu). ^{13}C NMR ($CDCl_3$, 101 MHz, 298 K) δ (ppm): 159.9; 149.7 (2x 2C, Ar-C, C-O-); 148.6 (2C, Ar-C, C-*t*-Bu); 139.8 (2C, Ar-C, C-NO_2); 131.1; 130.9; 128.9; 128.6 (4x 2C, Ar-C, *C*-CH_2-); 126.4; 126.3 (2x 2C, Ar-CH); 124.5 (4C, Ar-CH); 82.4 (2C, -O-CH_2-); 36.0 (2C, -CH -); 34.3 (2C, -*C*(CH_3)$_3$); 31.6 (2C, Ar-CH_2 -Ar); 31.3 (6C, -C(CH_3)$_3$); 31.2 (2C, Ar-CH_2 -Ar); 26.3 (2C, -CH_2 -); 16.9; 11.7 (2x 2C, -CH_3). HRMS-ESI: ($C_{46}H_{58}N_2O_8$) m/z calcd: 789.4087 $[M+Na]^+$, 805.3826

$[M+K]^+$, found: 789.4081 $[M+Na]^+$, 805.3820 $[M+K]^+$. IR (KBr) ν (cm^{-1}): 3365, 2958, 2917, 2849, 1645, 1593, 1514, 1109. Optical rotation: $[\alpha]_{589}^{20}$ = 12.6° (c = 1 g/mL, $CHCl_3$).

1,3-Alternate 26,28-diallyloxy-11,23-di-*tert*-butyl-25,27-bis[(*S*)-2-methylbutoxy]-5,17-dinitrocalix[4]arene (**4a**)

The calix[4]arene **3** (3 g, 3.9 mmol) and Cs_2CO_3 (19.1 g, 58.7 mmol) were dissolved in dry DMF (75 mL) and heated to 40 °C. After 1 hour, allyl bromide (5.1 mL, 58.7 mmol) was added, and the reaction mixture was stirred under an argon atmosphere at 40 °C for 7 days. The solvent was then evaporated under reduced pressure and the crude product was dissolved in a mixture of $CHCl_3$ (38 mL) and water (112 mL). The organic phase was washed with water (2×80 mL), dried over $MgSO_4$, and the solvent was removed on a vacuum evaporator. The crude product was purified by column chromatography on silica gel using cyclohexane/dichloromethane 3:2, v/v to give 1.3 g of the 1,3-alternate isomer **4a** (40%, mp: 248 – 250 °C). ^1H NMR ($CDCl_3$, 400 MHz, 298 K) δ (ppm): 7.98 (s, 4H, Ar-H); 6.98 (s, 4H, Ar-H); 5.82 – 5.73 (m, 2H, -C*H*=CH_2); 5.15 – 5.01 (m, 4H, -CH=CH_2); 4.09 – 4.07 (m, 4H, -O-CH_2-CH=CH_2); 3.84 – 3.79 (m, 4H, Ar-CH_2-Ar); 3.70 – 3.67 (m, 4H, Ar-CH_2-Ar); 3.51 – 3.42 (m, 4H, -O-CH_2-); 1.86 – 1.77 (m, 2H, -CH-); 1.26 – 1.24 (m, 2H, -C*H*H-CH_3); 1.23 (s, 18H, *t*-Bu); 1.09 – 0.97 (m, 2H, -CH*H*-CH_3); 0.84 (t, 6H, *J* = 7.4 Hz, -CH_3); 0.66 (d, 6H, *J* = 6.6 Hz, -CH_3). ^{13}C NMR ($CDCl_3$, 101 MHz, 298 K) δ (ppm): 162.0; 154.8 (2x 2C, Ar-C, C-O-); 144.4 (2C, Ar-C, C-*t*Bu); 141.9 (2C, Ar-C, C-NO_2); 135.7; 135.6 (2x 2C, Ar-C, *C*-CH_2-); 133.8 (2C, -*C*H=CH_2); 131.8; 131.7 (2x 2C, Ar-C, *C*-CH_2-); 127.9; 127.8; 126.1; 125.8 (4x 2C, Ar-CH); 117.5 (2C, -CH=*C*H_2); 77.4; 72.4 (2x 2C, -O-CH_2-); 38.2; 38.0 (2x 2C, Ar-CH_2-Ar); 36.1 (2C, -CH-); 34.0 (2C, -*C*(CH_3)$_3$); 31.6 (6C, -C(*C*H_3)$_3$); 26.6 (2C, -CH_2-); 16.1; 11.6 (2x 2C, -CH_3). HRMS-ESI: ($C_{52}H_{66}N_2O_8$) m/z calcd: 869.4711 $[M+Na]^+$, 885.4451 $[M+K]^+$, found: 869.4713

[M+Na]$^+$, 885.4445 [M+K]$^+$. IR (KBr) ν (cm^{-1}): 3390, 2961, 2916, 2874, 1958, 1518, 1341, 1263. Optical rotation: $[\alpha]^{20}_{589}$ = -13.5° (c = 1.02 g/mL, $CHCl_3$).

Partial-cone 26,28-diallyloxy-11,23-di-*tert*-butyl-25,27-bis[(*S*)-2-methylbutoxy]-5,17-dinitrocalix[4]arene (**4b**)

The calix[4]arene **4b** (in the partial cone conformation) was isolated as the next fraction from the above-described chromatography in the form of a yellowish powder (0.34 g, 10%), mp: 205 – 208 °C. ^{1}H NMR ($CDCl_3$, 400 MHz, 298 K) δ (ppm): 8.26 (s, 2H, Ar-H); 8.04 (s, 2H, Ar-H); 6.89 (d, 2H, J = 2.5 Hz, Ar-H); 6.46 (t, 2H, J = 2.3 Hz, Ar-H); 6.20 – 6.10 (m, 1H, -C*H*=CH$_2$); 5.64 – 5.53 (m, 1H, -C*H*=CH$_2$); 5.43 – 5.31 (m, 2H, -CH=C*H*$_2$); 4.92 – 4.82 (m, 2H, -CH=C*H*$_2$); 4.39 (d, 2H, J = 6.2 Hz, -O-C*H*$_2$-CH=CH$_2$); 4.20 (d, 2H, J = 6.1 Hz, -O-C*H*$_2$-CH=CH$_2$); 4.12 – 4.06 (m, 2H, Ar-CH$_2$-Ar); 3.87 – 3.81 (m, 2H, Ar-CH$_2$-Ar); 3.74 – 3.58 (m, 4H, Ar-CH$_2$-Ar + 2x -O-CH*H*-); 3.42 –3.28 (m, 1H, -O-C*H*H-); 3.32 – 3.26 (m, 1H, -O-CH*H*-); 3.24 – 3.19 (m, 2H, Ar-CH$_2$-Ar); 2.04 – 1.94 (m, 2H, -CH-); 1.71 – 1.61 (m, 2H, -C*H*H-); 1.39 – 1.16 (m, 2H, -CH*H*-); 1.10 (d, 3H, J = 6.7 Hz, -CH$_3$); 1.05 – 1.02 (d, 3H, J = 6.7 Hz, -CH$_3$); 1.02 (m, 21H, *t*-Bu + -CH$_3$); 0.96 (t, 3H, J = 7.4 Hz, -CH$_3$). ^{13}C NMR ($CDCl_3$, 101 MHz, 298 K) δ (ppm): 162.7; 161.7; 154.3; 154.2 (4x C, Ar-C, C-O-); 144.8; 144.7 (2x C, Ar-C, C-*t*-Bu); 142.7; 142.2 (2x C, Ar-C, C-NO$_2$); 138.4; 138.3; 135.6; 135.5 (4x C, Ar-C, *C*-CH$_2$-); 135.0; 134.1 (2x C, -*C*H=CH$_2$); 130.6; 130.5; 130.1; 130.0 (4x C, Ar-C, *C*-CH$_2$-); 126.9; 126.8; 126.7; 126.6; 126.0; 125.9; 124.2; 124.1 (4x C, Ar-CH); 118.8; 116.3 (2x C, -CH=*C*H$_2$); 81.0; 80.9; 74.4; 73.9 (4x C, -O-CH$_2$-); 37.1; 37.0 (2x C, Ar-CH$_2$-Ar); 36.1; 36.0 (2x C, -CH-); 33.9 (2C, -*C*(CH$_3$)$_3$); 31.6 (6C, -C(*C*H$_3$)$_3$); 31.3; 31.2 (2x C, Ar-CH$_2$-Ar); 26.9; 26.7 (2x C, -CH$_2$-); 17.3; 17.2 (2x C, -CH$_3$); 11.5; 11.4 (2x C, -CH$_3$). HRMS-ESI: ($C_{52}H_{66}N_2O_8$) m/z calcd: 869.4711 [M+Na]$^+$, 885.4451 [M+K]$^+$, found: 869.4715 [M+Na]$^+$,

885.4449 $[M+K]^+$. IR (KBr) ν (cm^{-1}): 3390, 2960, 2917, 2850, 1958, 1592, 1518, 1339, 1092. Optical rotation: $[\alpha]^{20}_{589}$ = 4.7° (c = 1.03 g/mL, $CHCl_3$).

1,3-Alternate 26,28-diallyloxy-5,17-diamino-11,23-di-*tert*-butyl-25,27-bis[(*S*)-2-methylbutoxy]calix[4]arene (**5**)

The starting calix[4]arene **4a** (0.8 g, 0.9 mmol) and $SnCl_2{\cdot}2H_2O$ (2.1 g, 9.4 mmol) were dissolved in ethanol (64 mL), and the reaction mixture was heated to reflux for 3 days. The solvent was then evaporated under reduced pressure, and the crude product was dissolved in a mixture of CH_2Cl_2 (62 mL) and a 15% solution of NH_3 (62 mL). The resulting solution was extracted with dichloromethane (3×40 mL), the combined organic layers were washed with a saturated solution of NaCl (50 mL) and water (50 mL) and dried over $MgSO_4$. The solvent was removed on a vacuum evaporator, and the crude mixture was purified by column chromatography on alumina using a cyclohexane/ethyl acetate 20:1, v/v mixture to give 0.42 g (57%) of the final compound **5** (mp 113–116 °C), which was stored refrigerated under an argon atmosphere. ^1H NMR ($CDCl_3$, 400 MHz, 298 K) δ (ppm): 6.91 (s, 4H, Ar-H); 6.41 (s, 4H, Ar-H); 5.75 – 5.66 (m, 2H, -C*H*=CH_2); 5.05 – 4.89 (m, 4H, -CH=CH_2); 3.89 – 3.87 (m, 4H, -O-CH_2-CH=CH_2); 3.68 – 3.56 (m, 8H, Ar-CH_2-Ar); 3.47 – 3.42 (m, 4H, -O-CH_2-); 3.21 (brs, 4H, -NH_2); 1.86 – 1.79 (m, 2H, -CH-); 1.44 – 1.34 (m, 2H, -C*H*H-CH_3); 1.21 (s, 18H, *t*-Bu); 1.13 – 1.01 (m, 2H, -CH*H*-CH_3); 0.93 (t, 6H, J = 7.3 Hz, -CH_3); 0.81 (d, 6H, J = 6.7 Hz, -CH_3). ^{13}C NMR ($CDCl_3$, 101 MHz, 298 K) δ (ppm): 155.2; 149.6 (2x 2C, Ar-C, C-O-); 143.5 (2C, Ar-C, C-*t*-Bu); 139.8 (2C, Ar-C, C-NH_2); 135.6 (2C, -*C*H=CH_2); 134.8; 134.7 (2x 2C, Ar-C, *C*-CH_2-); 132.9 (4C, Ar-C, *C*-CH_2-); 127.2; 127.1; 117.9; 117.7 (4x 2C, Ar-CH); 115.5 (2C, *C*H_2=CH-); 76.6; 72.1 (2x 2C, -O-CH_2-); 38.5; 38.4 (2x 2C, Ar-CH_2-Ar); 36.2 (2C, -CH-); 33.9 (2C, -*C*$(CH_3)_3$); 31.7 (6C, -C(*C*$H_3)_3$); 26.7 (2C, -CH_2-); 16.7; 12.0 (2x 2C, -

CH_3). HRMS-ESI: ($C_{52}H_{70}N_2O_4$) m/z calcd: 787.5408 $[M+H]^+$, 809.5228 $[M+Na]^+$, 825.4967 $[M+K]^+$, found: 787.5411 $[M+H]^+$, 809.5227 $[M+Na]^+$, 825.4957 $[M+K]^+$. IR (KBr) ν (cm^{-1}): 3389, 2956, 2916, 2849, 1958, 1595, 1466, 1094.

1,3-Alternate 5,17-diamino-11,23-di-*tert*-butyl-25,27-bis[(*S*)-2-methylbutoxy]-26,28-dipropoxycalix[4]arene (**6**)

A suspension of the calixarene **4a** (0.1 g, 0.1 mmol) and Pd/C (10%, 0.02 g) in THF (15 mL) was stirred under a H_2 atmosphere (5 bar) in an autoclave at room temperature for 4 days. The catalyst was then removed by filtration through Celite®, and the solution was concentrated under reduced pressure. The crude product was purified by column chromatography on alumina using a cyclohexane/ethyl acetate 10:1, v/v mixture to yield 85 mg (91%) of the final compound **6** (mp: 243–246 °C), which was stored refrigerated under an argon atmosphere. ^1H NMR ($CDCl_3$, 400 MHz, 298 K) δ (ppm): 6.93 (s, 4H, Ar-H); 6.40 (s, 4H, Ar-H); 3.67 – 3.58 (m, 8H, Ar-CH_2-Ar); 3.46 – 3.32 (m, 8H, -O-CH_2-); 3.19 (brs, 4H, -NH_2); 1.78 – 1.68 (m, 2H, -CH-); 1.43 – 1.30 (m, 2H, -C*H*H-CH_3); 1.25 – 1.19 (m, 22H, *t*-Bu + -CH_2-CH_3); 1.08 – 0.99 (m, 2H, -CH*H*-CH_3); 0.90 (t, 6H, J = 7.4 Hz, -CH_3); 0.80 – 0.71 (m, 12H, -CH_3). ^{13}C NMR ($CDCl_3$, 101 MHz, 298 K) δ (ppm): 155.5; 150.1 (2x 2C, Ar-C, C-O-); 143.2 (2C, Ar-C, C-*t*-Bu); 139.7 (2C, Ar-C, C-NH_2); 134.4; 134.3; 132.8; 132.7 (4x 2C, Ar-C, C-CH_2-); 126.5; 126.4 (2x 2C, Ar-CH); 117.5 (4C, Ar-CH); 76.6; 72.9 (2x 2C, -O-CH_2-); 38.5; 38.4 (2x 2C, Ar-CH_2-Ar); 35.9 (2C, -CH-); 33.9 (2C, -*C*(CH_3)$_3$); 31.7 (6C, -C(*C*H_3)$_3$); 26.6; 22.7 (2x 2C, -CH_2-); 16.9; 11.9; 10.3 (3x 2C, -CH_3). HRMS-ESI: ($C_{52}H_{74}N_2O_4$) m/z calcd: 791.5721 $[M+H]^+$, 813.5541 $[M+Na]^+$, 829.5280 $[M+K]^+$, found: 791.5724 $[M+H]^+$, 813.5540 $[M+Na]^+$, 829.5276 $[M+K]^+$. IR (KBr) ν (cm^{-1}): 3360, 2958, 2919, 2872, 1617, 1468, 1230.

Synthesis of ureido derivatives–general procedure

The corresponding isocyanate (10 equiv) was added to a solution of the calix[4]arene **5** or **6** (0.03 g) in 10 mL of dry dichloromethane under an argon atmosphere. The mixture was stirred for 3 to 5 days at room temperature. The reaction was quenched by the addition of 10 mL of methanol, and the reaction mixture was stirred for 30 minutes. The solvent was then evaporated under reduced pressure. The particular purification methods of the crude product are described below for each individual receptor.

1,3-Alternate 26,28-diallyloxy-11,23-di-*tert*-butyl-25,27-bis[(*S*)-2-methylbutoxy]-5,17-bis[*N*´-(4-nitrophenyl)ureido]-calix[4]arene (**7a**): Compound **7a** was prepared according to the general procedure using the calixarene **5** (0.030 g, 0.040 mmol) and *p*-nitrophenyl isocyanate (0.02 g, 0.40 mmol). The title compound **7a** was obtained without chromatography, by multiple triturations of the crude solid in methanol, in the form of a yellow powder (0.020 g, 34%), mp: 180 -182 °C. ^{1}H NMR (DMSO-d_6, 400 MHz, 298 K) δ (ppm): 9.33 (s, 2H, -NH-CO); 8.30 (s, 2H, -NH-CO); 8.15 (d, 4H, J = 9.3 Hz, Ar-H); 7.66 (d, 4H, J = 9.3 Hz, Ar-H); 7.23 (d, 2H, J = 2.5 Hz, Ar-H); 7.10 (d, 2H, J = 2.5 Hz, Ar-H); 6.94 (s, 4H, Ar-H); 5.53 (m, 2H, -C*H*=CH$_2$); 4.95-4.81 (m, 4H, -CH=C*H*$_2$); 3.71 – 3.66 (m, 12H, Ar-CH$_2$-Ar + -O-CH$_2$-); 3.30 – 3.25 (m, 4H, -O-CH$_2$-); 1.72 – 1.68 (m, 2H, -CH-); 1.27 – 1.24 (m, 2H, -C*H*H-); 1.19 (s, 18H, *t*-Bu); 0.9 (m, 2H, -CH*H*-); 0.75 (t, 6H, J = 7.4 Hz, -CH$_3$); 0.60 (d, 6H, J = 6.6 Hz, -CH$_3$). ^{13}C NMR (DMSO-d_6, 101 MHz, 298 K) δ (ppm): 154.8; 151.6 (2x 2C, Ar-C$_{calix}$, *C*-OCH$_2$-); 151.4 (2C, C=O); 146.6 (2C, Ar-C, C-NO$_2$); 142.6 (2C, Ar-C$_{calix}$, C–*t*-Bu); 140.6 (2C, Ar-C, C-NH-); 135.2 (2C, CH$_2$=*C*H-); 134.2 (2C, Ar-C$_{calix}$, C-NH-); 134.1; 132.6; 132.5; 132.4 (4x 2C, Ar- C$_{calix}$, *C*-CH$_2$-); 126.7; 126.6 (2x 2C, Ar-CH$_{calix}$, *C*H-C-*t*-

Bu); 125.1 (4C, Ar-CH, *C*H-C-NO_2); 119.9; 119.8 (2x 2C, Ar-CH_{calix}, *C*H-C-NH); 117.0 (4C, Ar-CH, *C*H-CH-C-NO_2); 114.8 (2C, *C*H_2=CH-); 76.0; 70.8 (2x 2C, -O-CH_2-); 38.2; 38.0 (2x 2C, Ar-CH_2-Ar); 34.8 (2C, -CH-); 33.5 (2C, -*C*(CH_3)$_3$); 31.3 (6C, -C(*C*H_3)$_3$); 25.9 (2C, -CH_2-); 16.2; 11.3 (2x 2C, -CH_3). HRMS-ESI: ($C_{66}H_{78}N_6O_{10}$) m/z calcd: 1137.5672 $[M+Na]^+$, 1153.5411 $[M+K]^+$, found: 1137.5671 $[M+Na]^+$, 1153.5410 $[M+K]^+$. IR (KBr) ν (cm^{-1}): 3600, 3308, 3091, 2962, 2874, 1726, 1549, 1476, 1419, 1325, 1302, 1197. Optical rotation: $[\alpha]_{589}^{20}$= -33.9° (c = 0.01 g/mL, acetone).

1,3-Alternate 26,28-diallyloxy-11,23-di-*tert*-butyl-5,17-bis[*N*´-(4-butyl)ureido]-25,27-bis[(*S*)-2-methylbutoxy]calix[4]arene (**7b**): Compound **7b** was prepared according to the general procedure using the calixarene **5** (0.060 g, 0.070 mmol) and *p*-butylphenyl isocyanate (0.10 mL, 0.70 mmol). The crude mixture was purified by multiple triturations in methanol to obtain the final product **7b** as a slightly pink powder (0.04 g, 49%), mp: 239 – 242 °C. ^{1}H NMR (DMSO-*d*$_6$, 400 MHz, 298 K) δ (ppm): 8.45 (s, 2H, -NH-CO); 7.97 (s, 2H, -NH-CO); 7.31 (d, 4H, *J* = 8.5 Hz, Ar-H); 7.17 (d, 2H, *J* = 2.6 Hz, Ar-H); 7.05 (m, 6H, Ar-H); 6.93 (s, 4H, Ar-H); 5.57 – 5.47 (m, 2H, -C*H*=CH_2); 4.93 – 4.83 (m, 4H, -CH=C*H*$_2$); 3.73 – 3.62 (m, 12H, -O-CH_2-, Ar-CH_2-Ar); 3.32 – 3.23 (m, 4H, -O-CH_2-); 2.53 (m, overlapped with solvent, 4H, -CH_2-); 1.75 – 1.66 (m, 2H, -CH-); 1.54 – 1.47 (m, 4H, -CH_2-); 1.31 – 1.24 (m, 4H, -CH_2-); 1.19 (m, 20H, *t*-Bu, -C*H*H-); 0.92 – 0.88 (m, 2H, -CH*H*-); 0.88 (t, 6H, *J* = 7.3 Hz -CH_3); 0.77 (t, 6H, *J* = 7.3 Hz -CH_3); 0.61 (d, 6H, *J* = 6.6 Hz, -CH_3). ^{13}C NMR (DMSO-*d*$_6$, 101 MHz, 298 K) δ (ppm): 154.8; 152.5 (2x 2C, Ar-C, *C*-O-CH_2-); 150.9 (2C, C=O); 142.5 (2C, Ar- C_{calix}, C–*t*-Bu); 137.7 (2C, Ar-C, C-Bu); 135.3 (2C, CH_2=*C*H-); 135.2 (2C, Ar-C, C-NH-); 133.99; 133.91 (2x 2C, Ar-C_{calix}, *C*-CH_2-); 133.3 (2C, Ar-C_{calix}, C-NH-); 132.6; 132.5 (2x 2C, Ar-C_{calix}, *C*-CH_2-); 128.5 (4C, Ar-CH, -*C*H-C-Bu); 126.6; 126.5; 119.7;

119.5 (4x 2C, Ar-CH_{calix}); 117.9 (4C, Ar-CH, -*C*H-C-NH-); 114.7 (2C, *C*H_2=CH-); 76.0; 70.9 (2x 2C, -O-CH_2-); 38.3; 38.1 (2x 2C, Ar-CH_2-Ar); 34.8 (2C, -CH-); 34.1 (2C, -CH_2-); 33.5 (2C, -*C*(CH_3)$_3$); 33.3 (2C, -CH_2-); 31.3 (6C, -C(*C*H_3)$_3$); 25.9; 21.6 (2x 2C, -CH_2-); 16.2; 13.8; 11.4 (3x 2C, -CH_3). HRMS-ESI: ($C_{74}H_{96}N_4O_6$) m/z calcd: 1159.7222 $[M+Na]^+$, 1175.6961 $[M+K]^+$, found: 1159.7237 $[M+Na]^+$, 1175.6969 $[M+K]^+$. IR (KBr) ν (cm^{-1}): 3344, 2955, 2926, 2871, 1661, 1543, 1475, 1418, 1218, 1197. Optical rotation: $[\alpha]_{589}^{20}$ = -40.0° (c = 0.01 g/mL, acetone).

1,3-Alternate 26,28-diallyloxy-11,23-di-*tert*-butyl-5,17-bis[*N*´-(4-(*S*)-1-methylbenzyl)ureido]-25,27-bis[(*S*)-2-methylbutoxy]calix[4]arene (**7c**): Compound **7c** was prepared according to the general procedure using the calixarene **5** (0.50 g, 0.60 mmol) and (*S*)-1-phenylethyl isocyanate (0.5 mL, 3.2 mmol). The crude mixture was purified by multiple triturations in methanol to obtain the final product **7c** as pink microcrystals (0.35 g, 53%), mp: 240 – 242 °C. ^{1}H NMR (400 MHz, DMSO-*d*$_6$, 298 K) δ (ppm): 7.73 (s, 2H, -NH-CO); 7.33 – 7.30 (m, 8H, Ar-H); 7.25 – 7.22 (m, 2H, Ar-H); 7.06 (m, 2H, Ar-H_{calix}); 6.98 (m, 2H, Ar-H_{calix}); 6.89 (s, 4H, Ar-H_{calix}); 6.50 (d, 2H, *J* = 7.9 Hz, -NH-CO); 5.53 – 5.46 (m, 2H, CH_2=C*H*-); 4.93 – 4.74 (m, 6H, C*H*$_2$=CH- + -C*H*-NH-); 3.72 – 3.46 (m, 12H, Ar-CH_2-Ar + -O-CH_2-$_{allyl}$); 3.27 – 3.09 (m, 4H, -O-CH_2-); 1.68 – 1.58 (m, 2H, -O-CH_2-C*H*-); 1.38 (d, 6H, *J* = 6.9 Hz, C*H*$_3$-CH-NH); 1.17 (m, 20H, -C(CH_3)$_3$ + -C*H*H -CH_3); 0.87 – 0.84 (m, 2H –CH*H*-CH_3); 0.76 (t, 6H, *J* = 7.3 Hz, C*H*$_3$-CHH-); 0.55 (d, 6H, *J* = 6.6 Hz, C*H*$_3$-CH-). ^{13}C NMR (DMSO-*d*$_6$, 101 MHz, 298 K) δ (ppm): 154.8 (2C, Ar C_{calix}, *C*-OCH_2-); 154.5 (2C, C=O); 150.4 (2C, Ar C_{calix}, *C*-OCH_2-); 145.4 (2C, Ar-C, *C*-CH); 142.4 (2C, Ar- C_{calix}, C-t-Bu); 135.4 (2C, CH_2=*C*H-); 133.8 (2C, Ar-C_{calix}, C-NH-); 133.7; 133.6; 132.65; 132.62 (4x 2C, Ar-C_{calix}, *C*-CH_2); 128.2 (4C, Ar-CH); 126.7; 126.6 (4C, Ar-CH_{calix}, *t*-Bu, 2C Ar-CH); 125.8 (4C, Ar-CH); 119.3; 119.2 (2x 2C, Ar-CH_{calix}, *C*H-C-NH); 114.5 (2C, *C*H_2=CH-); 75.9; 70.8 (2x 2C, -O-

CH_2-); 48.4 (2C, CH-NH-), 38.3; 38.2 (2x 2C, Ar-CH_2-Ar); 34.7 (2C, -CH-); 33.4 (2C, -*C*(CH_3)$_3$); 31.4 (6C, -C(*C*H_3)$_3$); 25.8 (2C, -CH_2-); 22.9 (2C, *C*H_3-CH-NH-); 16.1; 11.4 (2x 2C, -CH_3). HRMS-ESI: ($C_{70}H_{88}N_4O_6$) m/z calcd: 1103.6596 $[M+Na]^+$, 1119.6335 $[M+K]^+$, found: 1103.6605 $[M+Na]^+$, 1119.6333 $[M+K]^+$. IR (KBr) ν (cm^{-1}): 3329, 2958, 2925, 2871, 1647, 1546, 1475, 1200. Optical rotation: $[\alpha]_{589}^{20}$ = -43.6° (c = 0.01 g/mL, acetone).

1,3-Alternate 26,28-diallyloxy-11,23-di-*tert*-butyl-5,17-bis[*N*´-(4-(*S*)-1-methylbenzyl)ureido]-25,27-bis[(*R*)-2-methylbutoxy]calix[4]arene (**7d**): Compound **7d** was prepared according to the general procedure using the calixarene **5** (0.20 g, 0.20 mmol) and (*R*)-1-phenylethyl isocyanate (0.3 mL, 1.9 mmol). The crude mixture was purified by multiple triturations in methanol to obtain the final product **7d** as a slightly pink powder (0.12 g, 61%), mp: 239 – 242 °C. ^{1}H NMR (400 MHz, DMSO-d_6, 298 K) δ (ppm): 7.73 (s, 2H, -NH-CO); 7.32 – 7.30 (m, 8H, Ar-H); 7.25 – 7.19 (m, 2H,, Ar-H); 7.06 (m, 2H, Ar-H_{calix}); 6.98 (m, 2H, Ar-H_{calix}); 6.89 (s, 4H, Ar-H_{calix}); 6.50 (d, 2H, *J* = 7.9 Hz,- NH-CO); 5.53 – 5.46 (m, 2H, CH_2=C*H*-); 4.93 – 4.74 (m, 6H, C*H*$_2$=CH- + -C*H*-NH-); 3.67 – 3.45 (m, 12H, Ar-CH_2-Ar + -O-CH_2-$_{allyl}$); 3.24 – 3.15 (m, 4H, -O-CH_2-); 1.68 – 1.58 (m, 2H, -O-CH_2-C*H*-); 1.38 (d, 6H, *J* = 6.9 Hz, C*H*$_3$-CH-NH-); 1.17 (m, 20H, -C(CH_3)$_3$ + -C*H*H -CH_3); 0.88 – 0.76 (m, 2H -CH*H*-CH_3); 0.72 (t, 6H, *J* = 7.3 Hz, C*H*$_3$-CHH); 0.57 (d, 6H, *J* = 6.6 Hz, C*H*$_3$-CH-). ^{13}C NMR (DMSO-d_6, 101 MHz, 298 K) δ (ppm): 154.8 (2C, Ar-C_{calix}, *C*-OCH_2-); 154.5 (2C, C=O); 150.4 (2C, Ar-C_{calix}, *C*-O-CH_2-); 145.4 (2C, Ar-C, *C*-CH); 142.5 (2C, Ar- C_{calix}, *C*-*t*-Bu); 135.4 (2C, CH_2=*C*H-); 133.9 (2C, Ar-C_{calix}, *C*-NH-); 133.8; 133.7; 132.7; 132.6 (4x 2C, Ar- C_{calix}, *C*-CH_2); 128.2 (4C, Ar-CH); 126.6; 126.5 (2x 2C, Ar-CH_{calix}, 2C, Ar-CH); 125.8 (4C, Ar-CH); 119.3; 119.2 (2x 2C, Ar-CH_{calix}, *C*H-C-NH); 114.6 (2C, *C*H_2=CH-); 76.0; 70.9 (2x 2C, -O-CH_2-); 48.4 (2C, CH-NH-); 38.3; 38.2 (2x 2C, Ar-CH_2-Ar); 34.8 (2C, -CH-); 33.5 (2C, -

C(CH_3)$_3$); 31.4 (6C, -C(*C*H_3)$_3$); 25.9 (2C, -CH_2-); 23.0 (2C, *C*H_3-CH-NH); 16.1; 11.4 (2x 2C, CH_3-). HRMS-ESI: ($C_{70}H_{88}N_4O_6$) m/z calcd: 1081.6777 $[M+H]^+$, 1103.6596 $[M+Na]^+$, 1119.6335 $[M+K]^+$, found: 1081.6779 $[M+H]^+$, 1103.6603 $[M+Na]^+$, 1119.6331 $[M+K]^+$. IR (KBr) ν (cm^{-1}): 3307, 2957, 2898, 2866, 1650, 1552, 1476, 1199. Optical rotation: $[\alpha]_{589}^{20}$ = 11.1° (c = 0.01 g/mL, acetone).

1,3-Alternate 11,23-di-*tert*-butyl-25,27-bis[(*S*)-2-methylbutoxy]-5,17-bis[*N*´-(4-nitrophenyl)ureido]-26,28-dipropoxycalix[4]arene (**8a**): Compound **8a** was prepared according to the general procedure using the calixarene **6** (0.060 g, 0.070 mmol) and *p*-nitrophenyl isocyanate (0.1 g, 0.70 mmol). The crude mixture was crystallised from ethyl acetate to form **8a** (0.03 g, 38%) as yellow crystals, mp: 218 – 221 °C. ^{1}H NMR (DMSO-*d*$_6$, 400 MHz, 298 K) δ (ppm): 9.31 (s, 2H, -NH-CO-); 8.34 (s, 2H, -NH-CO-); 8.15 (d, 4H, *J* = 7.3 Hz, Ar-H); 7.66 (d, 4H, *J* = 7.4 Hz, Ar-H); 7.20 (m, 2H, Ar-H); 7.03 (m, 2H, Ar-H); 6.98 (s, 4H, Ar-H); 3.80 – 3.69 (m, 8H, Ar-CH_2-Ar); 3.33 (m, solvent overlapped, 2H, -O-C*H*H); 3.24 – 3.16 (m, 6H, -O-CH_2-, -O-CH*H*); 1.53 – 1.44 (m, 2H, -CH-); 1.22 (s, 18H, *t*-Bu, + m, 2H, -C*H*H-CH_3); 0.99 – 0.90 (m, 4H, -CH_2-); 0.87 – 0.79 (m, 2H, -CH*H*-CH_3); 0.73 (t, 6H, *J* = 7.3 Hz, -CH_3); 0.63 (t, 6H, *J* = 7.5 Hz, -CH_3); 0.52 (d, 6H, *J* = 6.6 Hz, -CH_3). ^{13}C NMR (DMSO-*d*$_6$, 101 MHz, 298 K) δ (ppm): 155.0; 152.0 (2x 2C, Ar C_{calix}, *C*-OCH_2-); 151.7 (2C, C=O); 146.7 (2C, Ar-C, C-NO_2); 142.4 (2C, Ar- C_{calix}, C–*t*-Bu); 140.6 (2C, Ar-C, C-NH-); 133.6 (2C, Ar-C_{calix}, C-NH-); 132.4 (4C, Ar- C_{calix}, *C*-CH_2-); 132.3; 132.2 (2x 2C, Ar- C_{calix}, *C*-CH_2-); 125.8; 125.6 (2x 2C, Ar-CH_{calix}, *C*H-C-*t*-Bu); 125.1 (4C, Ar-CH, *C*H-C-NO_2); 119.4; 119.3 (2x 2C, Ar-CH_{calix}, *C*H-C-NH); 117.1 (4C, ArCH, *C*H-CH-C-NO_2); 75.7; 71.2 (2x 2C, -O-CH_2-); 38.3; 38.1 (2x 2C, Ar-CH_2-Ar); 34.5 (2C, -CH-); 33.5 (2C, -*C*(CH_3)$_3$); 31.3 (6C, -C(*C*H_3)$_3$); 25.8; 21.5 (2x 2C, -CH_2-); 16.5; 11.3; 9.8 (3x2C, -CH_3). HRMS-ESI: ($C_{66}H_{82}N_6O_{10}$) m/z

calcd: 1141.5985 [M+Na]$^+$, 1157.5724 [M+K]$^+$, found: 1141.5978 [M+Na]$^+$, 1157.5713 [M+K]$^+$. IR (KBr) ν (cm^{-1}): 3368, 2959, 2917, 2849, 1958, 1563, 1331, 1111. Optical rotation: $[\alpha]_{589}^{20}$ = 25.9° (c = 0.01 g/mL, acetone).

1,3-Alternate 11,23-di-*tert*-butyl-5,17-bis[*N´*-(4-butyl)ureido]-25,27-bis[(*S*)-2-methylbutoxy]-26,28-dipropoxycalix[4]arene (**8b**): Compound **8b** was prepared according to the general procedure using the calixarene **6** (0.30 g, 0.30 mmol) and *p*-butylphenyl isocyanate (0.56 mL, 3.16 mmol). The crude mixture was purified by repeated trituration to yield **8b** (45%) in the form of a pinkish powder, mp: 236 – 238 °C. ^{1}H NMR (DMSO-d_6, 400 MHz, 298 K) δ (ppm): 8.43 (s, 2H, -NH-CO); 7.99 (s, 2H, -NH-CO); 7.31 (d, 4H, J = 8.5 Hz, Ar-H); 7.13 (d, 2H, J = 2.6 Hz, Ar-H); 7.05 (d, 4H, J = 8.5 Hz, Ar-H); 6.98 (m, 6H, Ar-H); 3.79 – 3.60 (m, 8H, Ar-CH_2-Ar); 3.31 – 3.29 (m, 2H, -O-C*H*H-); 3.23 – 3.15 (m, 6H, -O-CH_2-, -O-CH*H*-); 2.53 (m, overlapped with solvent, 4H, -CH_2-); 1.54 – 1.50 (m, 6H, -CH_2-, -CH-); 1.28 (m, 4H, -CH_2-); 1.22 (s, 18H, *t*-Bu); 1.20 – 1.18 (m, 2H, -C*H*H-); 0.96 (m, 6H, -CH*H*-, -CH_2-); 0.88 (t, 6H, J = 7.3 Hz, -CH_3); 0.74 (t, 6H, J = 7.4 Hz, -CH_3); 0.62 (t, 6H, J = 7.5 Hz, -CH_3); 0.52 (d, 6H, J = 6.6 Hz, -CH_3). ^{13}C NMR (DMSO-d_6, 101 MHz, 298 K) δ (ppm): 155.1; 152.5 (2x 2C, Ar-C, *C*-O-CH_2-); 151.5 (2C, C=O); 142.3 (2C, Ar- C_{calix}, C–*t*-Bu); 137.7 (2C, Ar-C, C-Bu); 135.2 (2C, Ar-C, C-NH-); 133.4 (4C, Ar-C_{calix}, *C*-CH_2-); 133.1 (2C, Ar-C_{calix}, C-NH-); 132.4; 132.3 (2x 2C, Ar-C_{calix}, *C*-CH_2-); 128.5 (4C, Ar-CH, -*C*H-C-Bu); 125.8; 125.6; 119.2; 119.1 (4x 2C, Ar-CH_{calix}); 117.9 (4C, Ar-CH, -*C*H-C-NH-); 75.7; 71.3 (2x 2C, -O-CH_2-); 38.4; 38.2 (2x 2C, Ar-CH_2-Ar); 34.5 (2C, -CH-); 34.1 (2C, -CH_2-); 33.5 (2C, -*C*(CH_3)$_3$); 33.3 (2C, -CH_2-); 31.3 (6C, -C(*C*H_3)$_3$); 25.8; 21.6; 21.5 (3x 2C, -CH_2-); 16.5; 13.8; 11.3; 9.9 (4x 2C, -CH_3). HRMS-ESI: ($C_{74}H_{100}N_4O_6$) m/z calcd: 1163.7535 [M+Na]$^+$, 1179.7275

$[M+K]^+$, found: 1163.7542 $[M+Na]^+$, 1179.7273 $[M+K]^+$. IR (KBr) ν (cm^{-1}): 3331, 2957, 2928, 2871, 1664, 1542, 1464, 1310, 1221. Optical rotation: $[\alpha]_{589}^{20}$ = 5.8° (c = 0.01 g/mL, acetone).

X-ray measurements

Crystallographic data for **4a**

M = 878.14 g·mol^{-1}, tetragonal system, space group $P4_12_12$, a = 13.7561 (3) Å, c = 52.9108 (11) Å, Z = 8, V = 10012 (5) Å^3, D_c = 1.165 g·cm^{-3}, μ(Cu-Kα) = 0.63 mm^{-1}, crystal dimensions of 0.53×0.49×0.41 mm (crystallised from MeOH). Data were collected at 180 (2) K on a D8 Venture Photon CMOS diffractometer with Incoatec microfocus sealed tube Cu-Kα radiation. The structure was solved by charge flipping methods [1] and anisotropically refined by full matrix least squares on F squared using the CRYSTALS suite off programs [2] to final value R = 0.049 and wR = 0.150 using 9840 independent reflections (θ_{max} = 72.1°), 692 parameters and 177 restrains. The hydrogen atoms bonded to carbon atoms were placed in calculated positions refined with a riding constrains. The disordered functional groups positions were found in difference electron density maps and refined with restrained geometry. MCE [3] was used for visualisation of electron density maps. The occupancies of disordered functional groups were constrained to full. The hydroxyl hydrogen atoms of disordered methanol could not be located in difference electron density maps; therefore, they are absent in the structure model. The absolute configuration was unambiguously assigned with resulting Flack parameter −0.06(2). The structure was deposited into Cambridge Structural Database under number CCDC 2027211.

Crystallographic data for **7a**

M = 679.90 g·mol^{-1}, monoclinic system, space group $C2$, a = 22.9527 (9) Å, b = 13.2541 (5) Å, c = 44.4840 (16) Å, β = 90.025 (1)°, Z = 8, V = 13532.8 (9) Å^3, D_c = 1.248 g·cm^{-3}, μ(Cu-Kα) = 1.24 mm^{-1}, crystal dimensions of 0.38×0.26×0.16 mm (crystallised from DMSO). Data were collected at 120 (2) K on a D8 Venture Photon CMOS diffractometer with Incoatec microfocus sealed tube Cu-Kα radiation. The structure was solved by direct methods [4] and anisotropically refined by full matrix least squares on F squared using the CRYSTALS suite of programs [2] to final value R = 0.045 and wR = 0.129 using 25224 independent reflections (θ_{max} = 72.3°), 1835 parameters and 281 restrains. The hydrogen atoms bonded to carbon were placed in calculated positions and refined with a riding constrains. The hydrogen atoms bonded to nitrogen were found in difference electron density maps and refined with restrained bond lengths. The disordered functional groups positions were found in difference electron density maps and refined with restrained geometry. MCE [3] was used for visualisation of electron density maps. The occupancies of disordered functional groups were constrained to full. The absolute configuration was unambiguously assigned with resulting Flack parameter 0.081(3). The structure was deposited into Cambridge Structural Database under number 2027212.

Crystallographic data for **7d**

M = 1255.73 g·mol^{-1}, triclinic system, space group $P1$, a = 9.5703 (3) Å, b = 12.5633 (3) Å, c = 16.4766 (4) Å, α = 75.1716 (10)°, β = 82.0204 (10)°, γ = 76.1604 (10)°, Z = 1, V = 1853.12 (9) Å^3, D_c = 1.125 g·cm^{-3}, μ(Cu-Kα) = 0.57 mm^{-1}, crystal dimensions of 0.47×0.42×0.40 mm (crystallised from acetone). Data were collected at 220 (2) K on a D8 Venture

Photon CMOS diffractometer with Incoatec microfocus sealed tube Cu-Kα radiation. The structure was solved by direct methods [4] and anisotropically refined by full matrix least squares on F squared using the CRYSTALS suite of programs [2] to final value R = 0.037 and wR = 0.109 using 13036 independent reflections (θ_{max} = 68.2°), 905 parameters and 79 restrains. The hydrogen atoms bonded to carbon were placed in calculated positions and refined with a riding constrains. The hydrogen atoms bonded to nitrogen were found in difference electron density maps and refined with restrained bond lengths. The disordered functional groups positions were found in difference electron density maps and refined with restrained geometry. MCE [3] was used for visualisation of electron density maps. The occupancies of disordered functional groups were constrained to full. The absolute configuration was unambiguously assigned with resulting Flack parameter 0.05(2). The structure was deposited into Cambridge Structural Database under number CCDC 2027213.

References

1. Palatinus, L.; Chapuis, G. *J. Appl. Cryst.* **2007**, *40*, 786−790.
2. Betteridge, P. W.; Carruthers, J. R.; Cooper, R. I.; Prout, K.; Watkin, D. J. *J. Appl. Cryst*. **2003**, *36*, 1487.
3. Rohlicek, J.; Husak, M. *J. Appl. Cryst*. **2007**, *40*, 600.
4. Sheldrick, G. M. *Acta Cryst.* **2015**, *A71*, 3−8.

Spectral characterisation of compounds

Compound 2

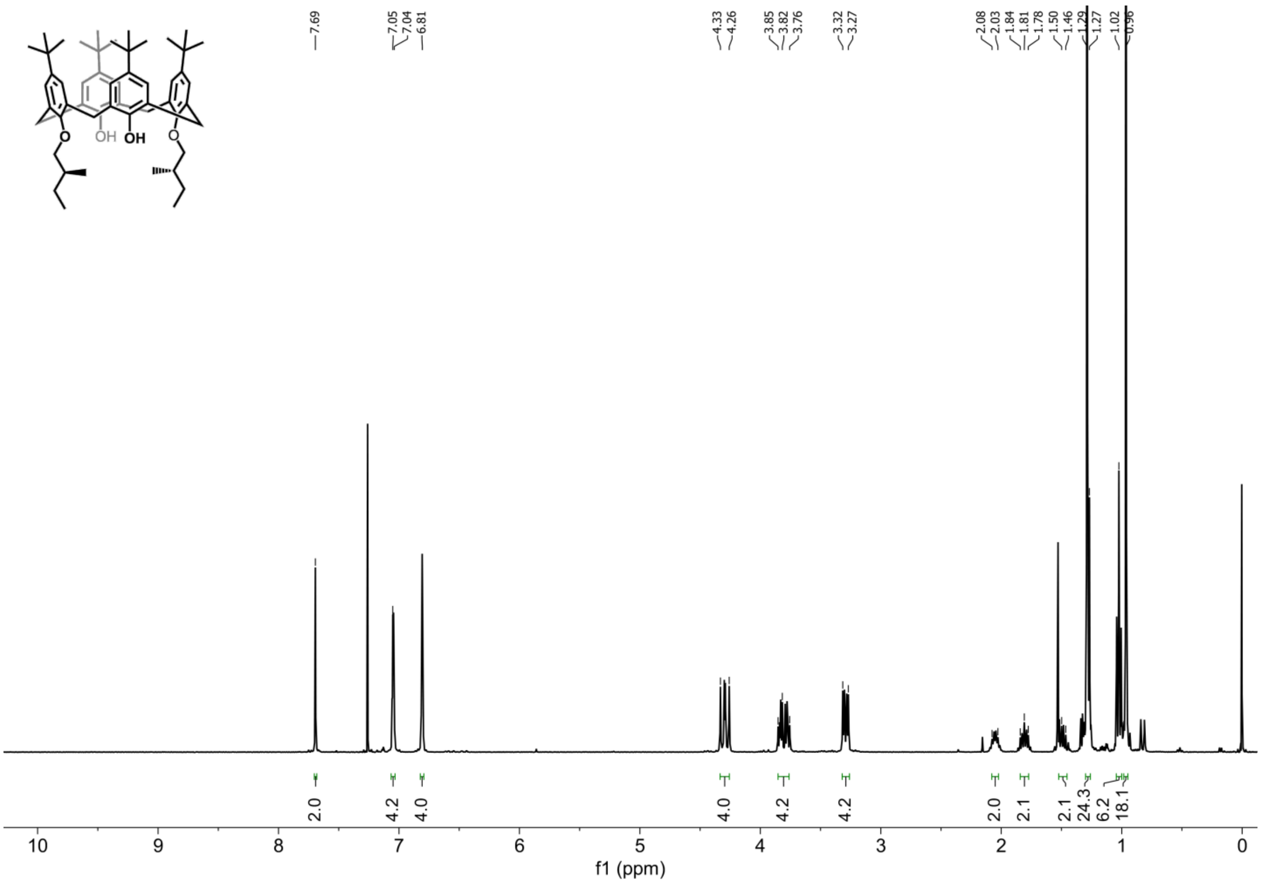

Figure 1. ^{1}H NMR of compound **2** ($CDCl_3$, 400.1 MHz, 298 K).

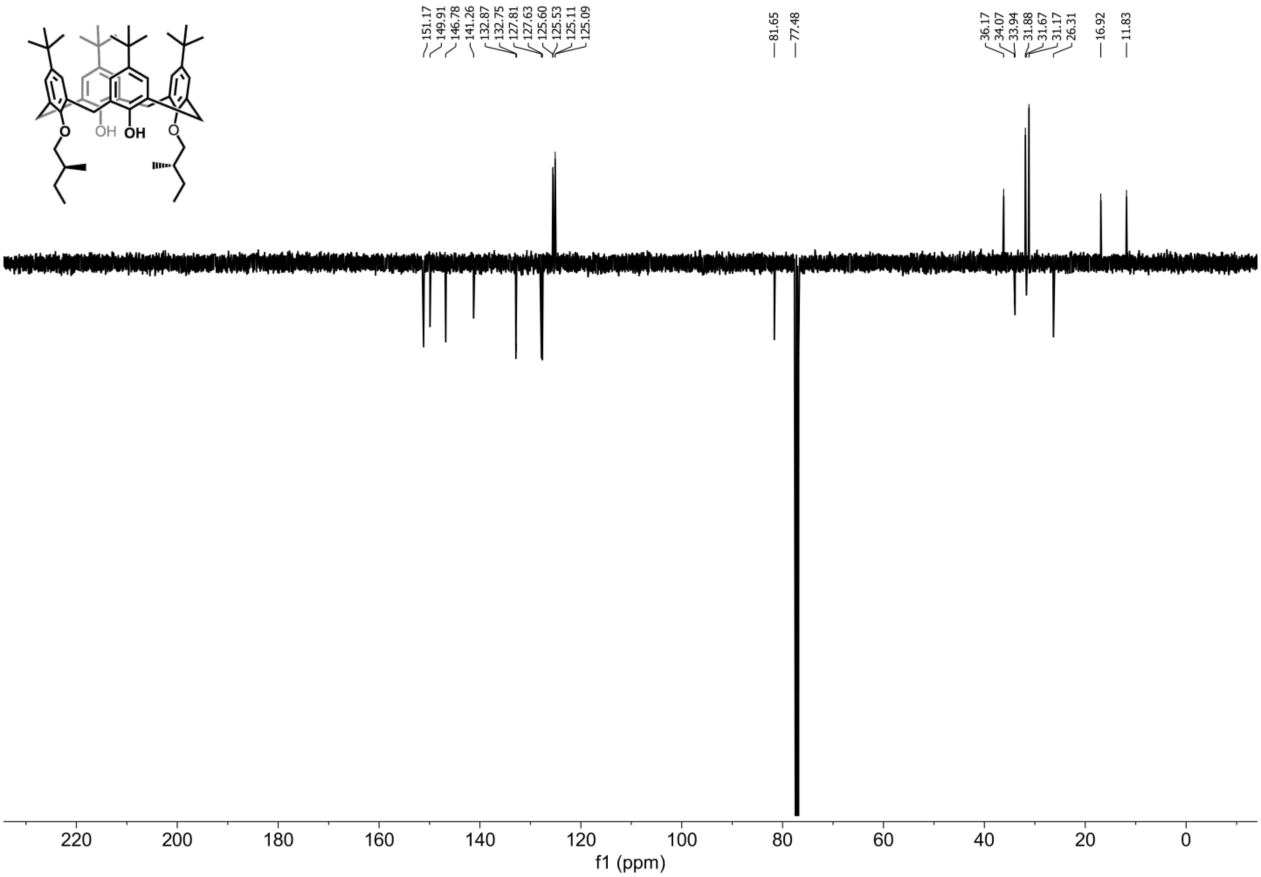

Figure 2. ^{13}C NMR (APT) of compound **2** ($CDCl_3$, 100.6 MHz, 298 K).

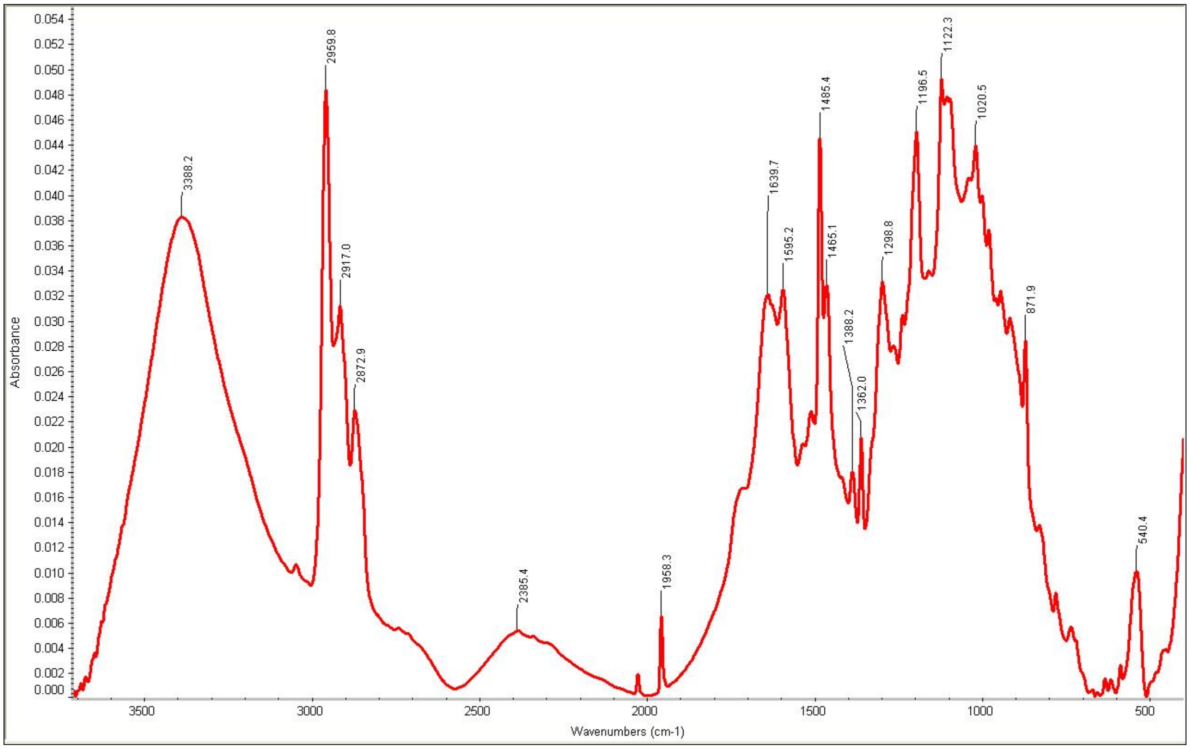

Figure 3. IR of compound **2** (KBr).

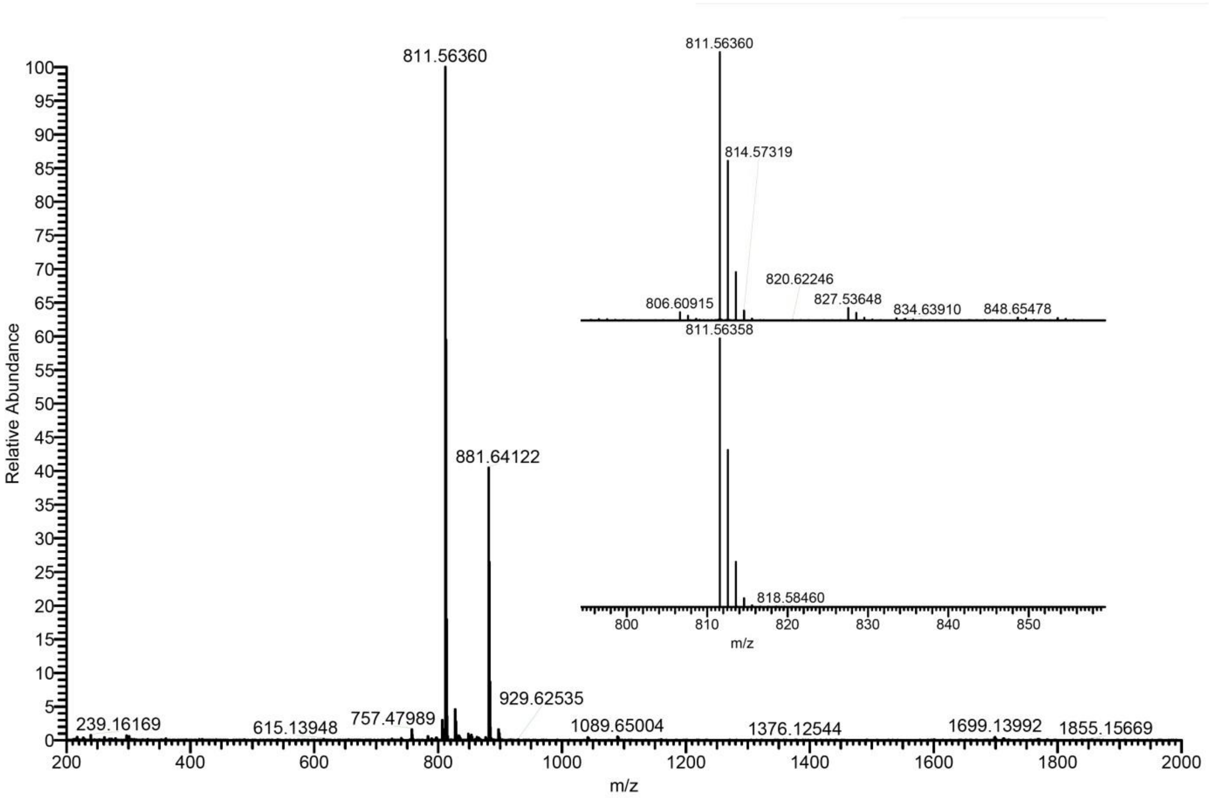

Figure 4. HRMS-ESI of compound **2** (($C_{54}H_{76}O_4$) m/z calcd: 811.5636 $[M+Na]^+$).

Compound 3

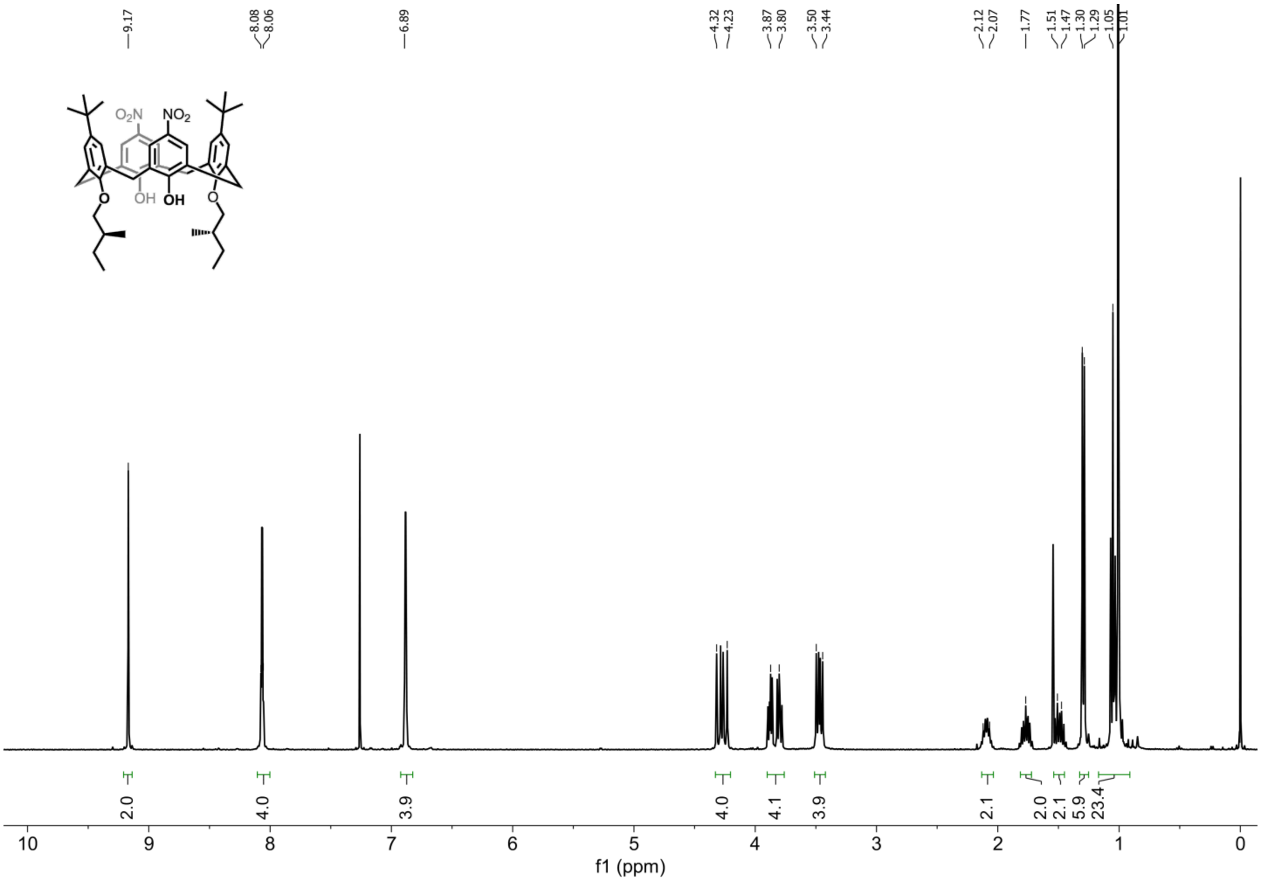

Figure 5. ^{1}H NMR of compound **3** ($CDCl_3$, 400.1 MHz, 298 K).

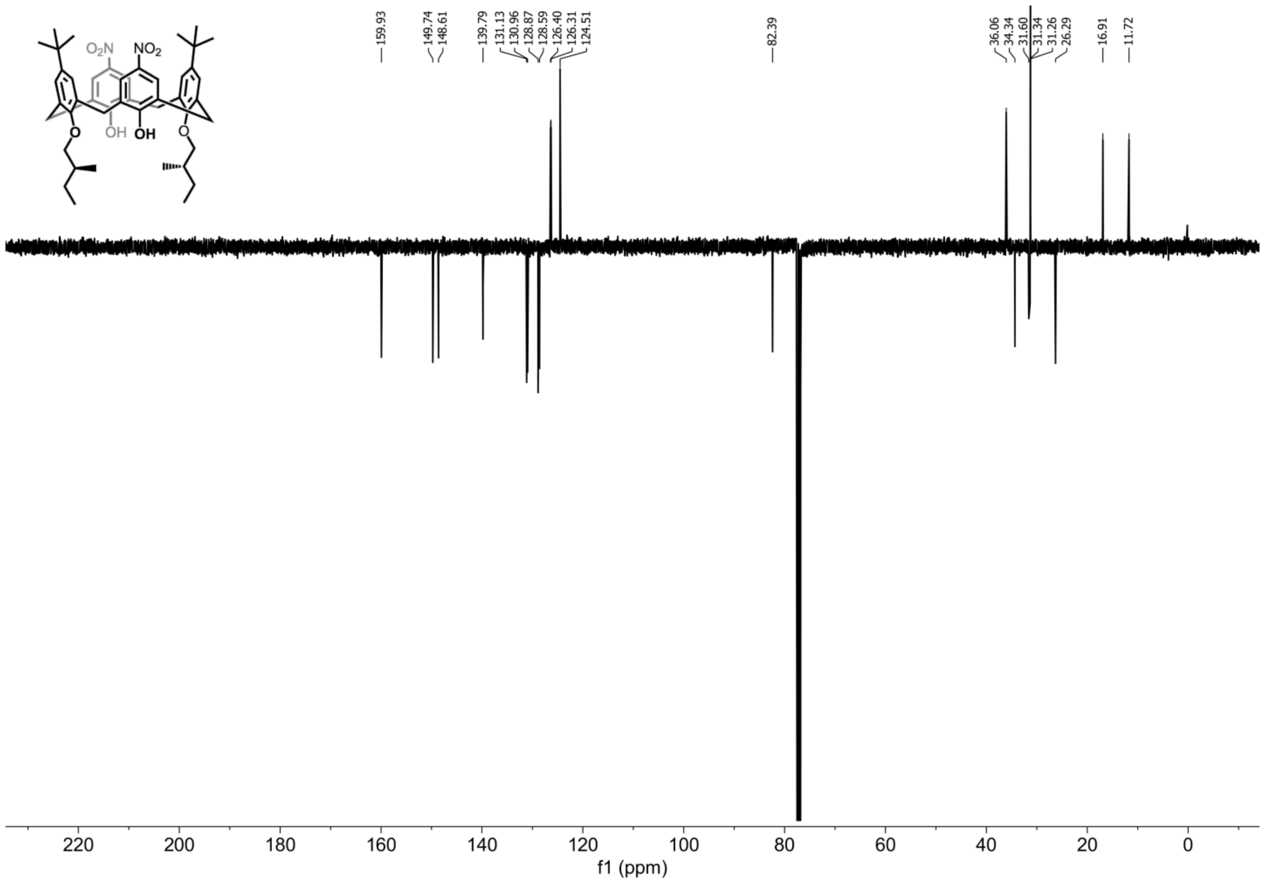

Figure 6. ^{13}C NMR (APT) of compound **3** ($CDCl_3$, 100.6 MHz, 298 K).

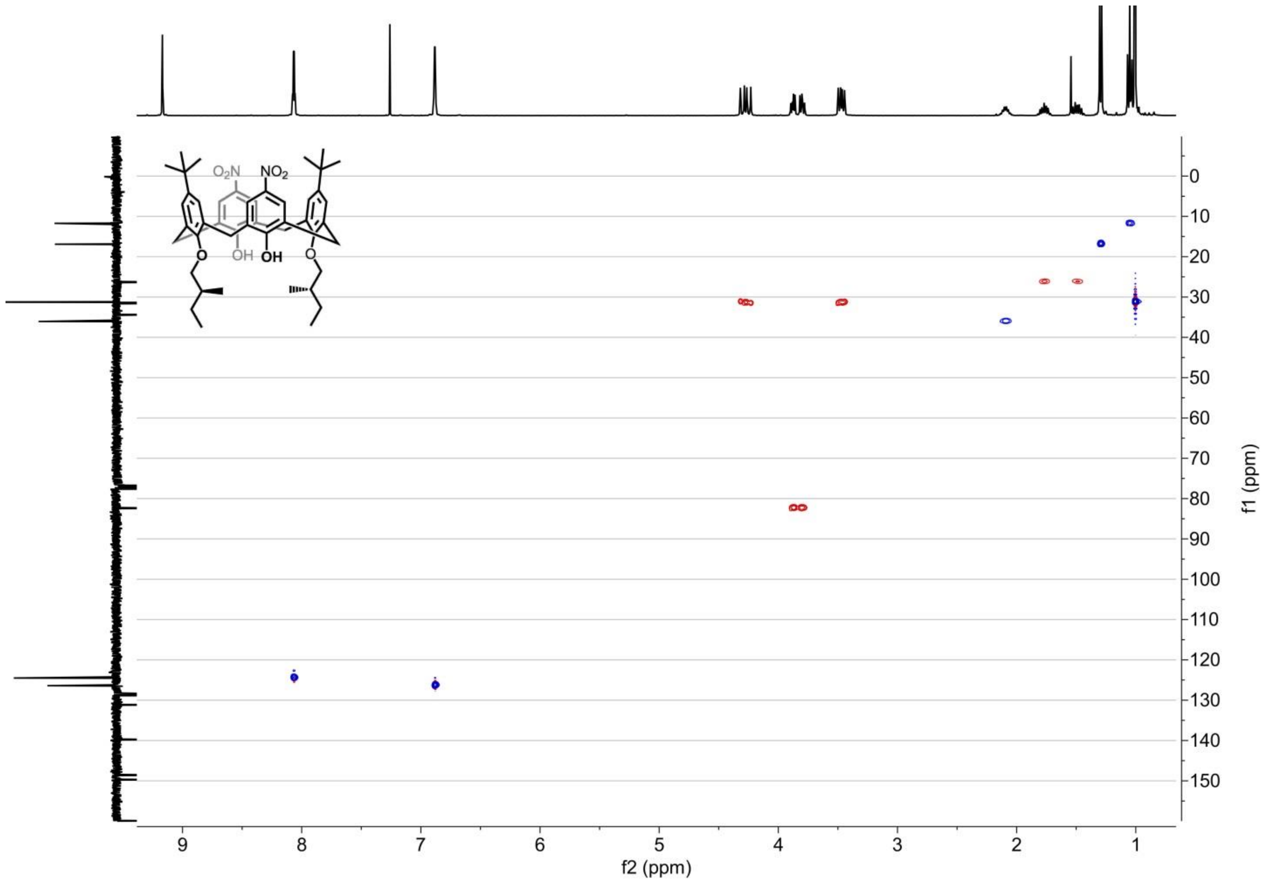

Figure 7. ^{1}H-^{13}C HSQC NMR of compound **3** ($CDCl_3$, 400.1 and 100.6 MHz, 298 K).

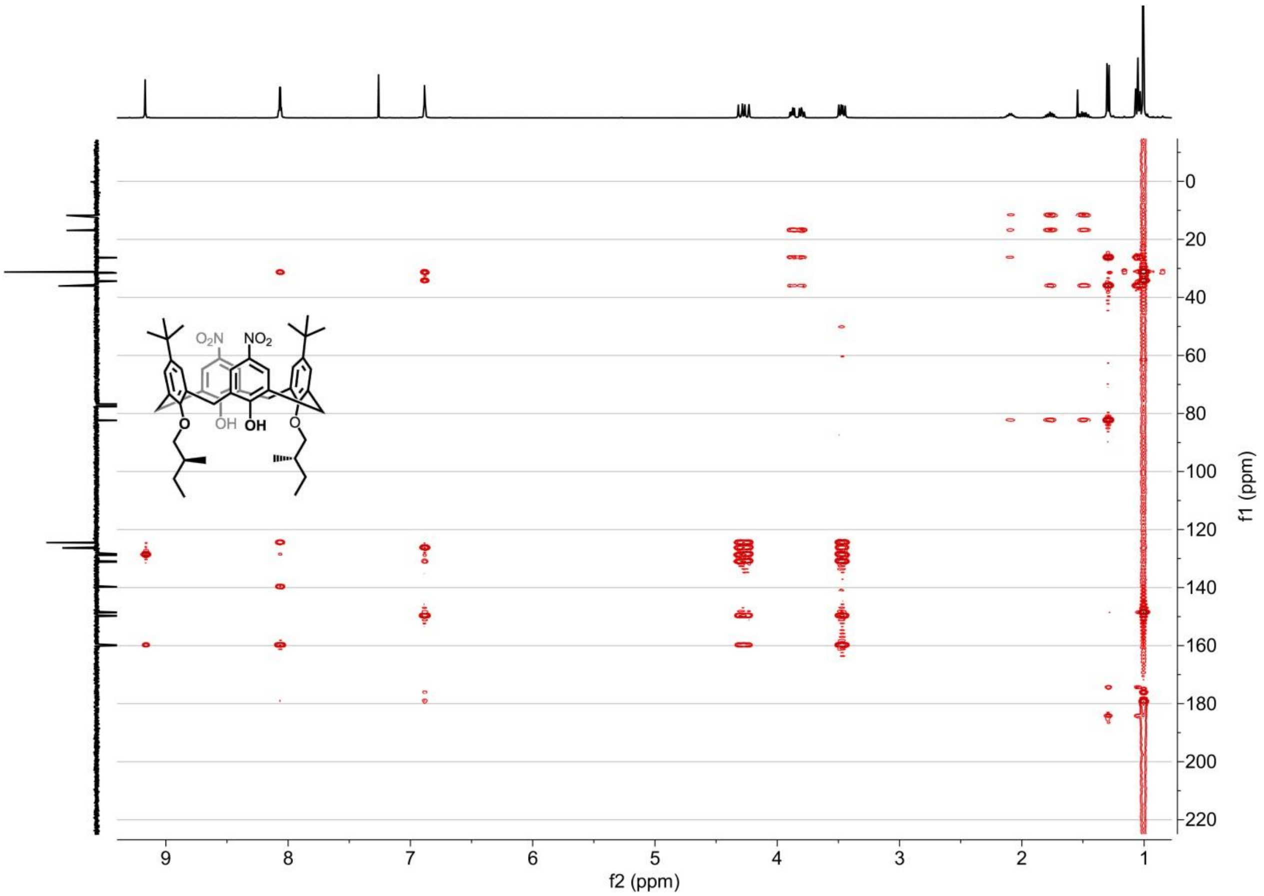

Figure 8. ^{1}H-^{13}C HMBC NMR of compound **3** ($CDCl_3$, 400.1 and 100.6 MHz, 298 K).

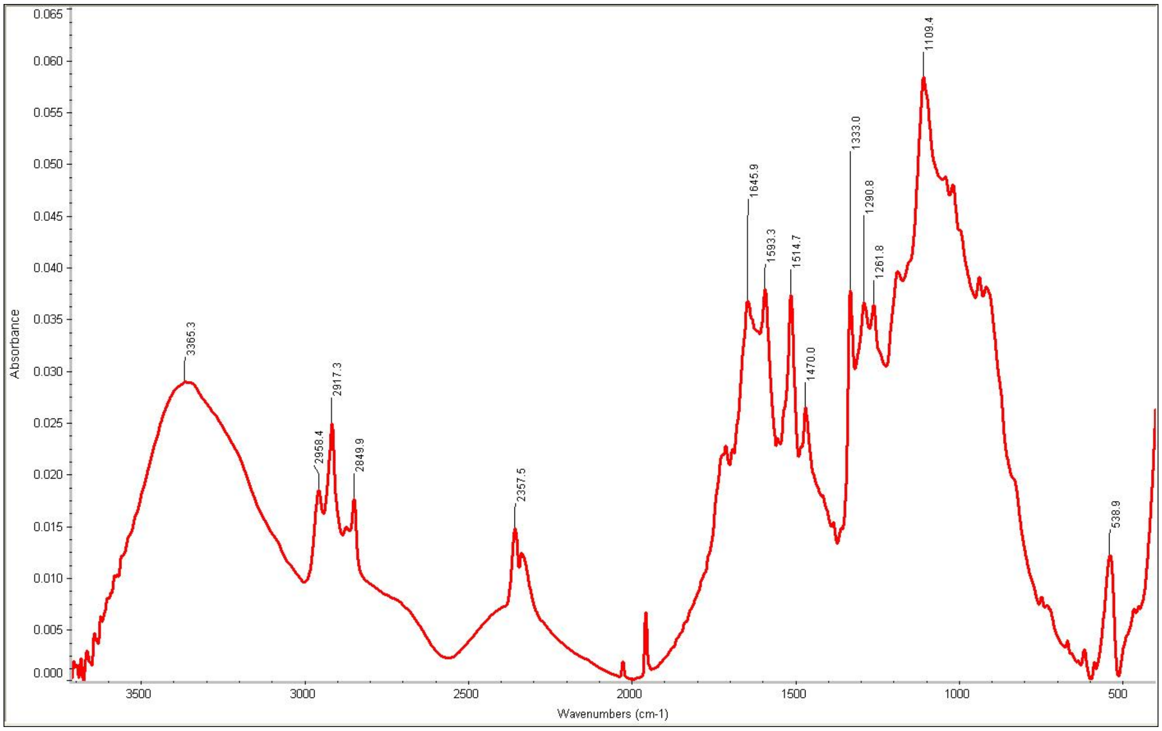

Figure 9. IR of compound **3** (KBr).

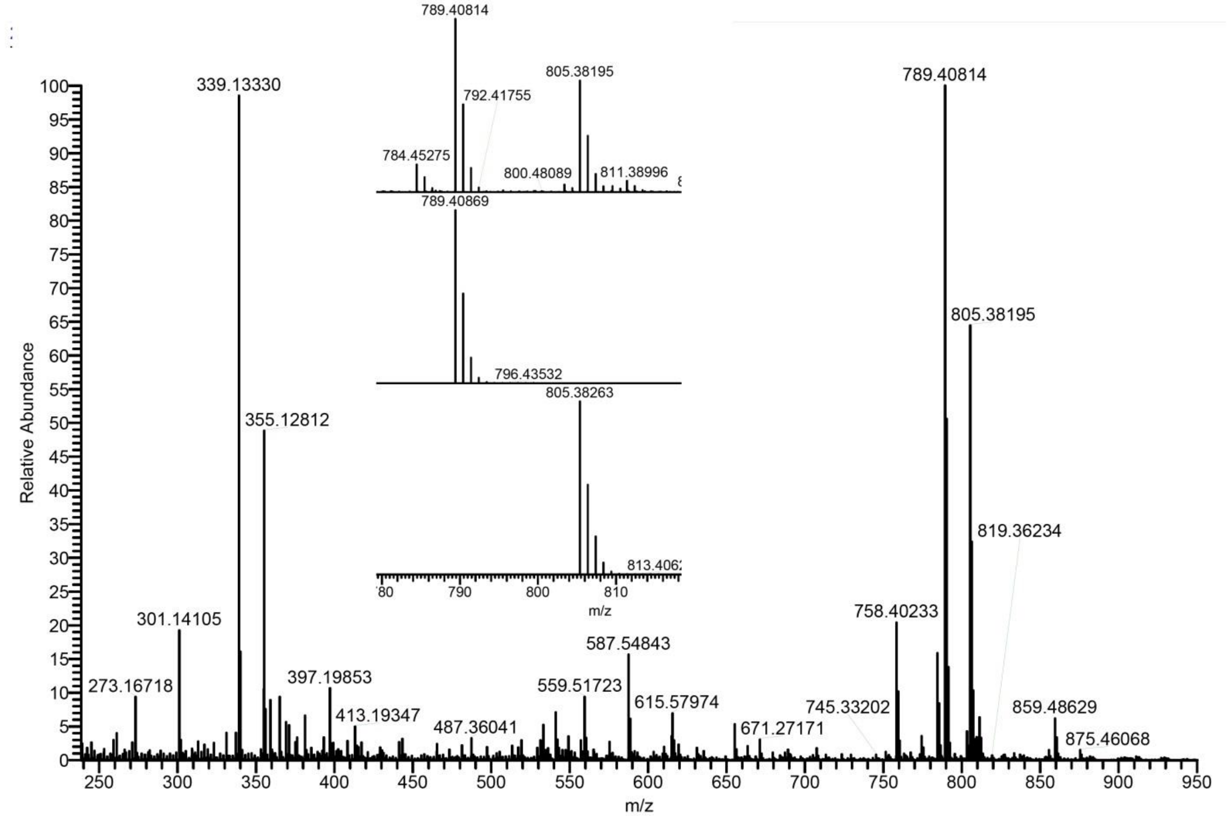

Figure 10. HRMS-ESI of compound **3** (($C_{46}H_{58}N_2O_8$) m/z calcd: 789.4087 $[M+Na]^+$, 805.3826 $[M+K]^+$).

Compound 4a

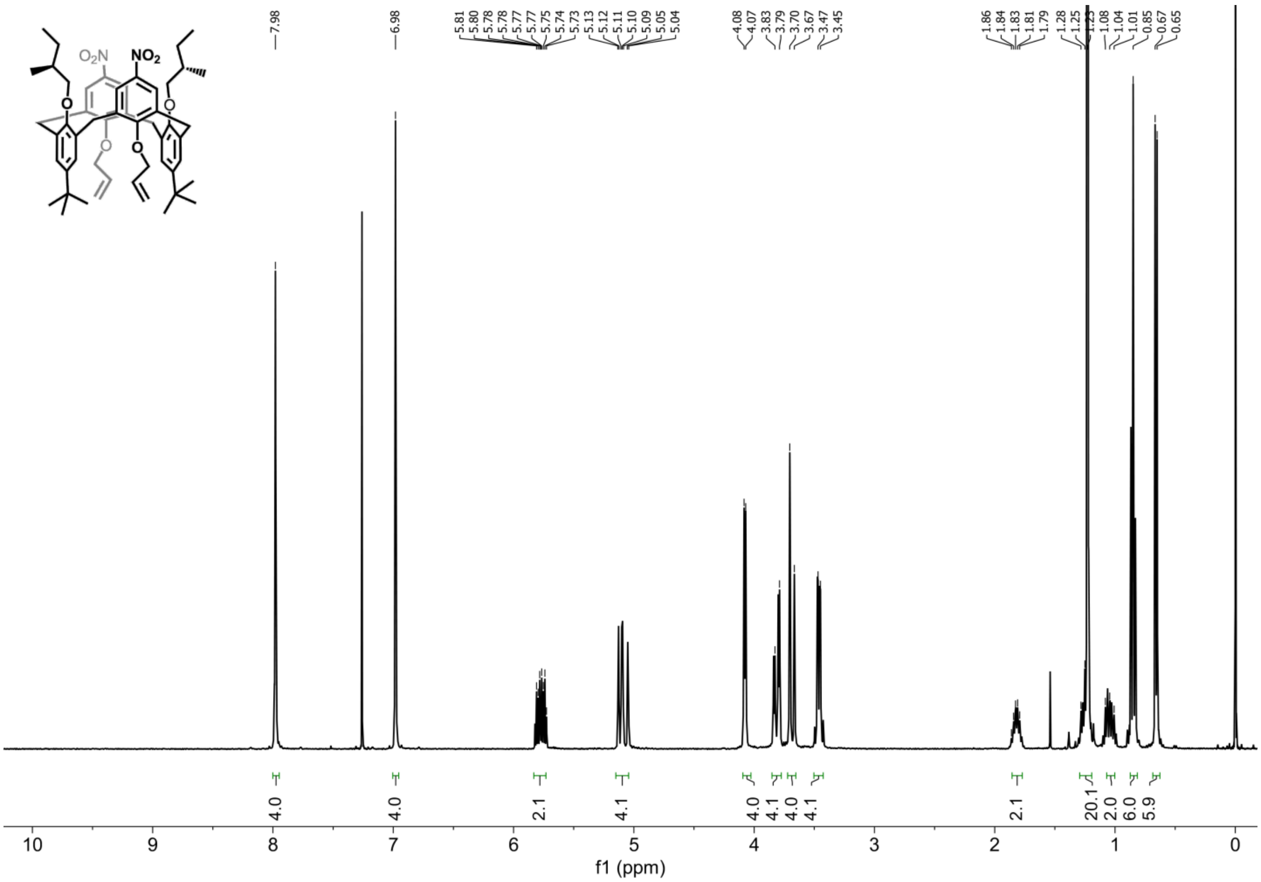

Figure 11. ^1H NMR of compound **4a** ($CDCl_3$, 400.1 MHz, 298 K).

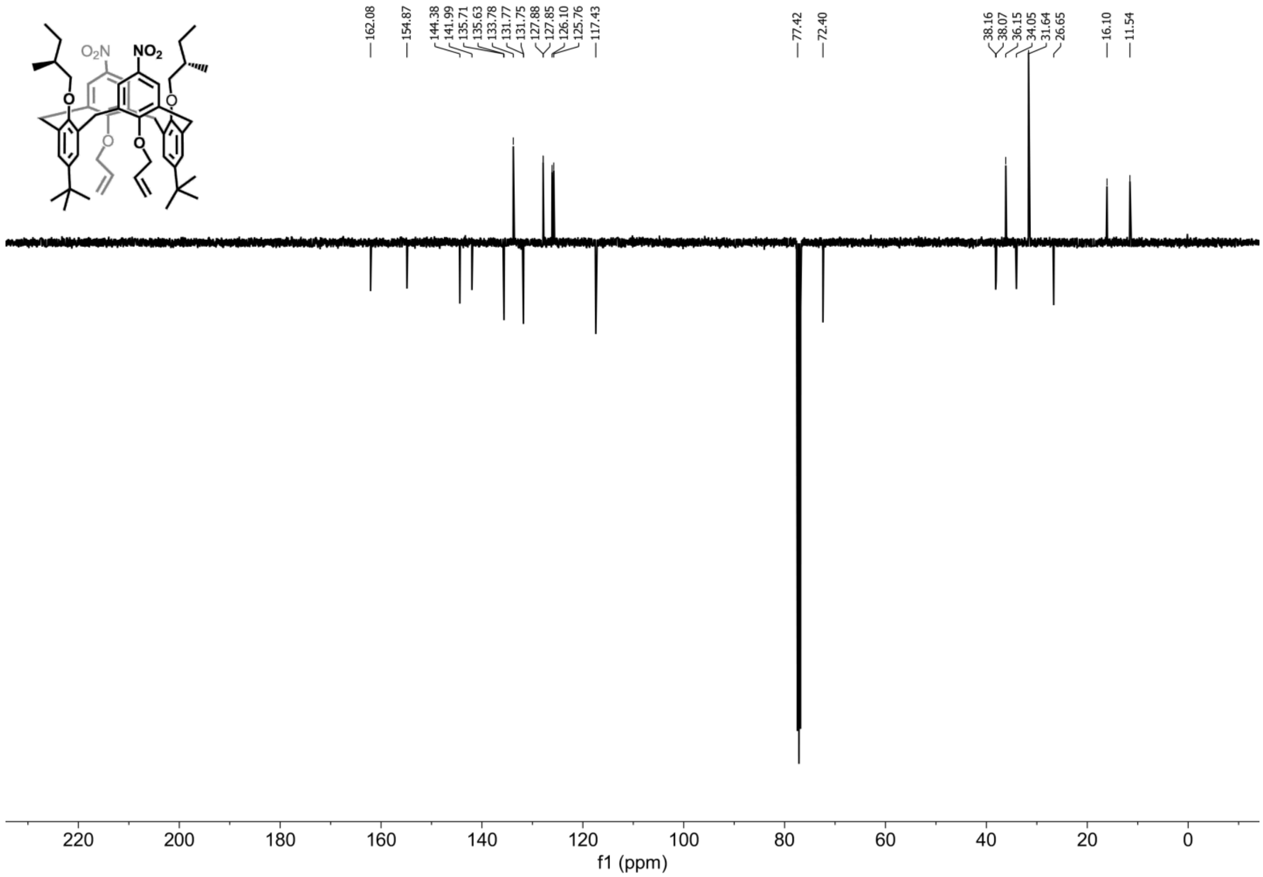

Figure 12. ^{13}C NMR (APT) of compound **4a** ($CDCl_3$, 100.6 MHz, 298 K).

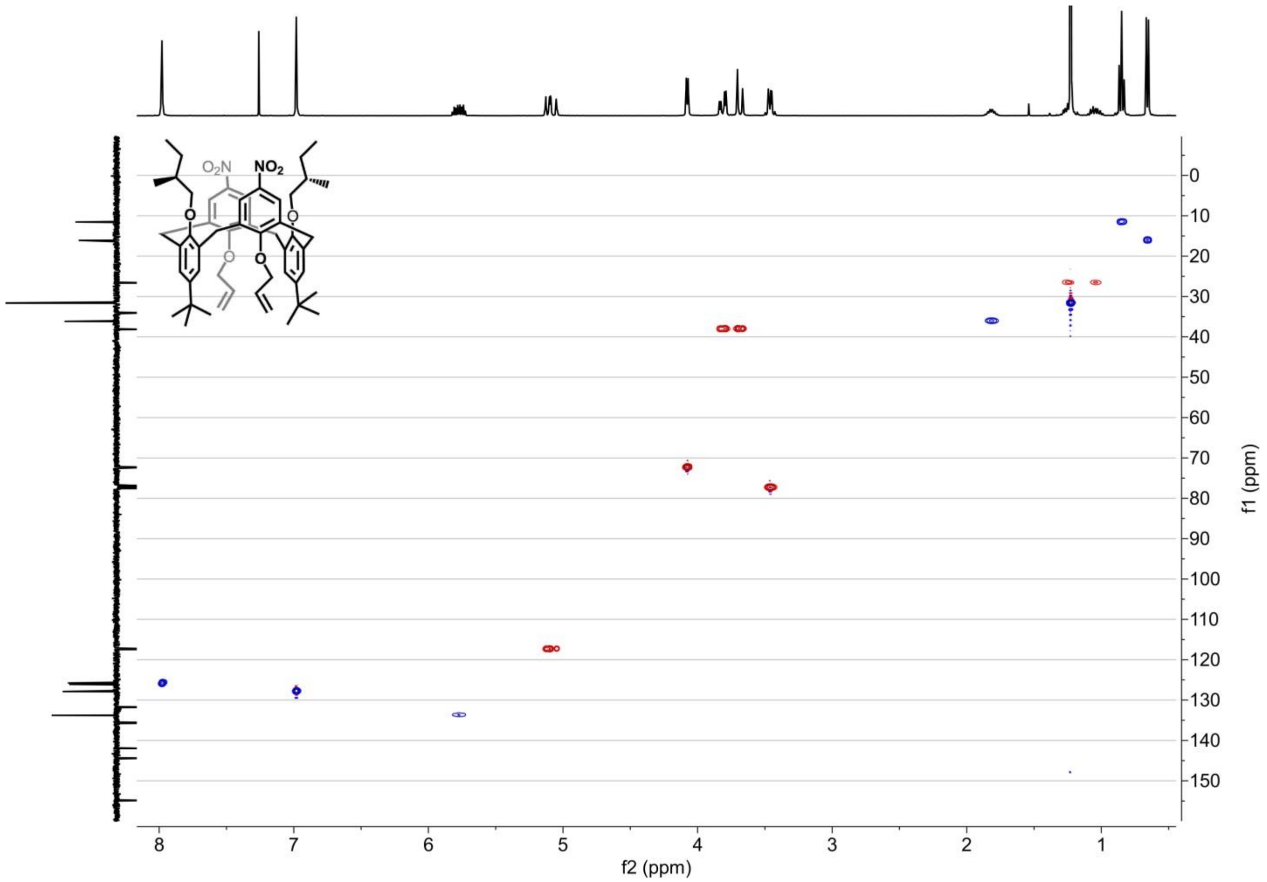

Figure 13. ^{1}H-^{13}C HSQC NMR of compound **4a** ($CDCl_3$, 400.1 and 100.6 MHz, 298 K).

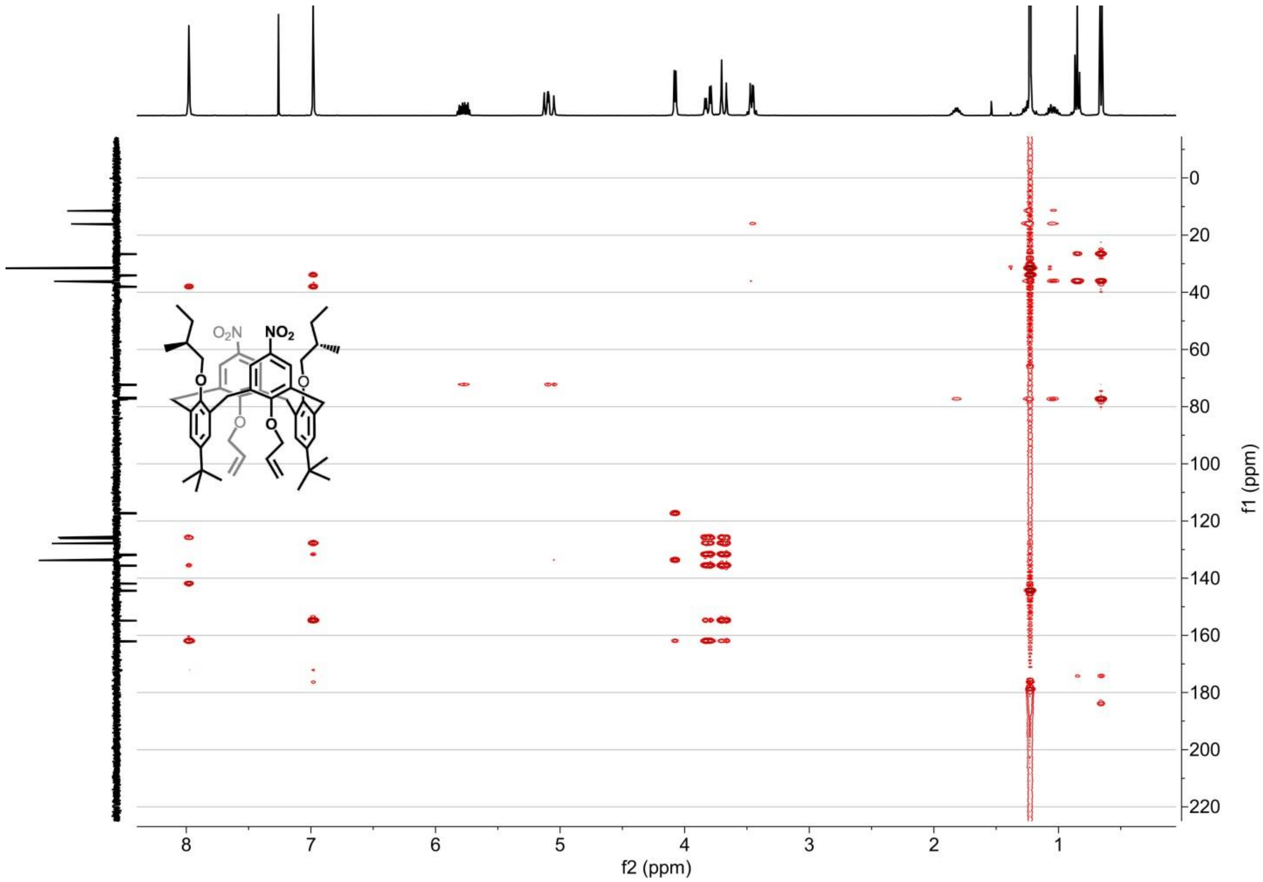

Figure 14. ^{1}H-^{13}C HMBC NMR of compound **4a** ($CDCl_3$, 400.1 and 100.6 MHz, 298 K).

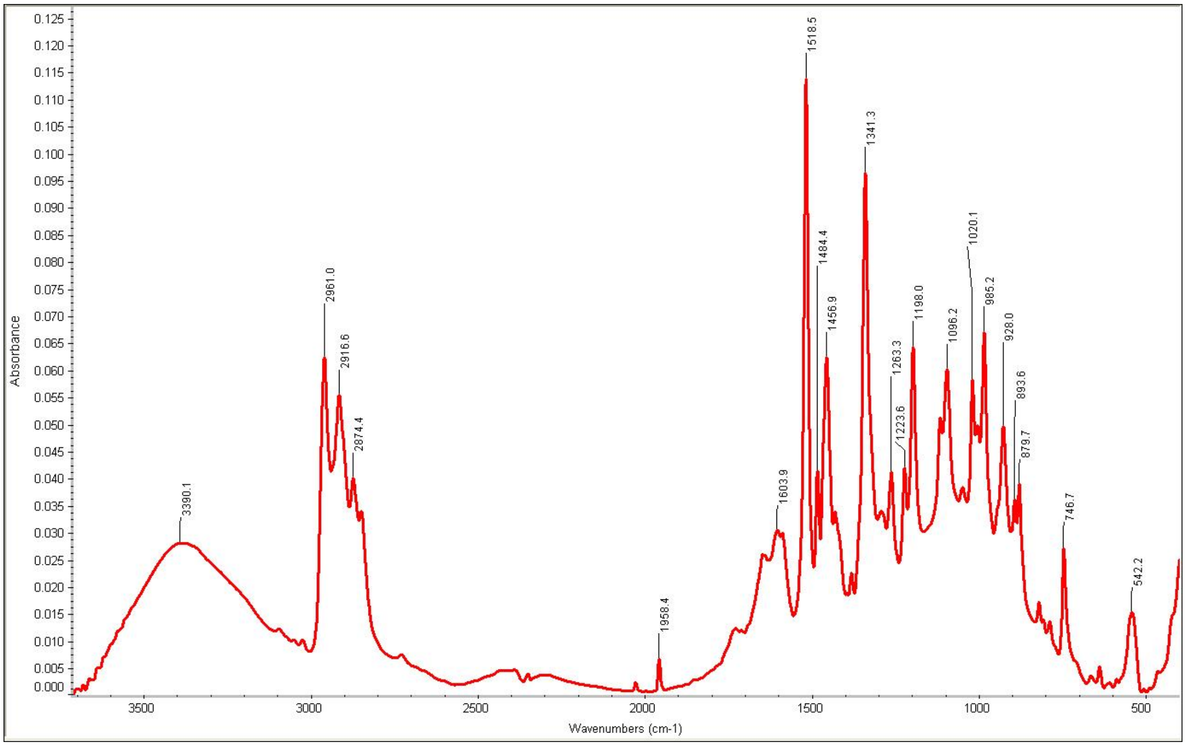

Figure 15. IR of compound **4a** (KBr).

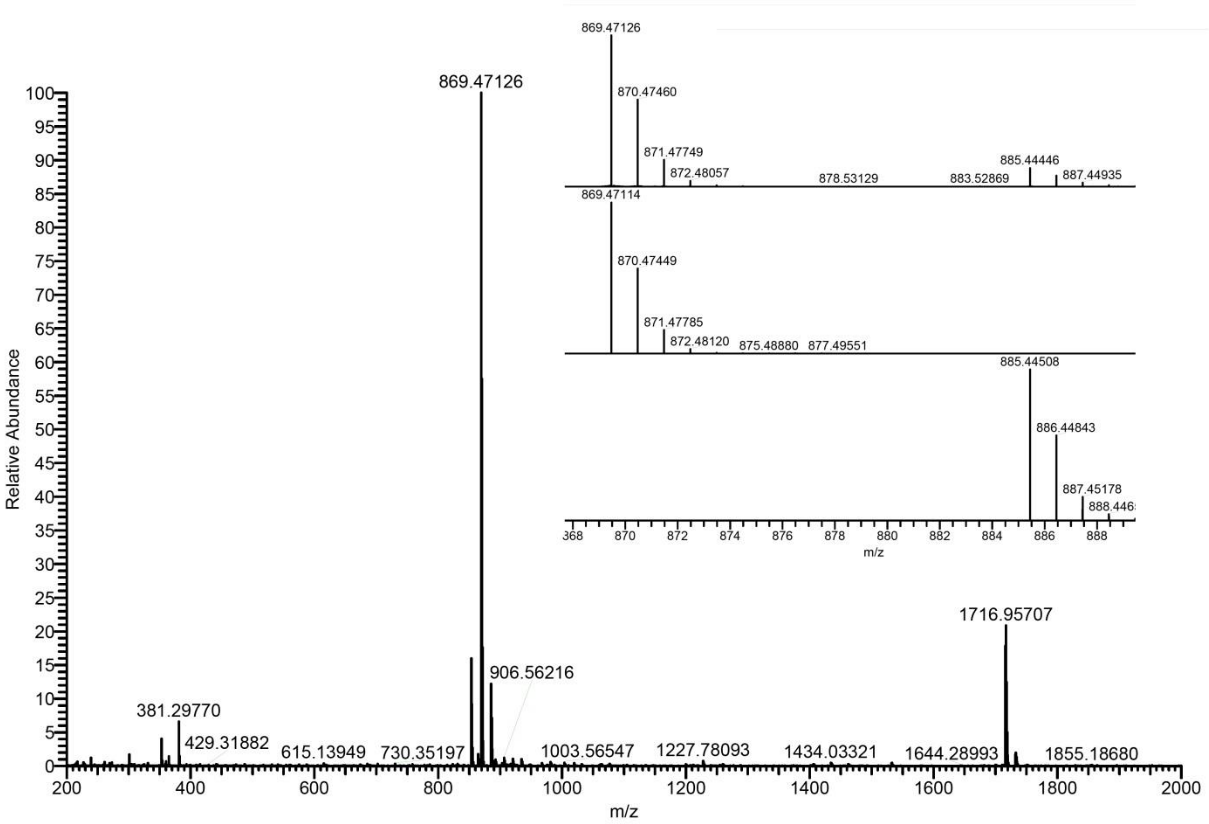

Figure 16. HRMS-ESI of compound **4a** (($C_{52}H_{66}N_2O_8$) m/z calcd: 869.4711 $[M+Na]^+$, 885.4451 $[M+K]^+$).

Compound 4b

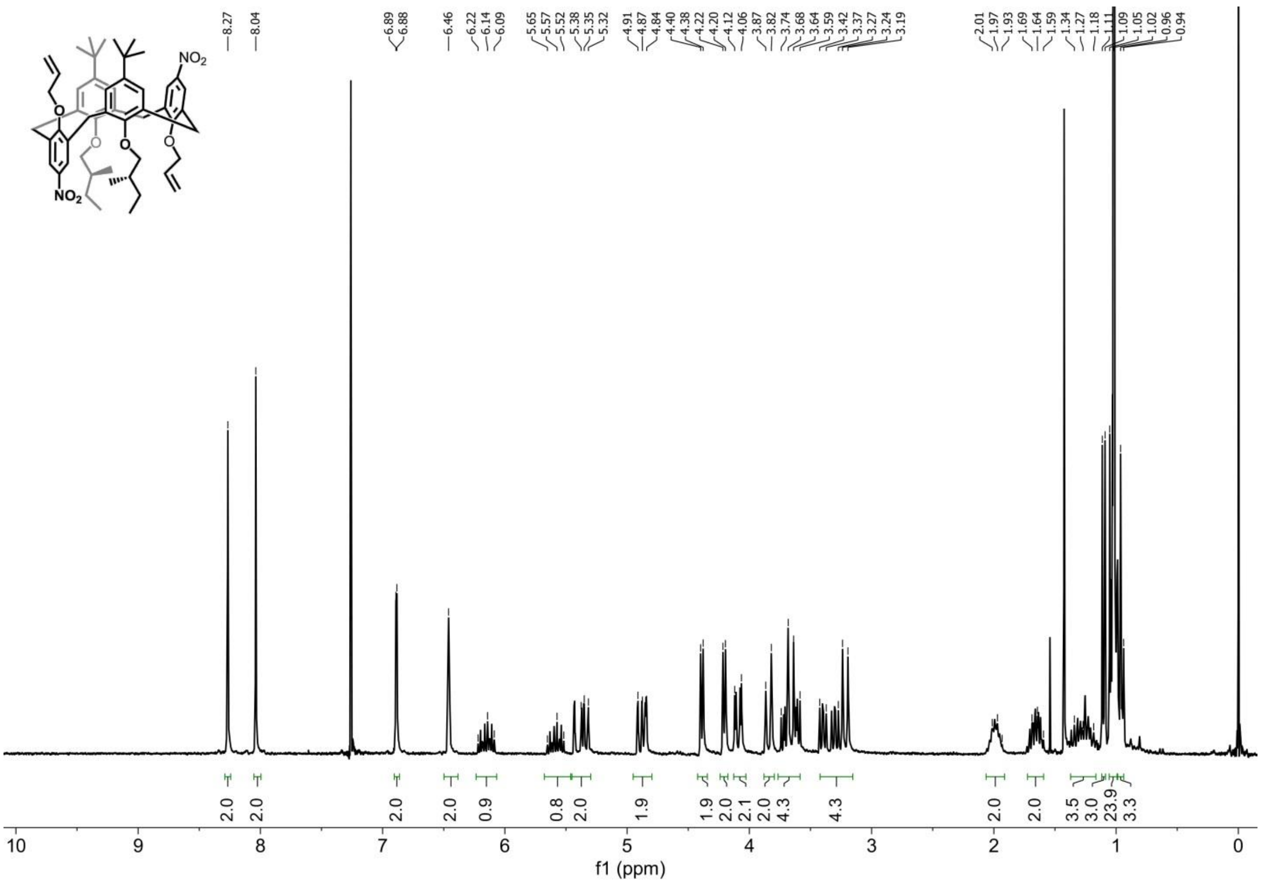

Figure 17. ^1H NMR of compound **4b** ($CDCl_3$, 400.1 MHz, 298 K).

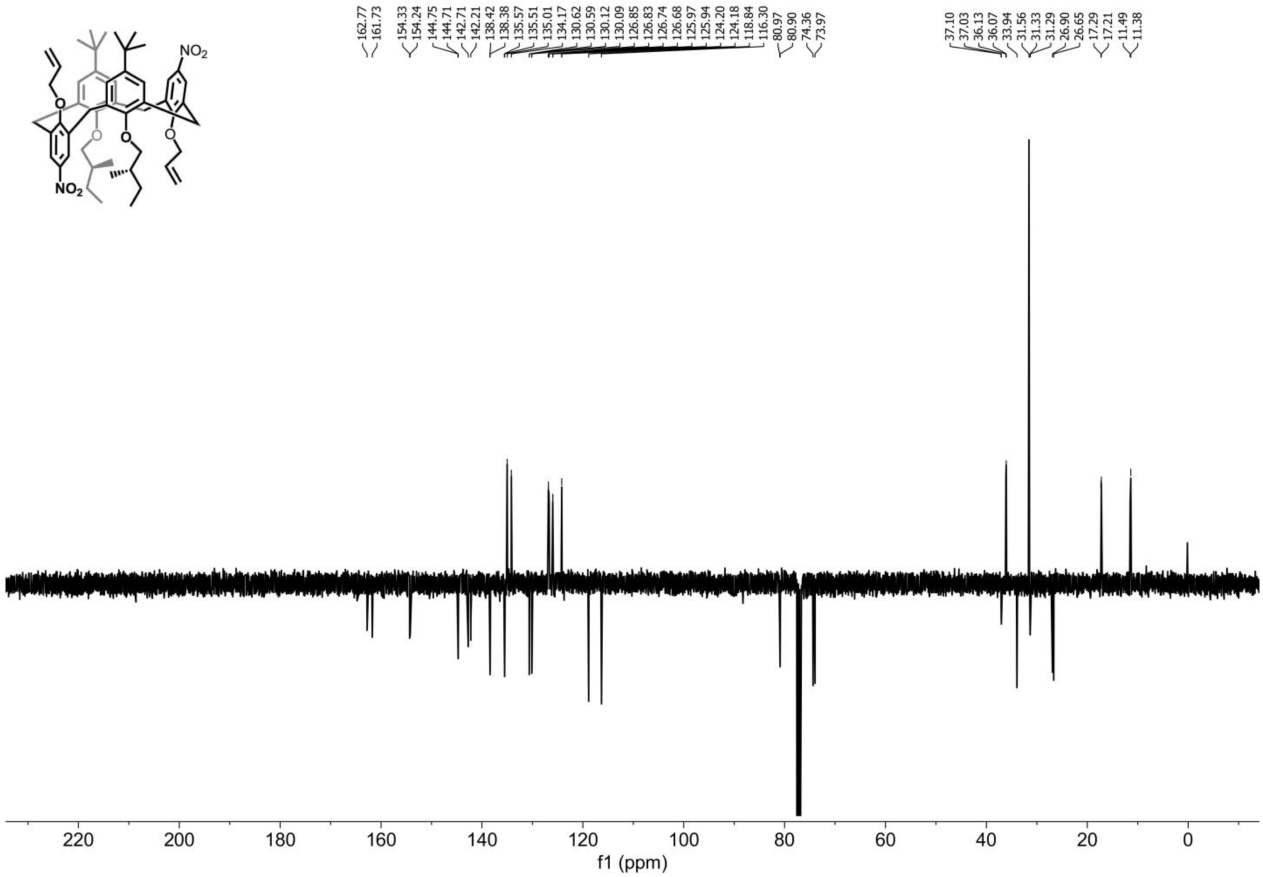

Figure 18. ^{13}C NMR (APT) of compound **4b** ($CDCl_3$, 100.6 MHz, 298 K).

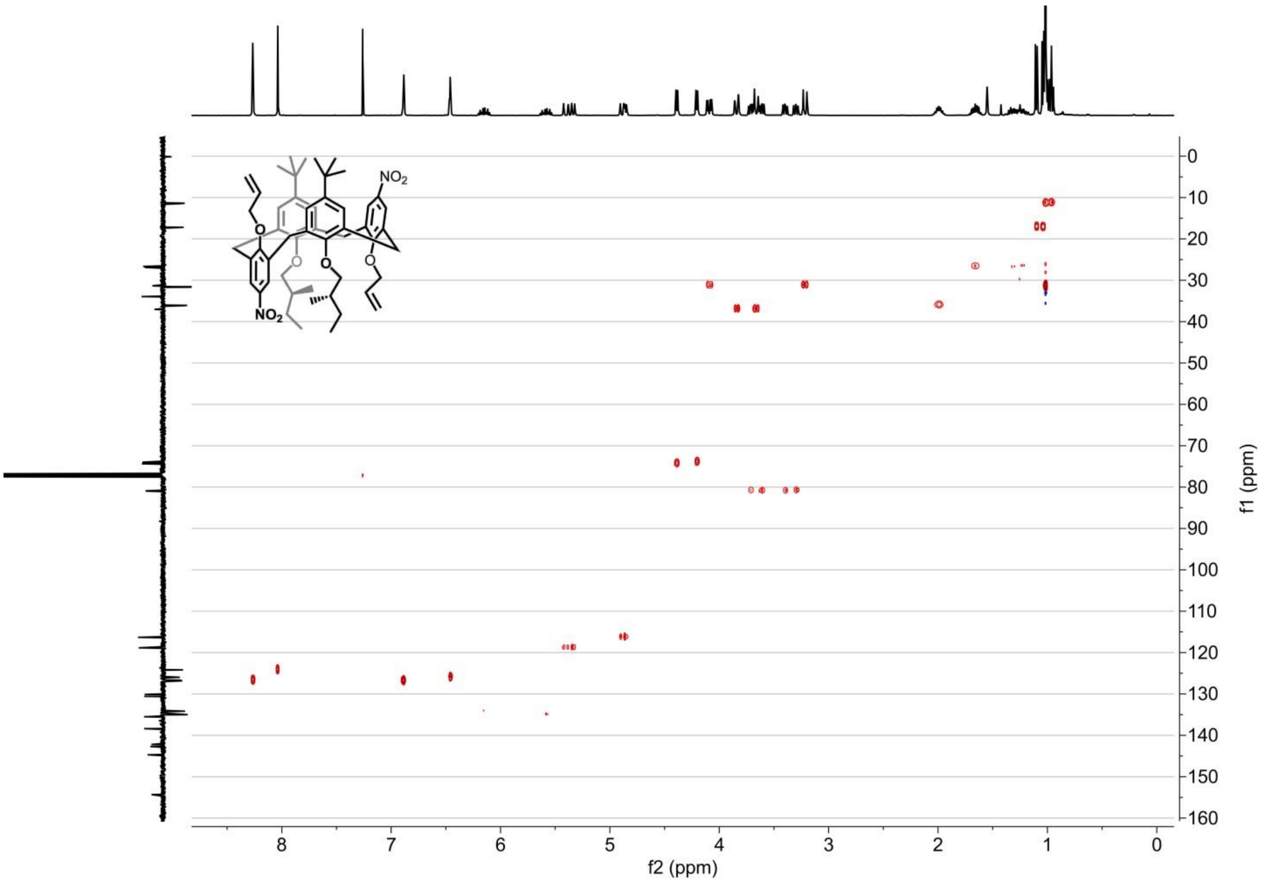

Figure 19. ^{1}H-^{13}C HSQC NMR of compound **4b** ($CDCl_3$, 400.1 and 100.6 MHz, 298 K).

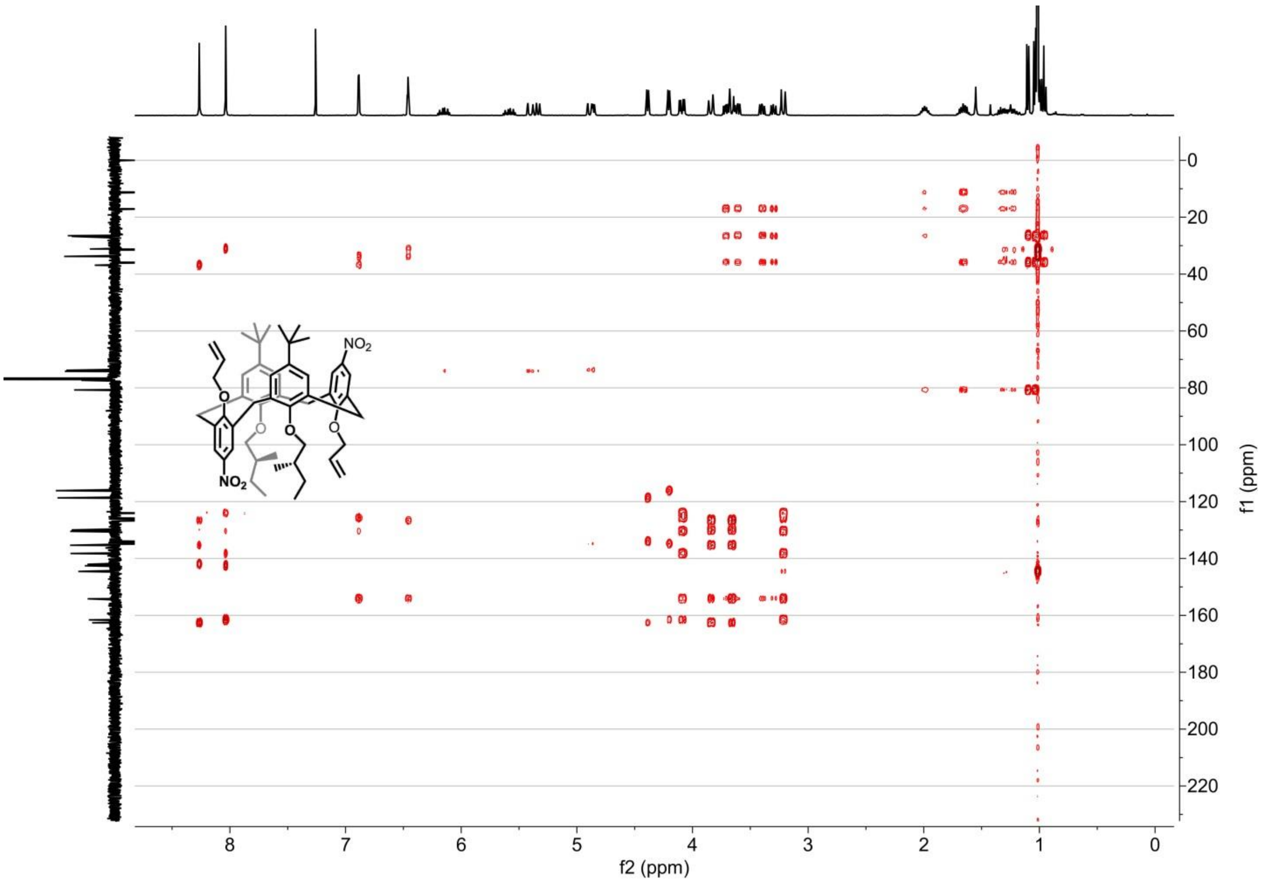

Figure 20. ^{1}H-^{13}C HMBC NMR of compound **4b** ($CDCl_3$, 400.1 and 100.6 MHz, 298 K).

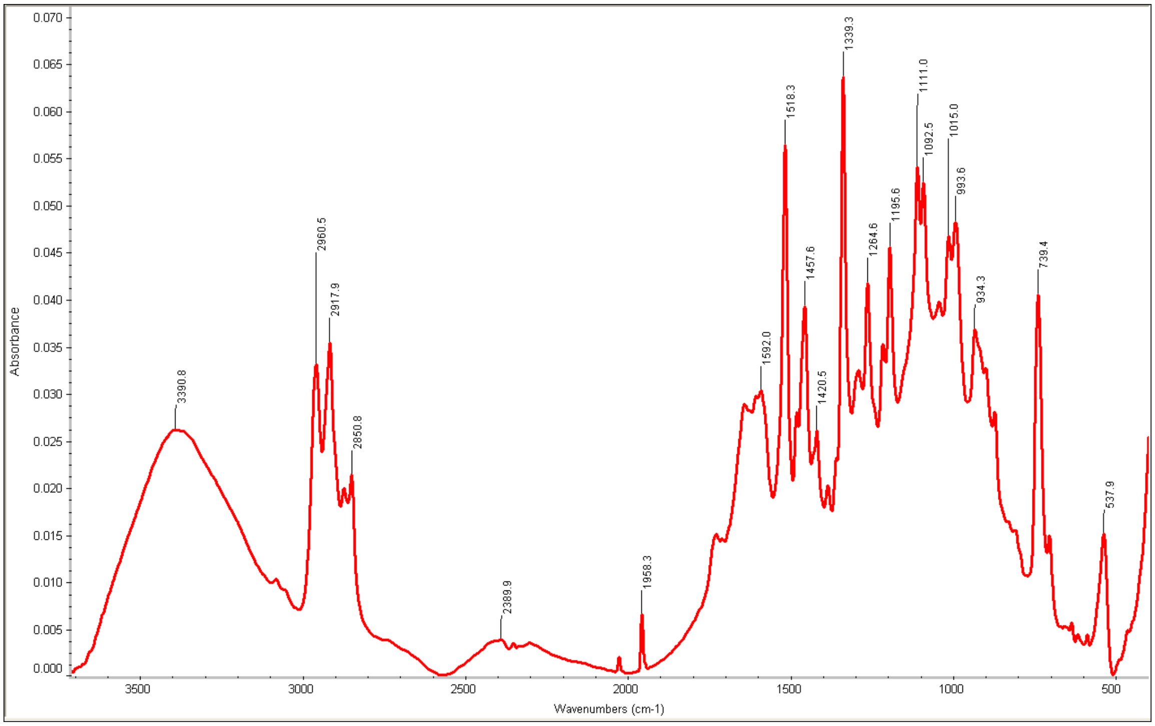

Figure 21. IR of compound **4b** (KBr).

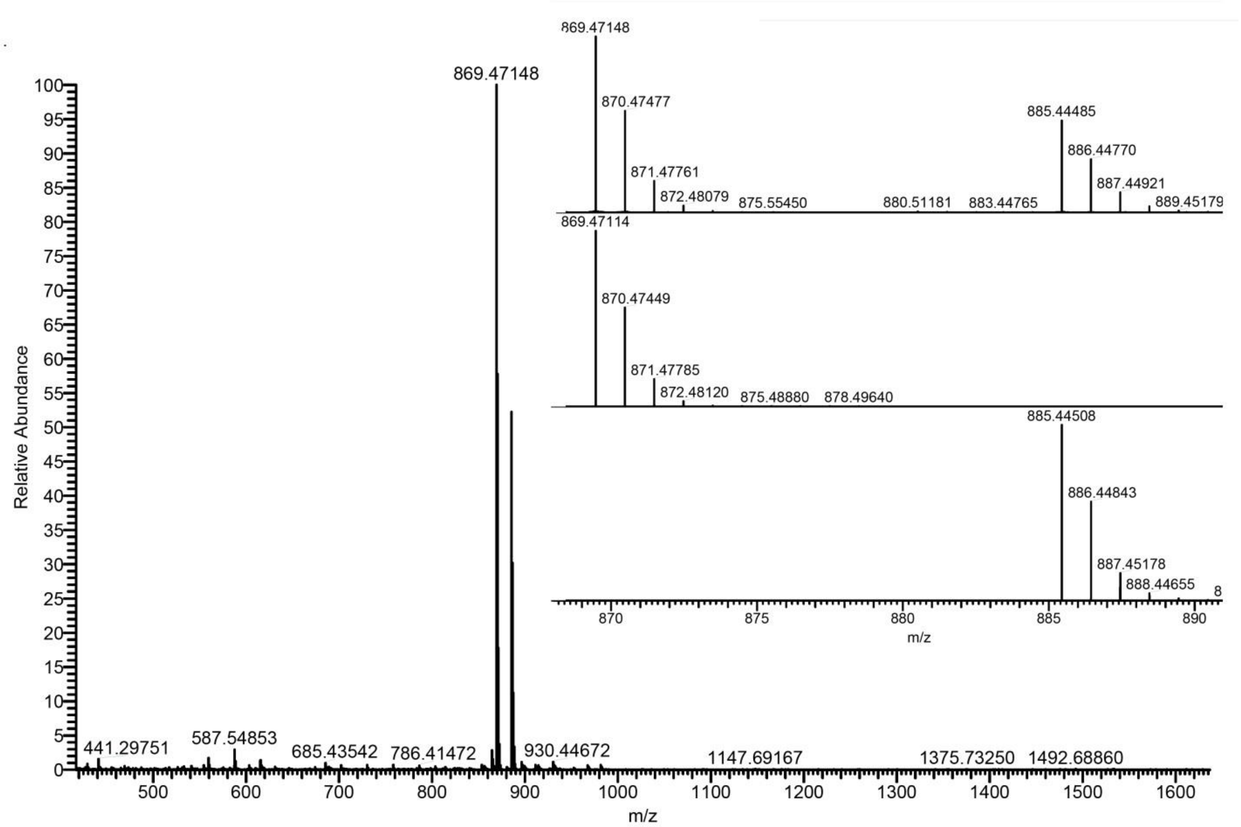

Figure 22. HRMS-ESI of compound **4b** (($C_{52}H_{66}N_2O_8$) m/z calcd: 869.4711 $[M+Na]^+$, 885.4451$[M+K]^+$).

Compound 5

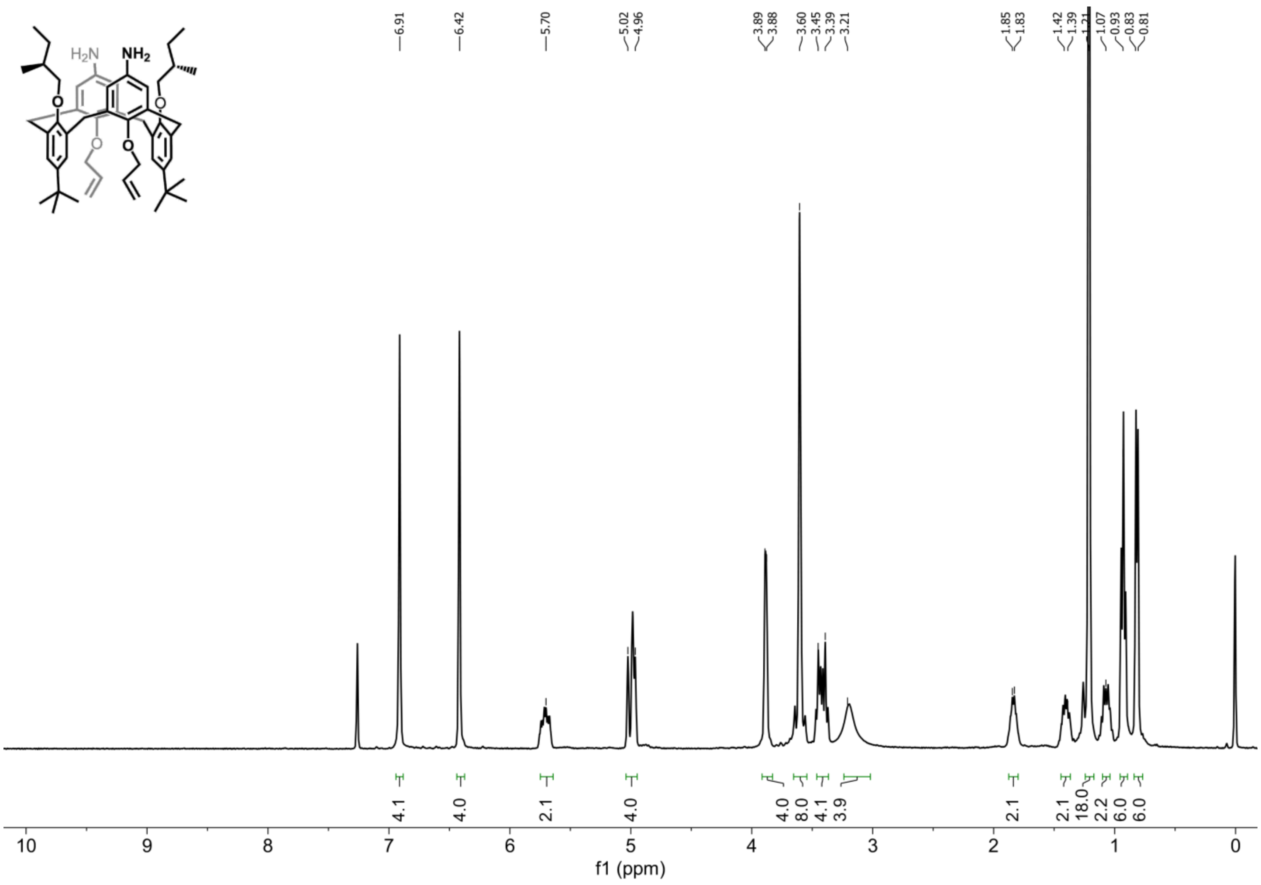

Figure 23. ^1H NMR of compound **5** ($CDCl_3$, 400.1 MHz, 298 K).

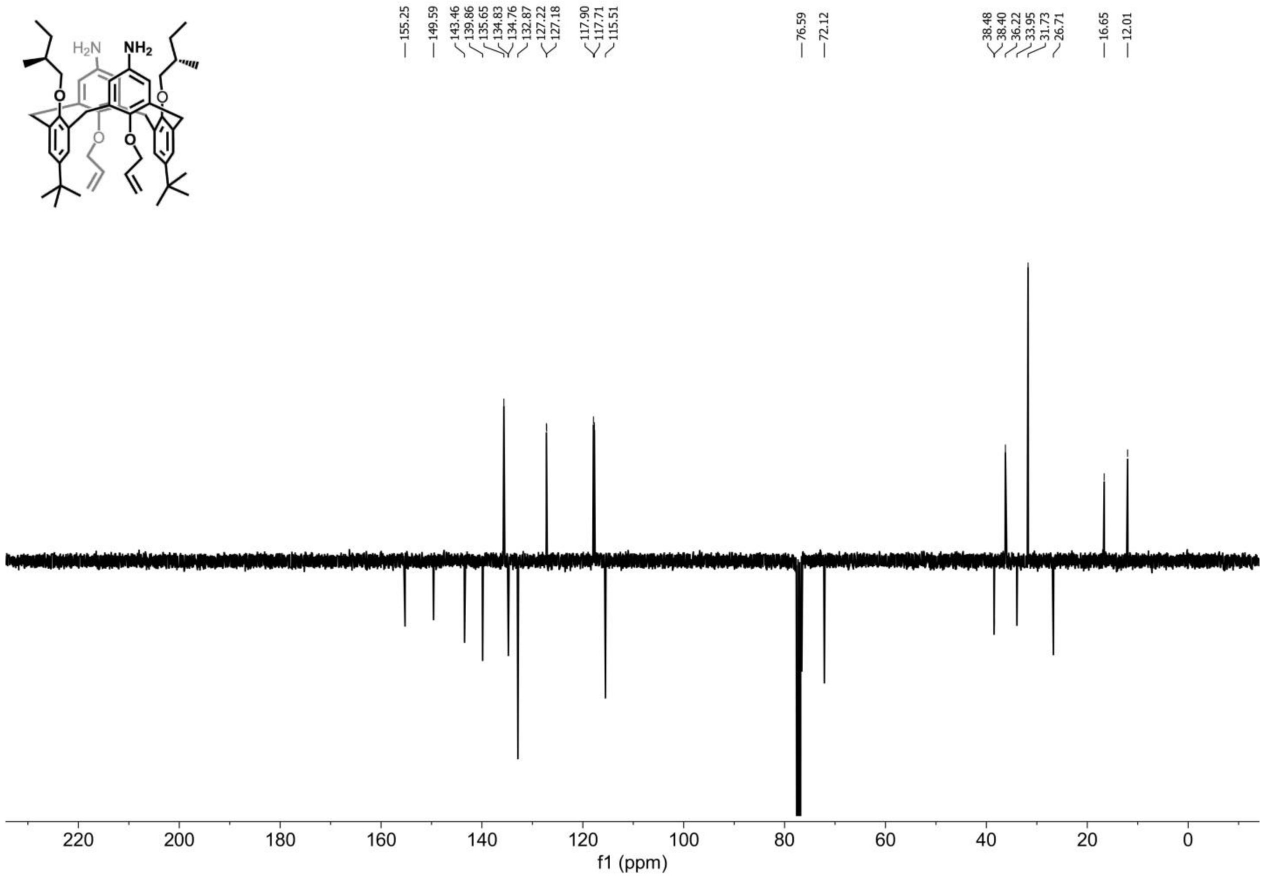

Figure 24. ^{13}C NMR (APT) of compound **5** ($CDCl_3$, 100.6 MHz, 298 K).

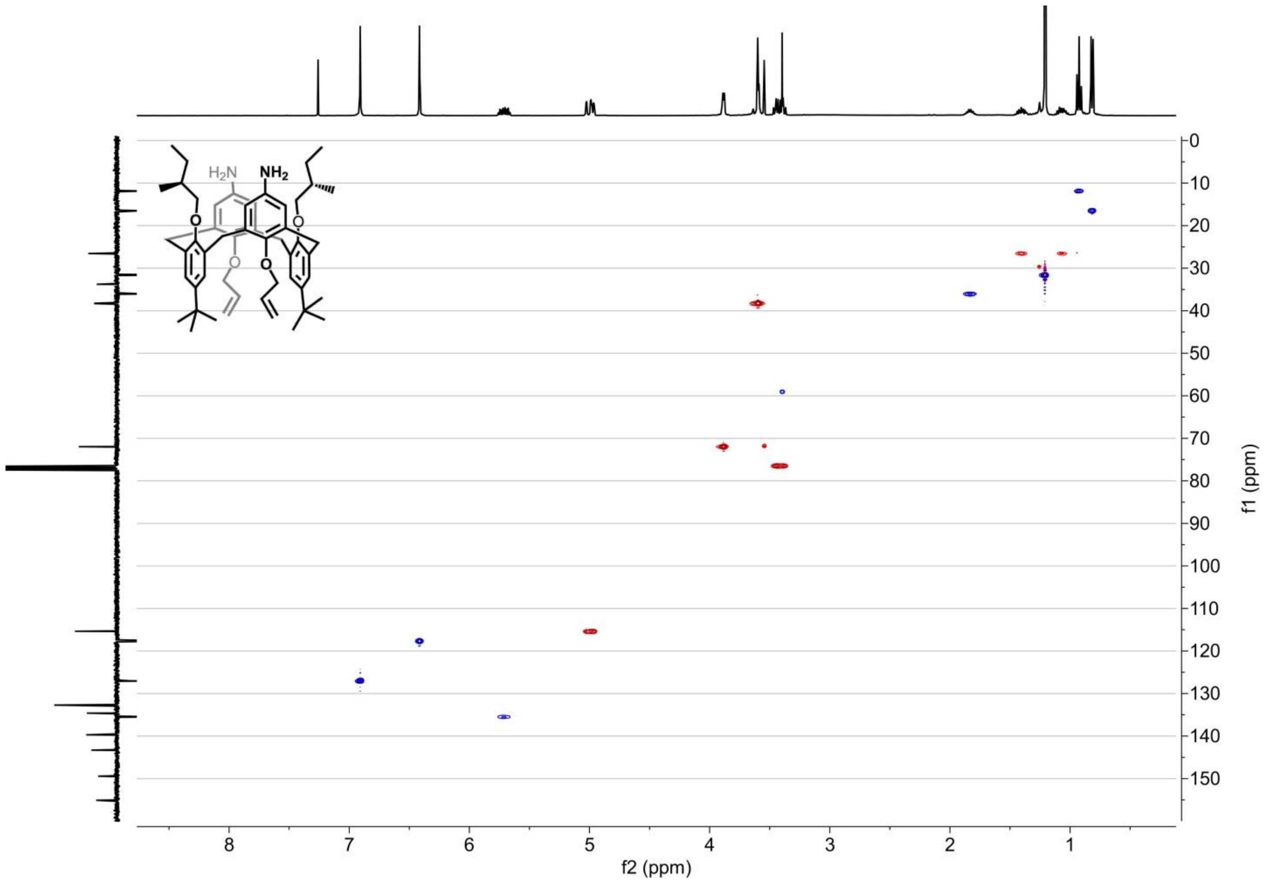

Figure 25. ^{1}H-^{13}C HSQC NMR of compound **5** ($CDCl_3$, 400.1 and 100.6 MHz, 298 K).

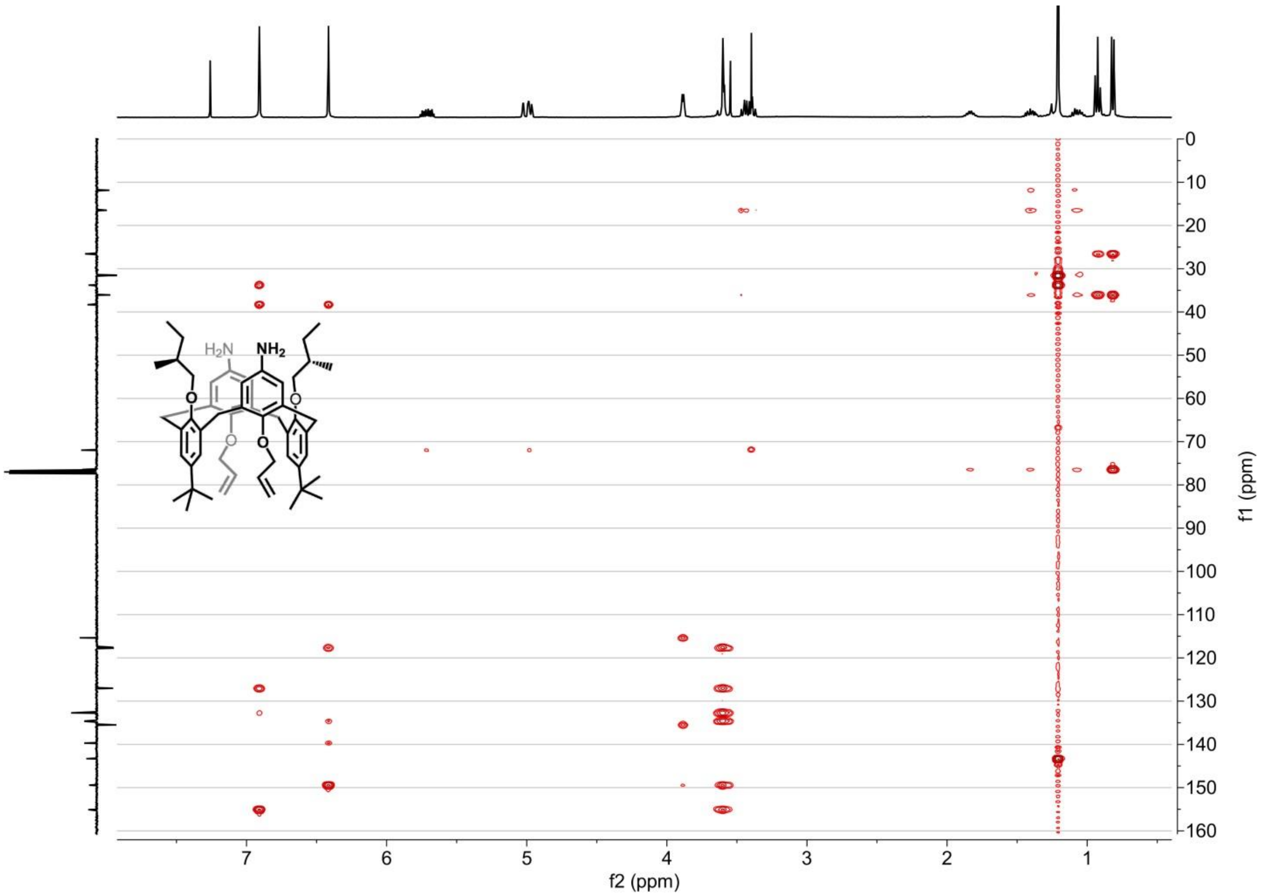

Figure 26. ^{1}H-^{13}C HMBC NMR of compound **5** ($CDCl_3$, 400.1 and 100.6 MHz, 298 K).

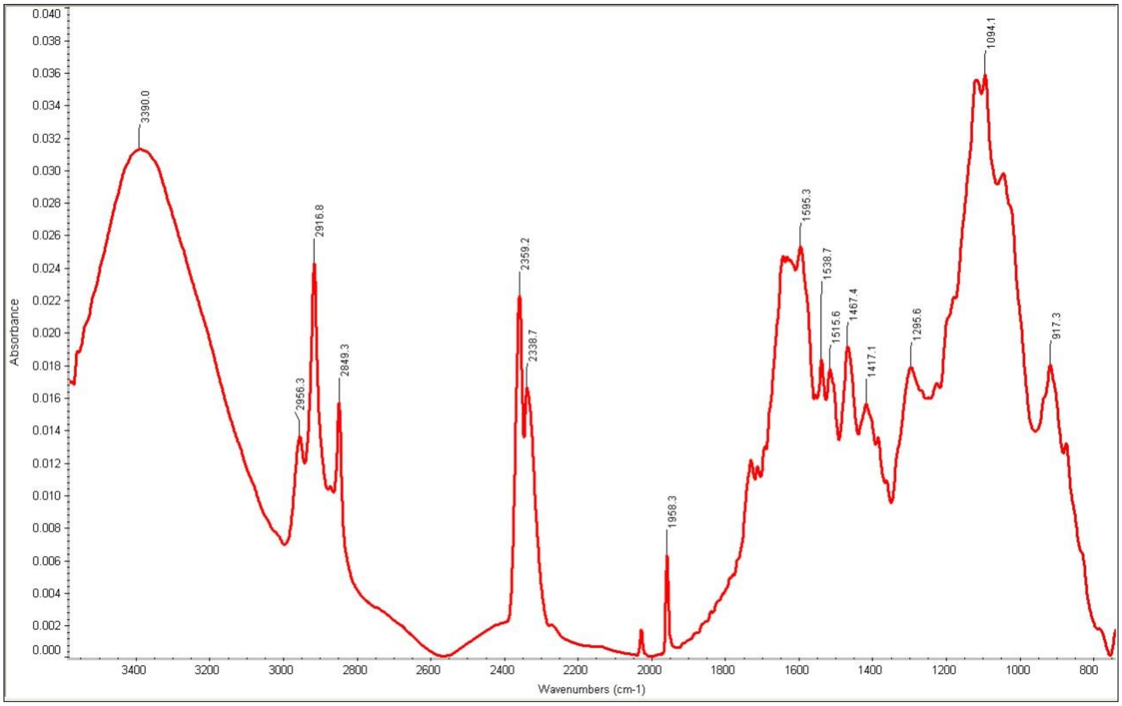

Figure 27. IR of compound **5** (KBr).

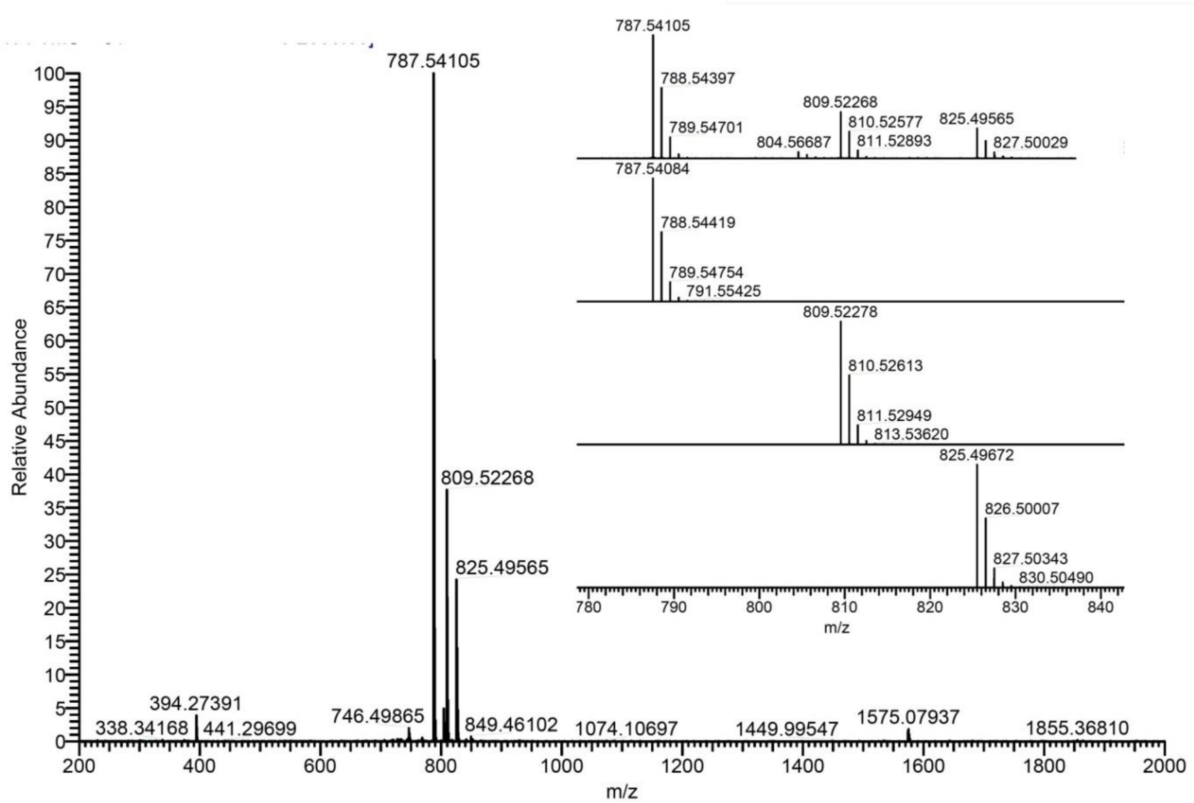

Figure 28. HRMS-ESI of compound **5** (($C_{52}H_{70}N_2O_4$) m/z calcd: 787.5408 $[M+H]^+$, 809.5228 $[M+Na]^+$, 825.4967 $[M+K]^+$).

Compound 6

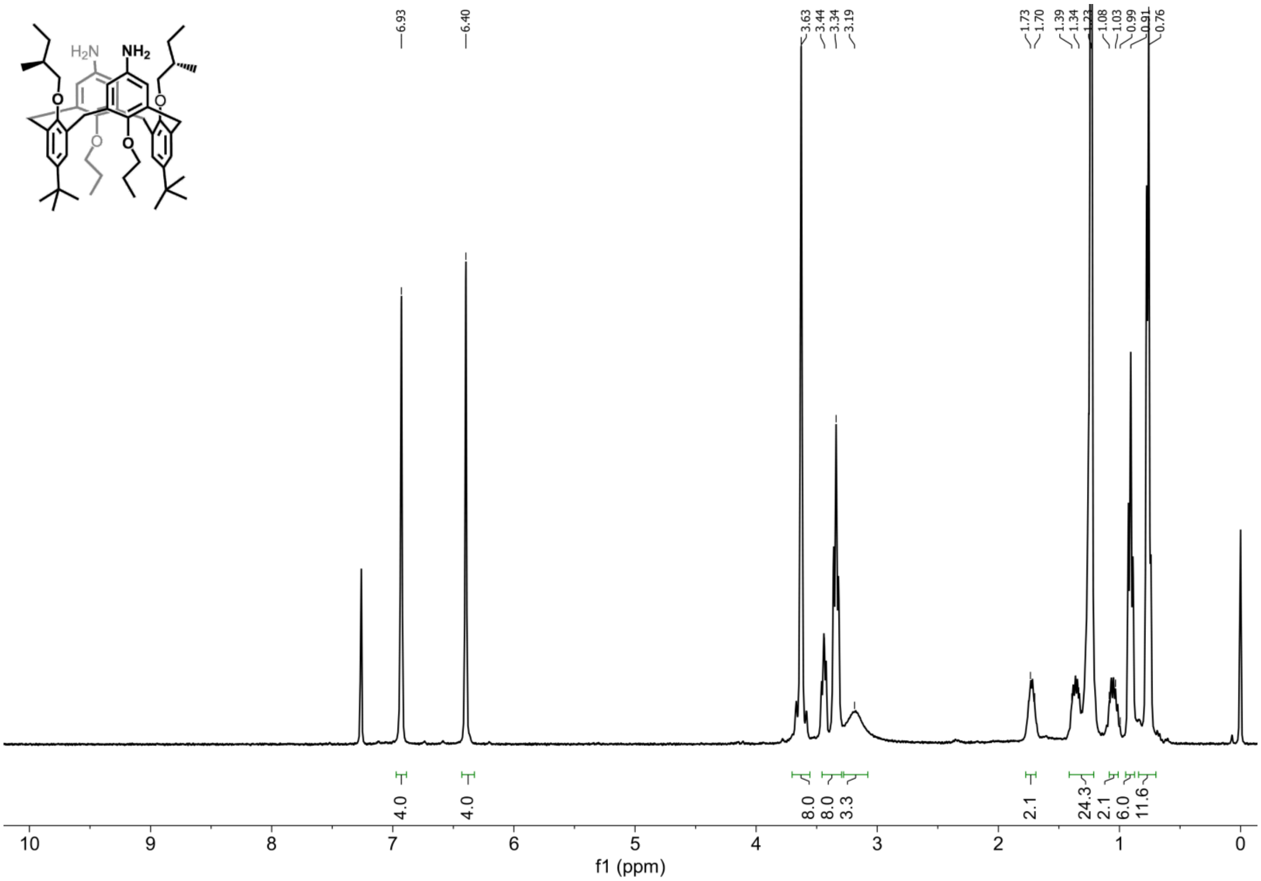

Figure 29. ^{1}H NMR of compound **6** ($CDCl_3$, 400.1 MHz, 298 K).

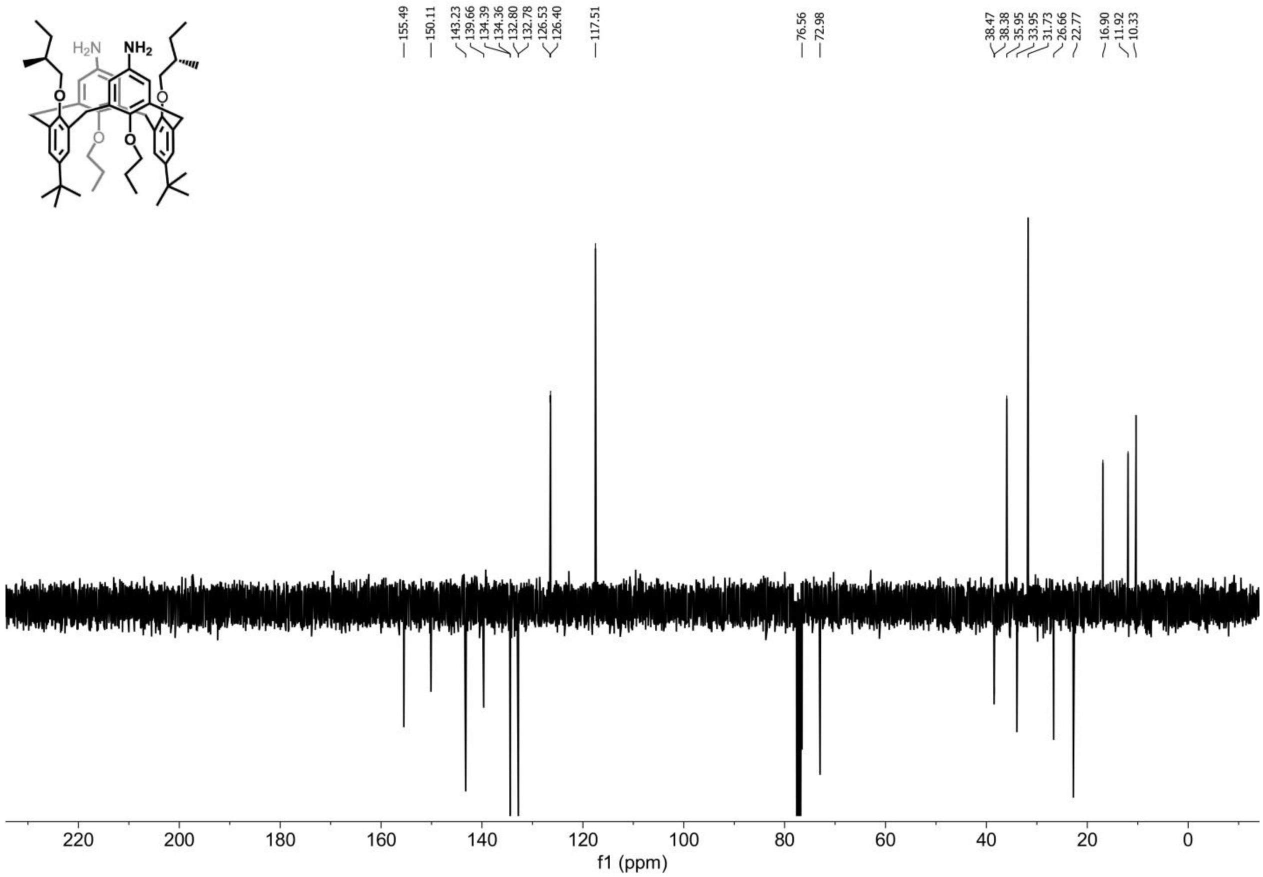

Figure 30. ^{13}C NMR (APT) of compound **6** ($CDCl_3$, 100.6 MHz, 298 K).

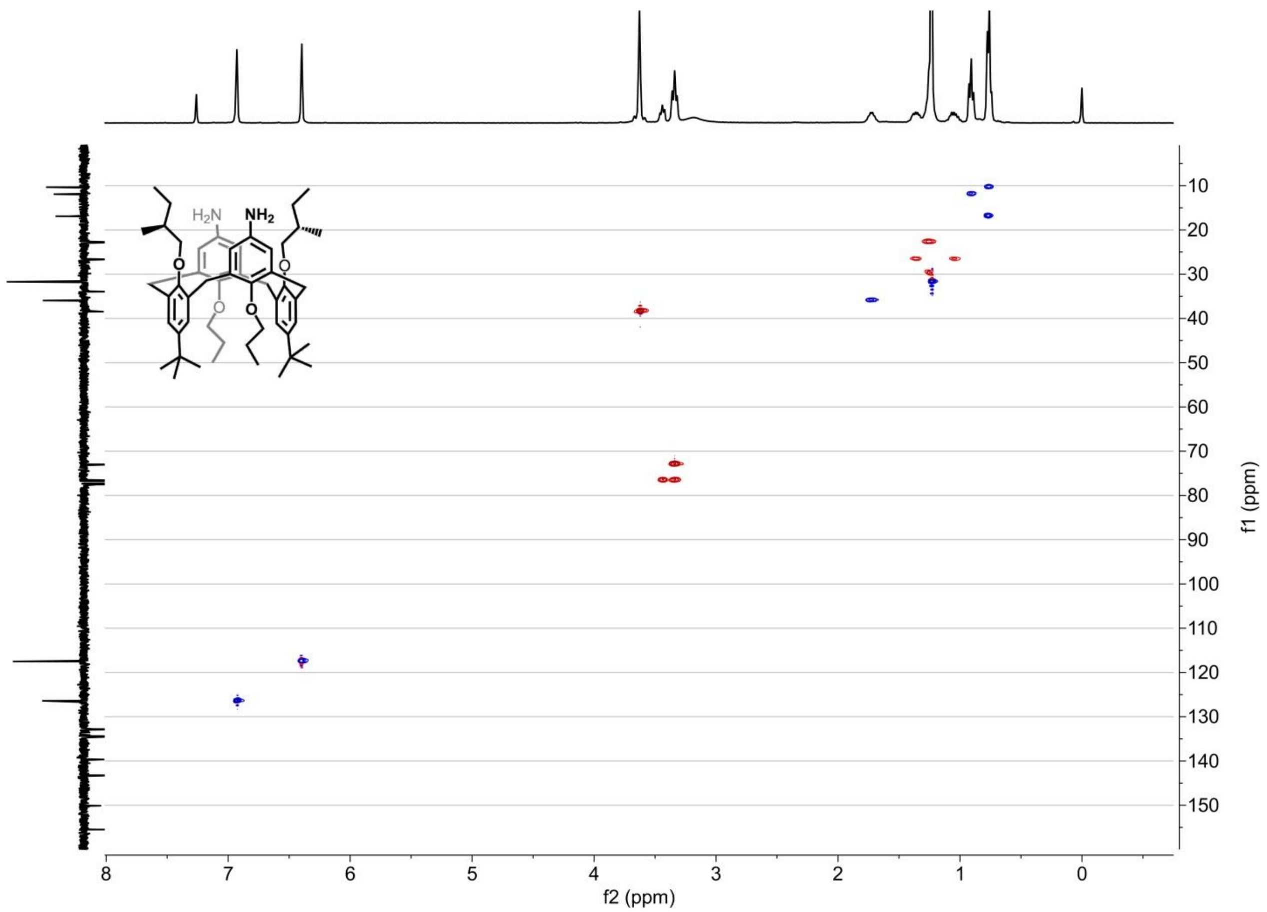

Figure 31. ^{1}H-^{13}C HSQC NMR of compound **6** ($CDCl_3$, 400.1 and 100.6 MHz, 298 K).

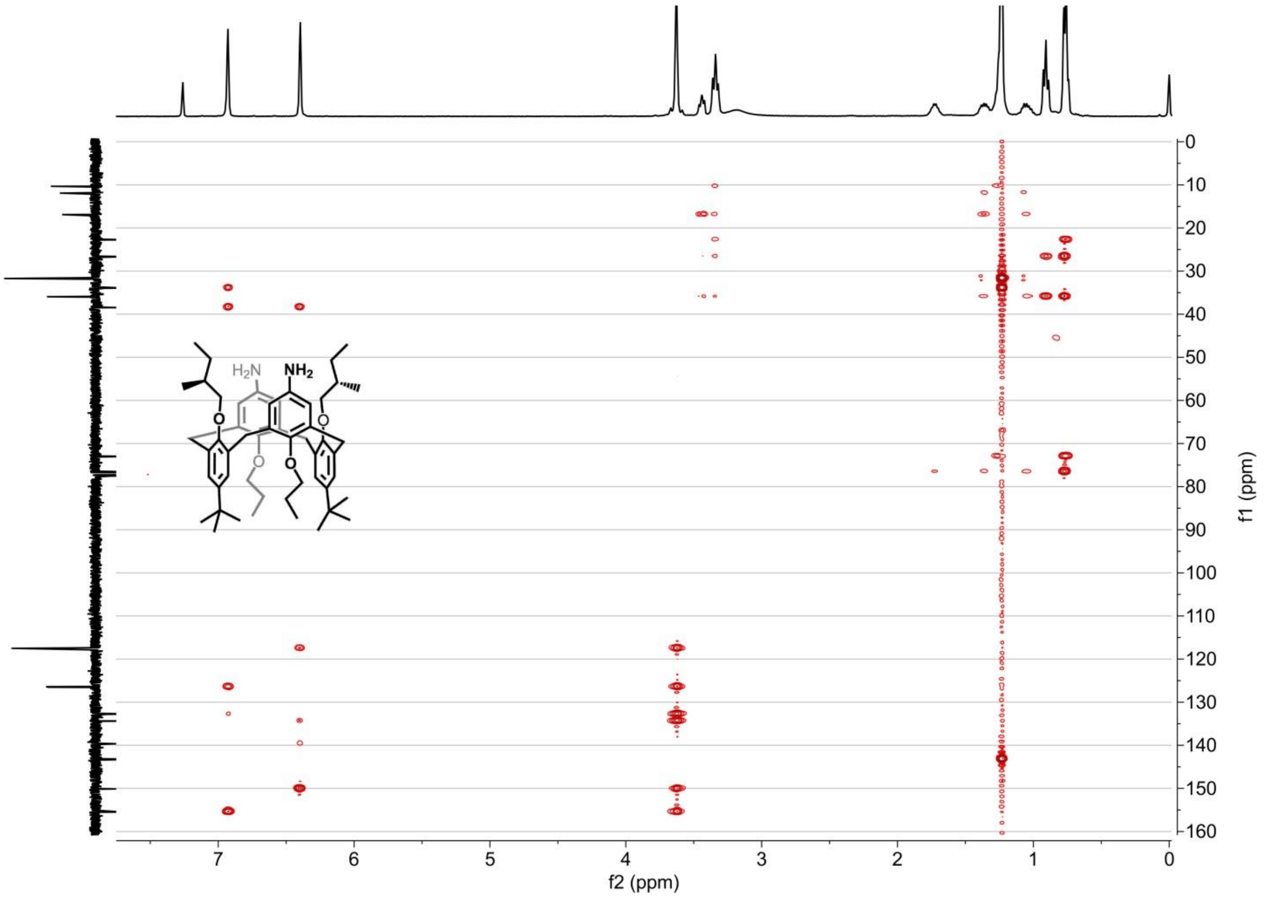

Figure 32. ^{1}H-^{13}C HMBC NMR of compound **6** ($CDCl_3$, 400.1 and 100.6 MHz, 298 K).

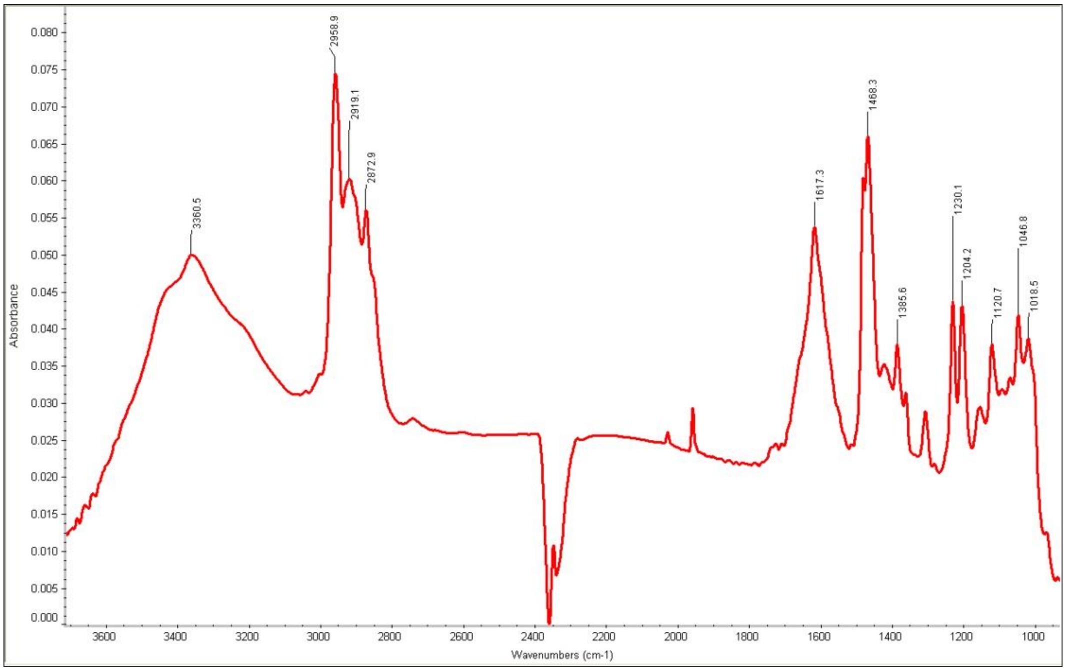

Figure 33. IR of compound **6** (KBr).

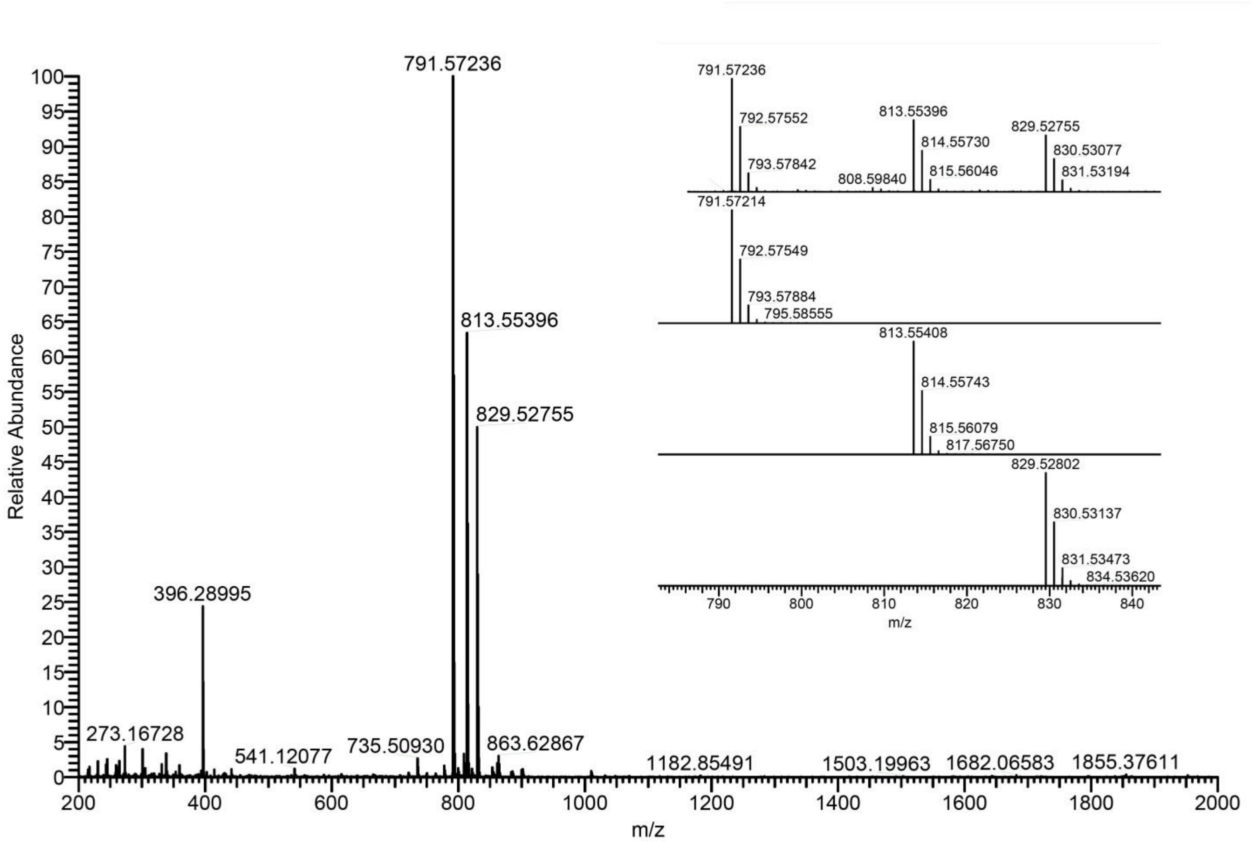

Figure 34. HRMS-ESI of compound **6** (($C_{52}H_{74}N_2O_4$) m/z calcd: 791.5721 $[M+H]^+$, 813.5541 $[M+Na]^+$, 829.5280 $[M+K]^+$).

Compound 7a

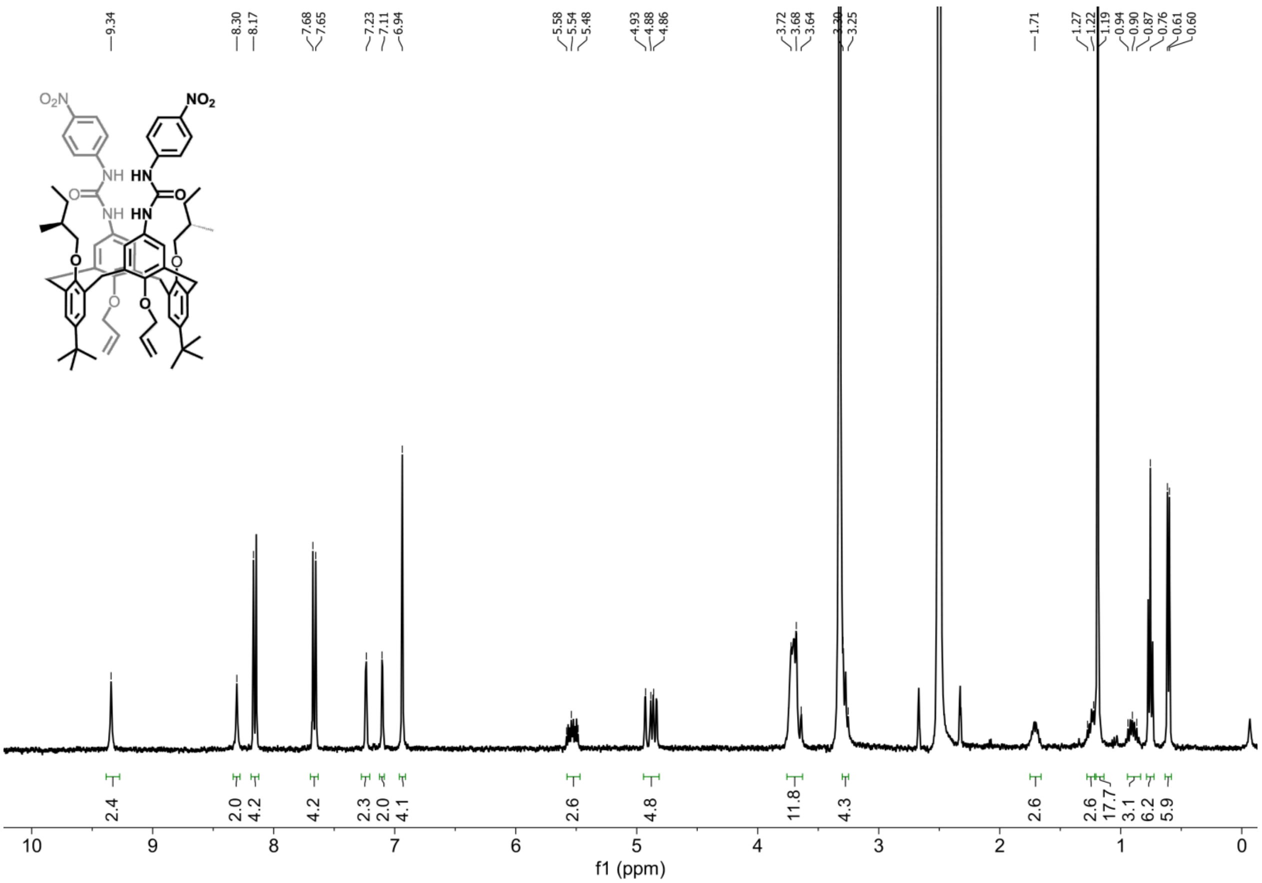

Figure 35. ^1H NMR of compound **7a** (DMSO-d_6, 400.1 MHz, 298 K).

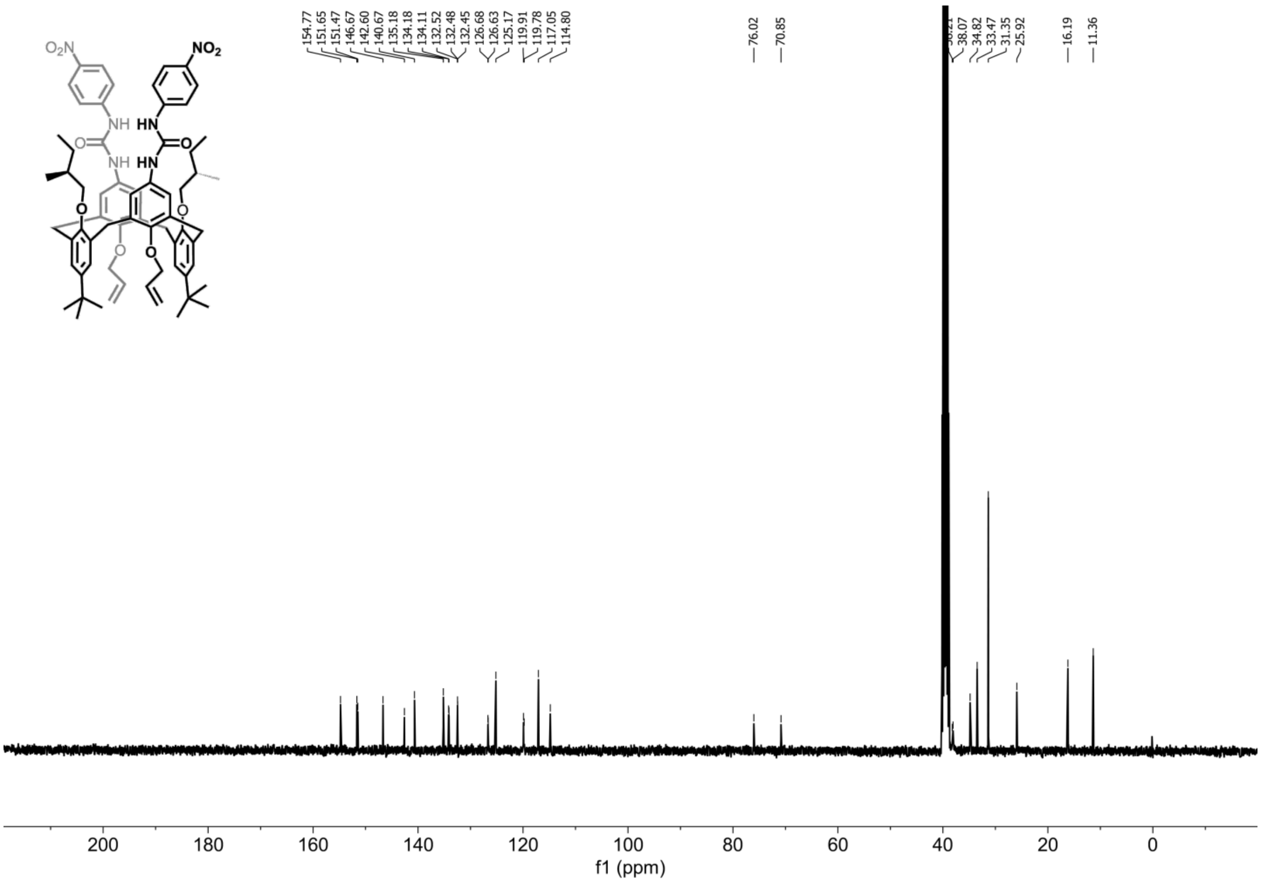

Figure 36. ^{13}C NMR of compound **7a** (DMSO-d_6, 100.6 MHz, 298 K).

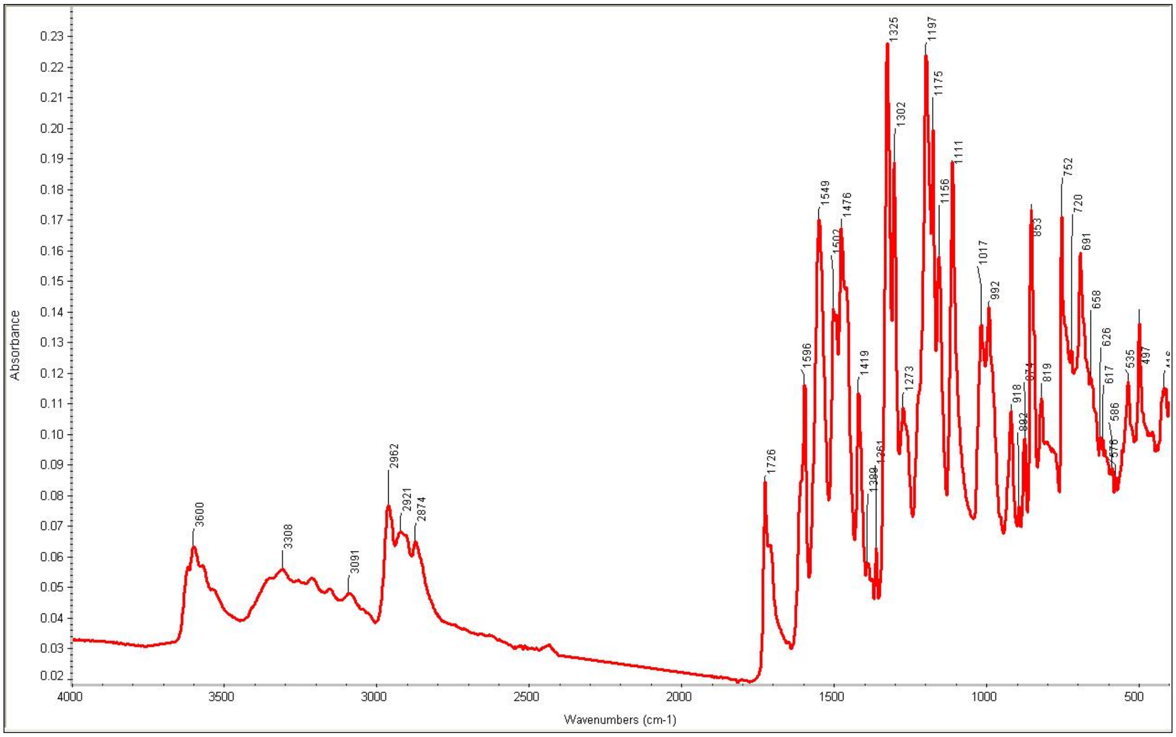

Figure 37. IR of compound **7a** (KBr).

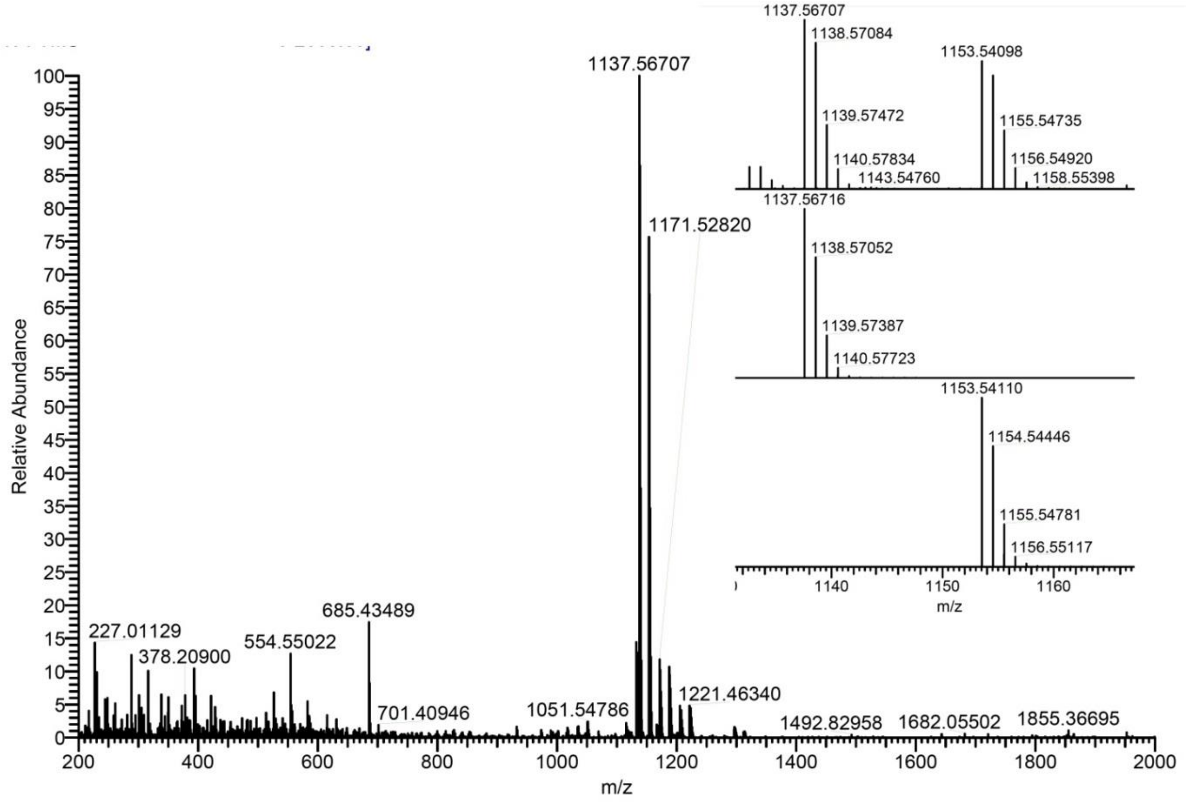

Figure 38. HRMS-ESI of compound **7a** (($C_{66}H_{78}N_6O_{10}$) m/z calcd: 1137.5672 $[M+Na]^+$, 1153.5411 $[M+K]^+$).

Compound 7b

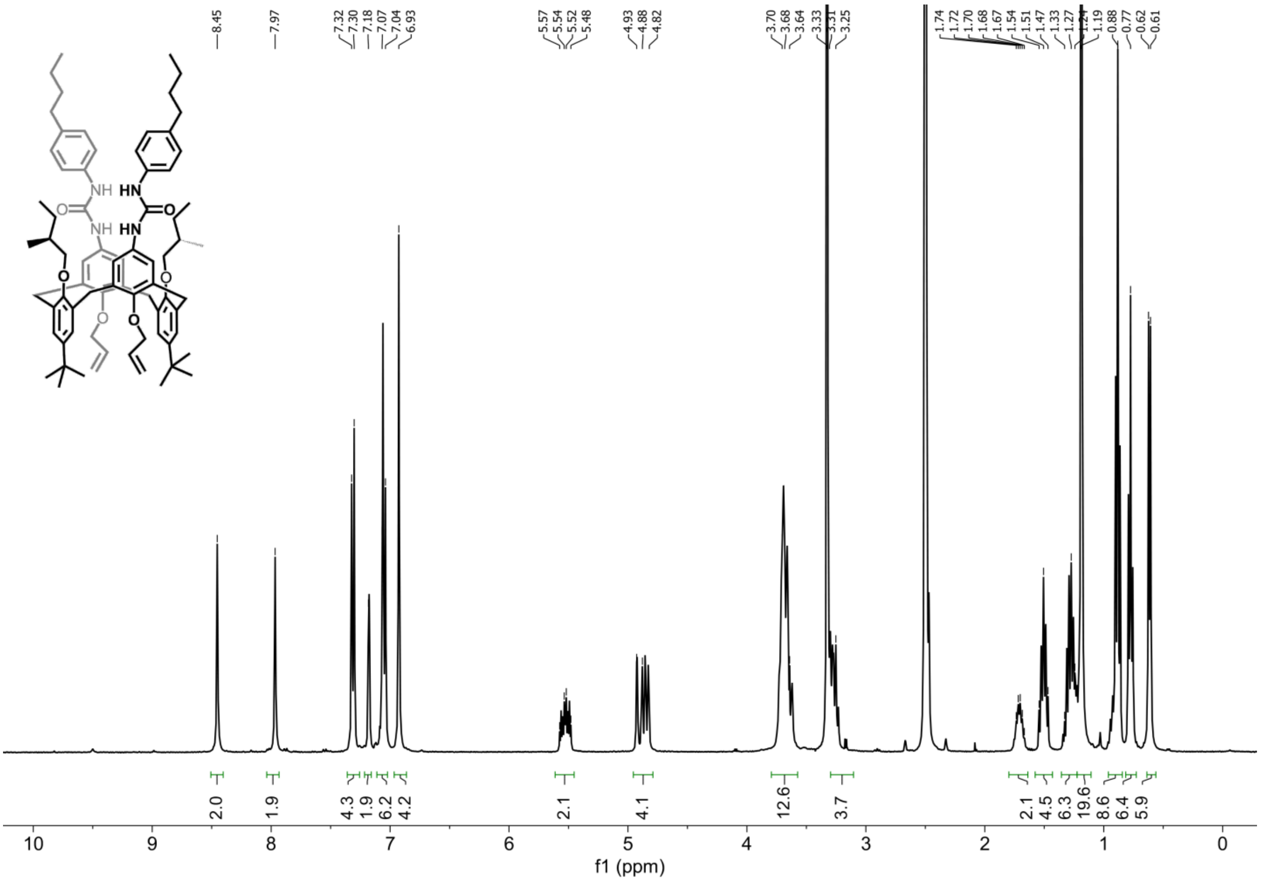

Figure 39. ^{1}H NMR of compound **7b** (DMSO-d_6, 400.1 MHz, 298 K).

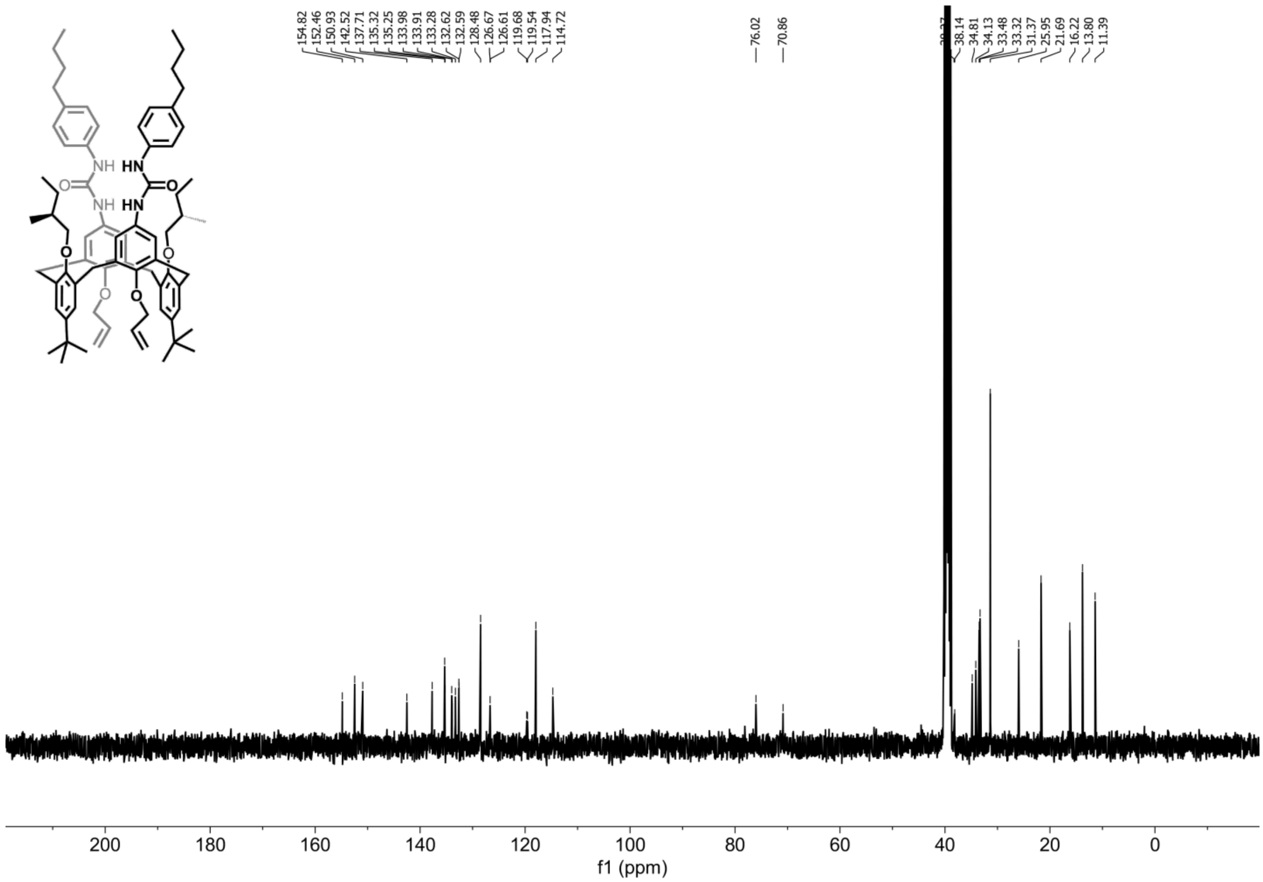

Figure 40. ^{13}C NMR of compound **7b** (DMSO-d_6, 100.6 MHz, 298 K).

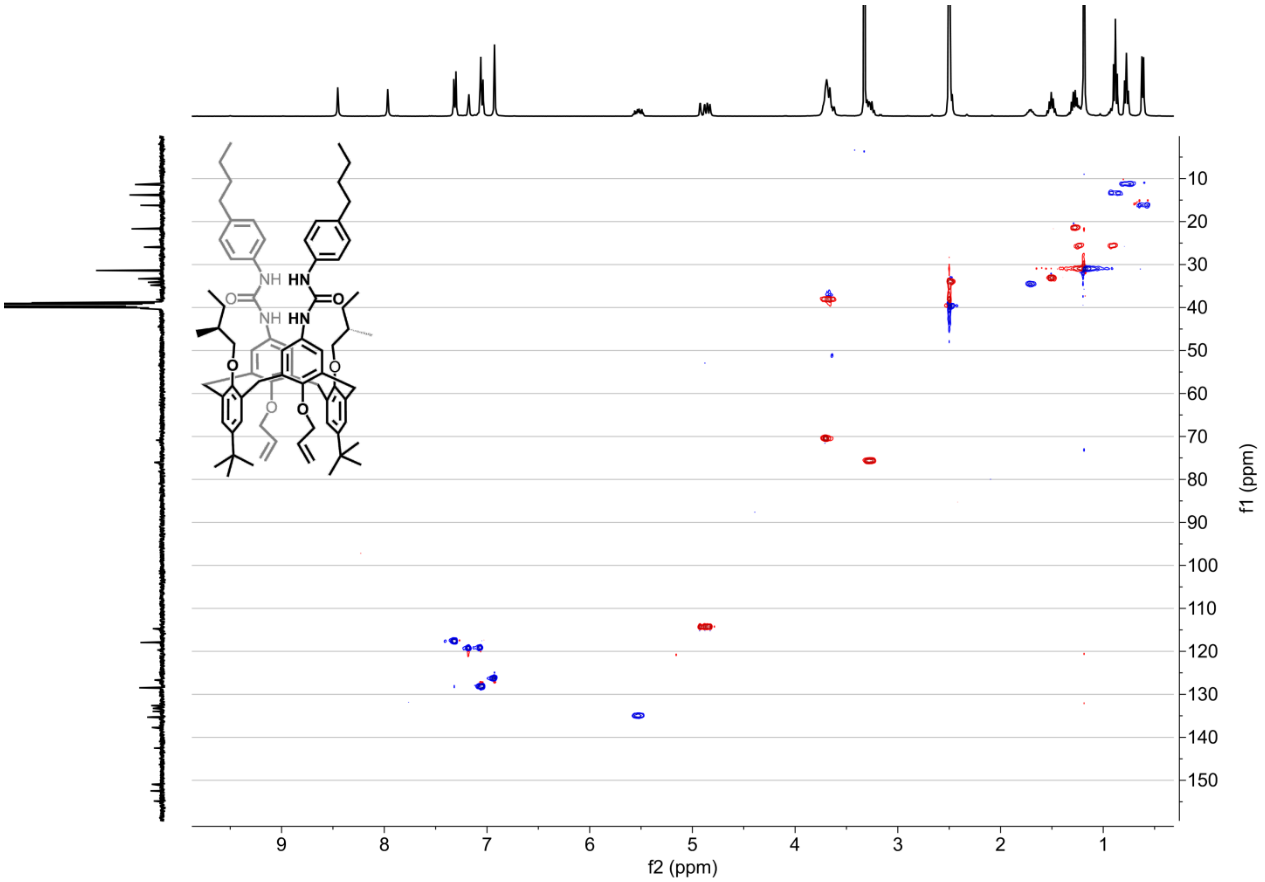

Figure 41. ^{1}H-^{13}C HSQC NMR of compound **7b** (DMSO-d_6, 400.1 and 100.6 MHz, 298 K).

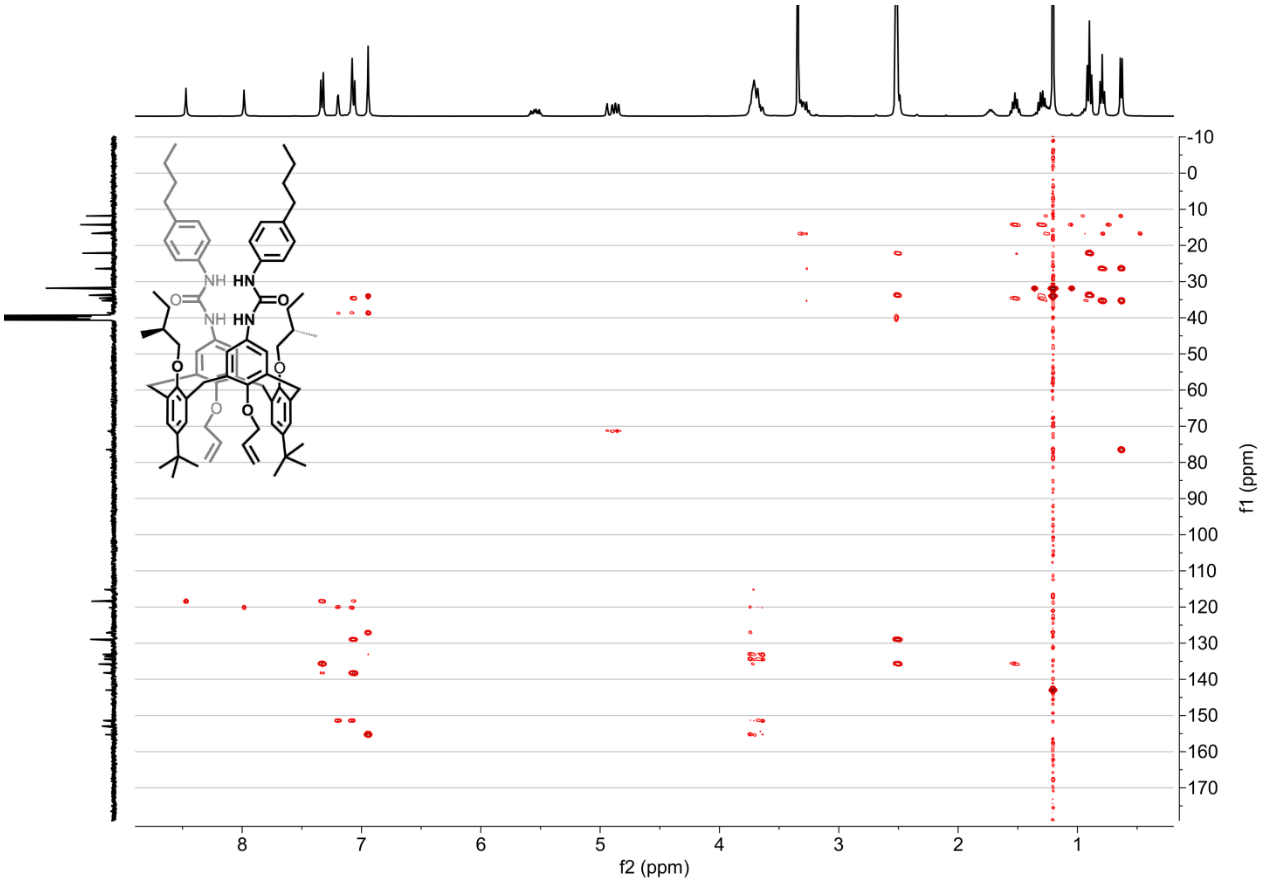

Figure 42. ^{1}H-^{13}C HMBC NMR of compound **7b** (DMSO-d_6, 400.1 and 100.6 MHz, 298 K).

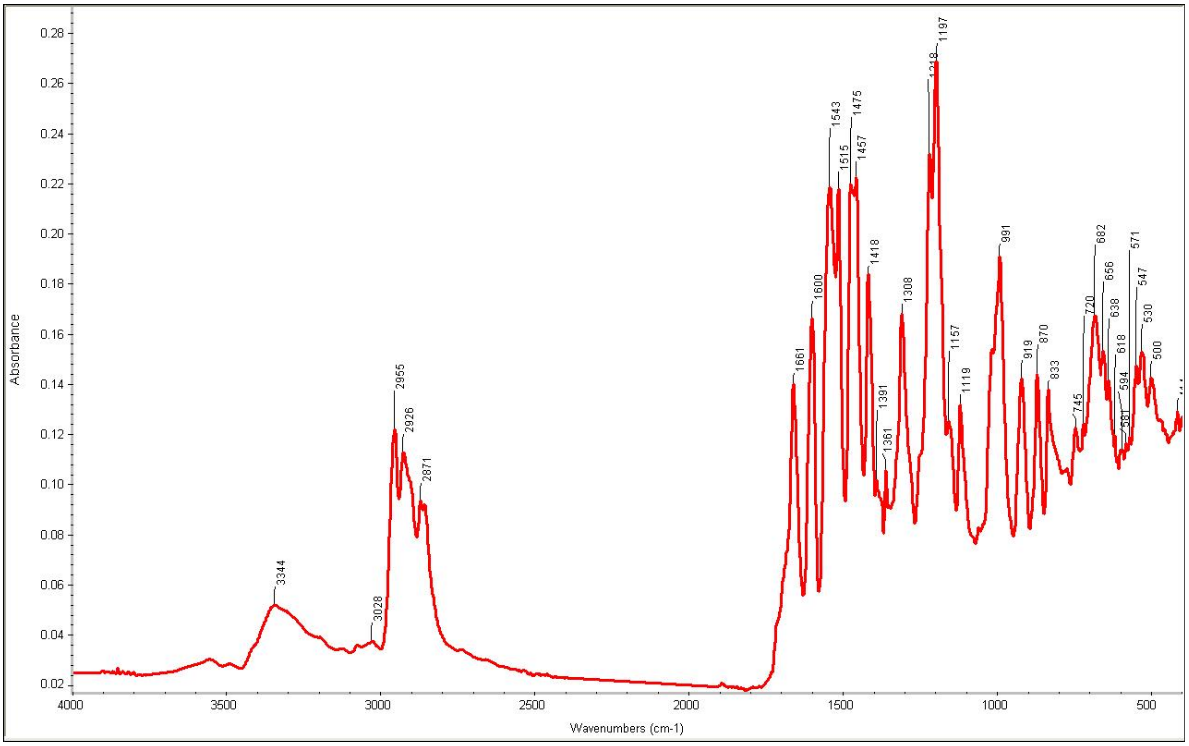

Figure 43. IR of compound **7b** (KBr).

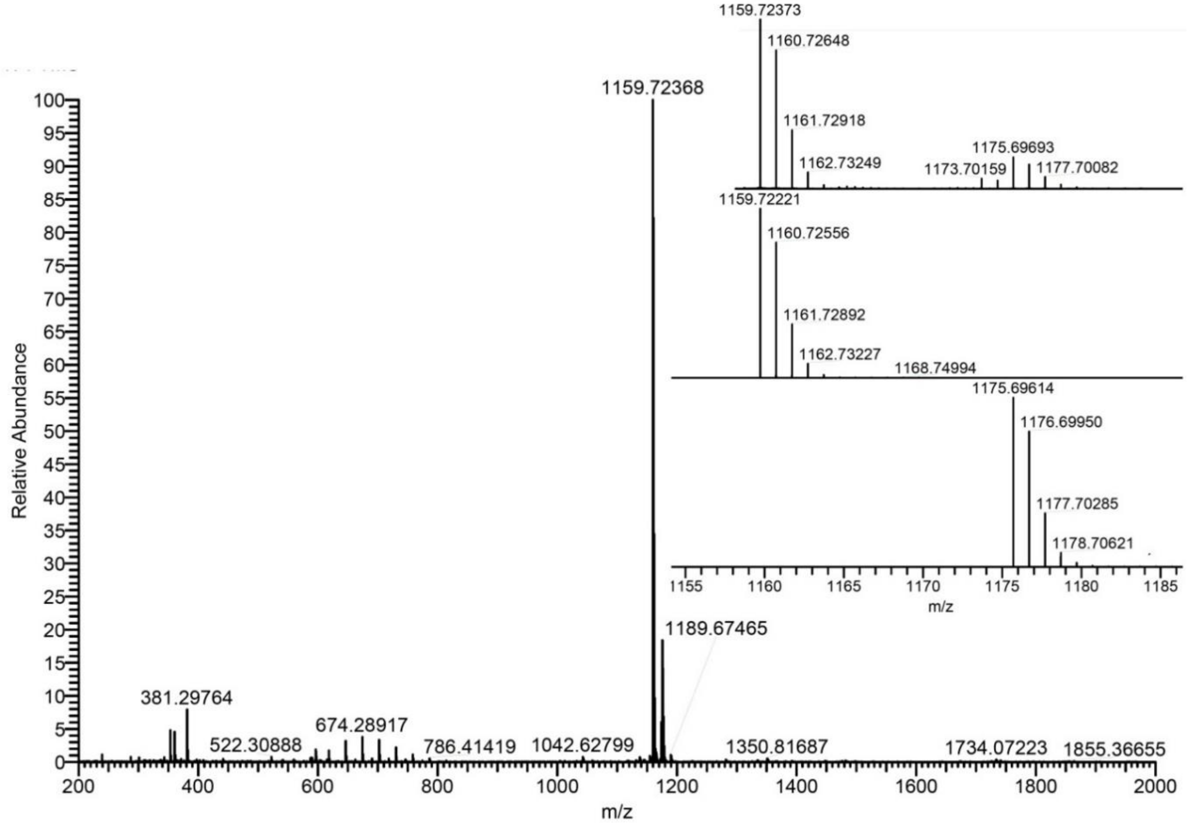

Figure 44. HRMS-ESI of compound **7b** (($C_{74}H_{96}N_4O_6$) m/z calcd: 1159.7222 [M+Na]$^+$, 1175.6961 [M+K]$^+$).

Compound 7c

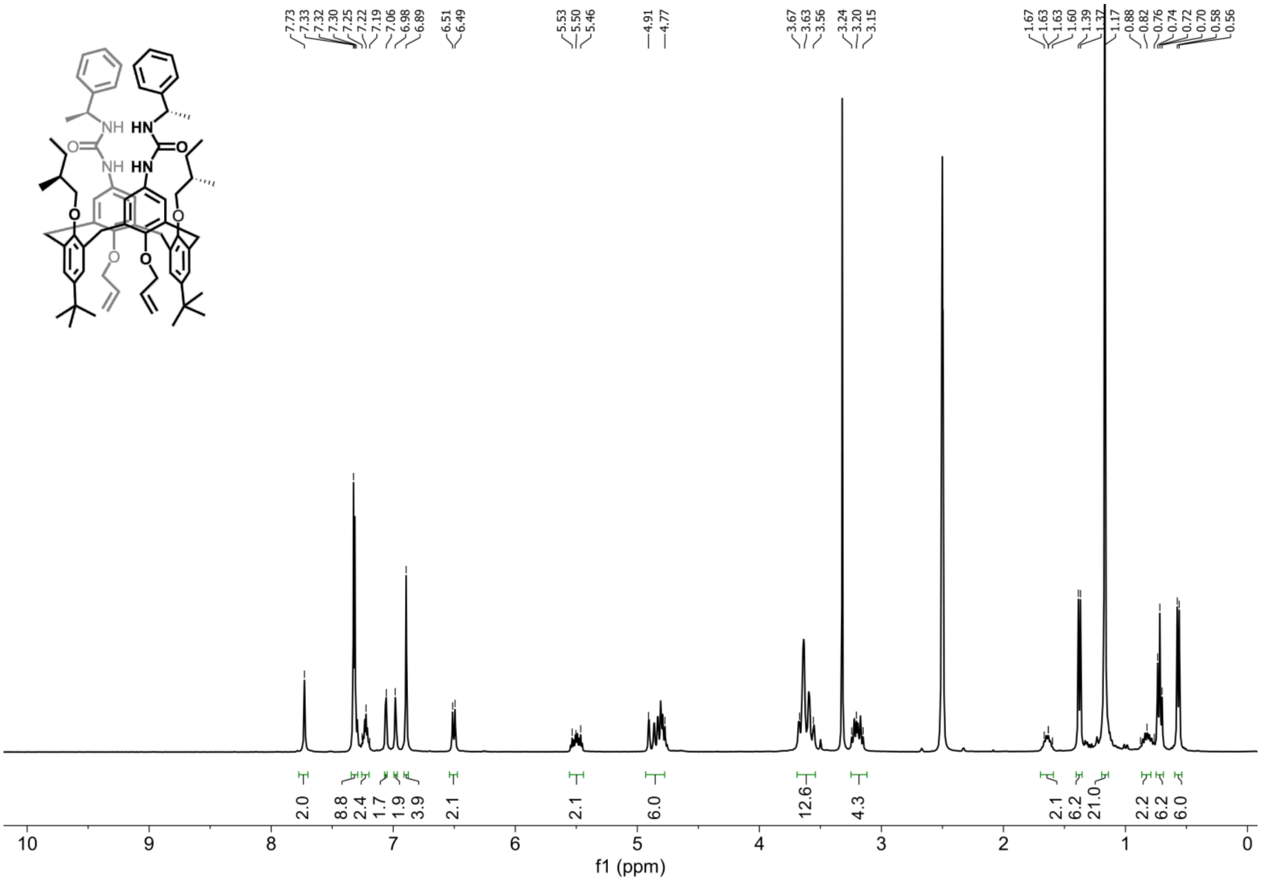

Figure 45. ^{1}H NMR of compound **7c** (DMSO-d_6, 400.1 MHz, 298 K).

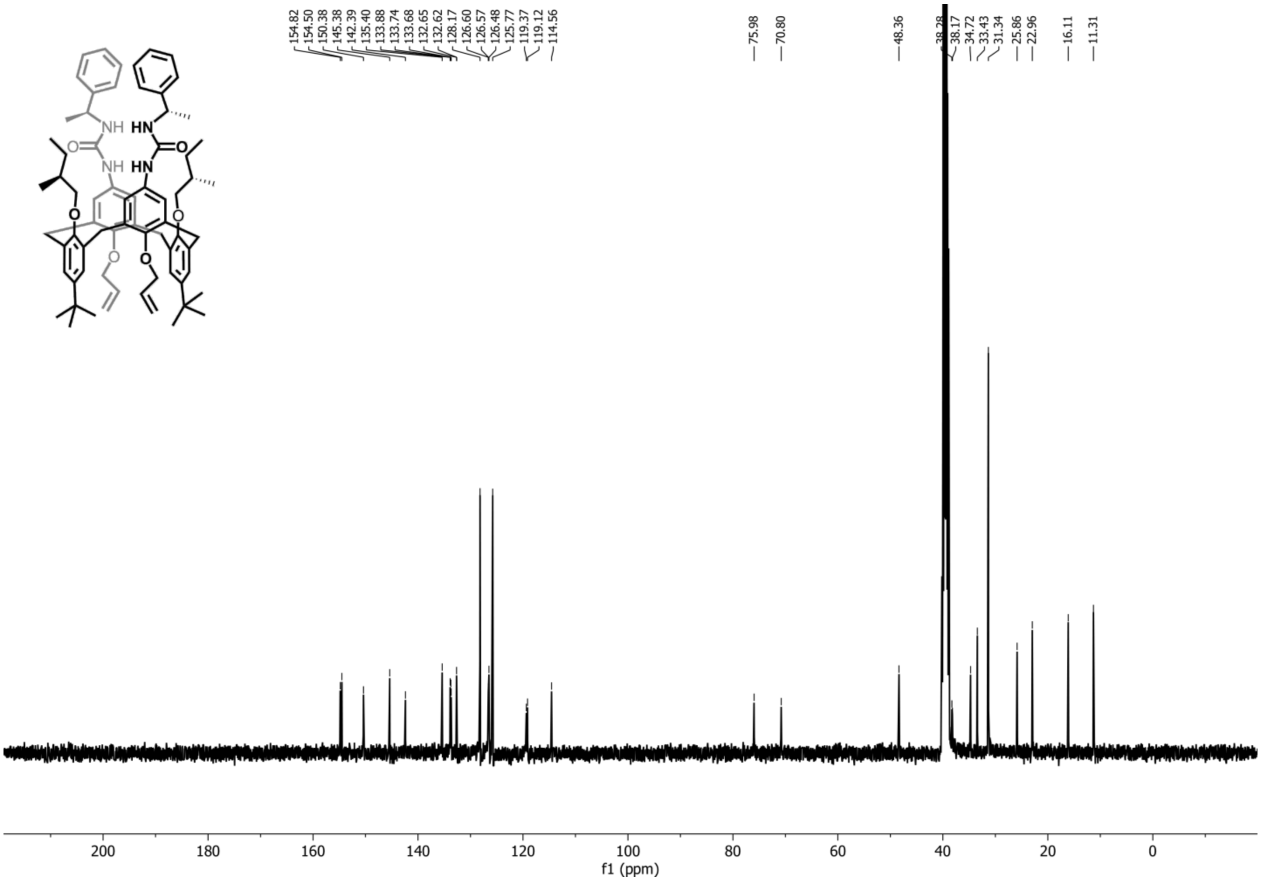

Figure 46. ^{13}C NMR of compound **7c** (DMSO-d_6, 100.6 MHz, 298 K).

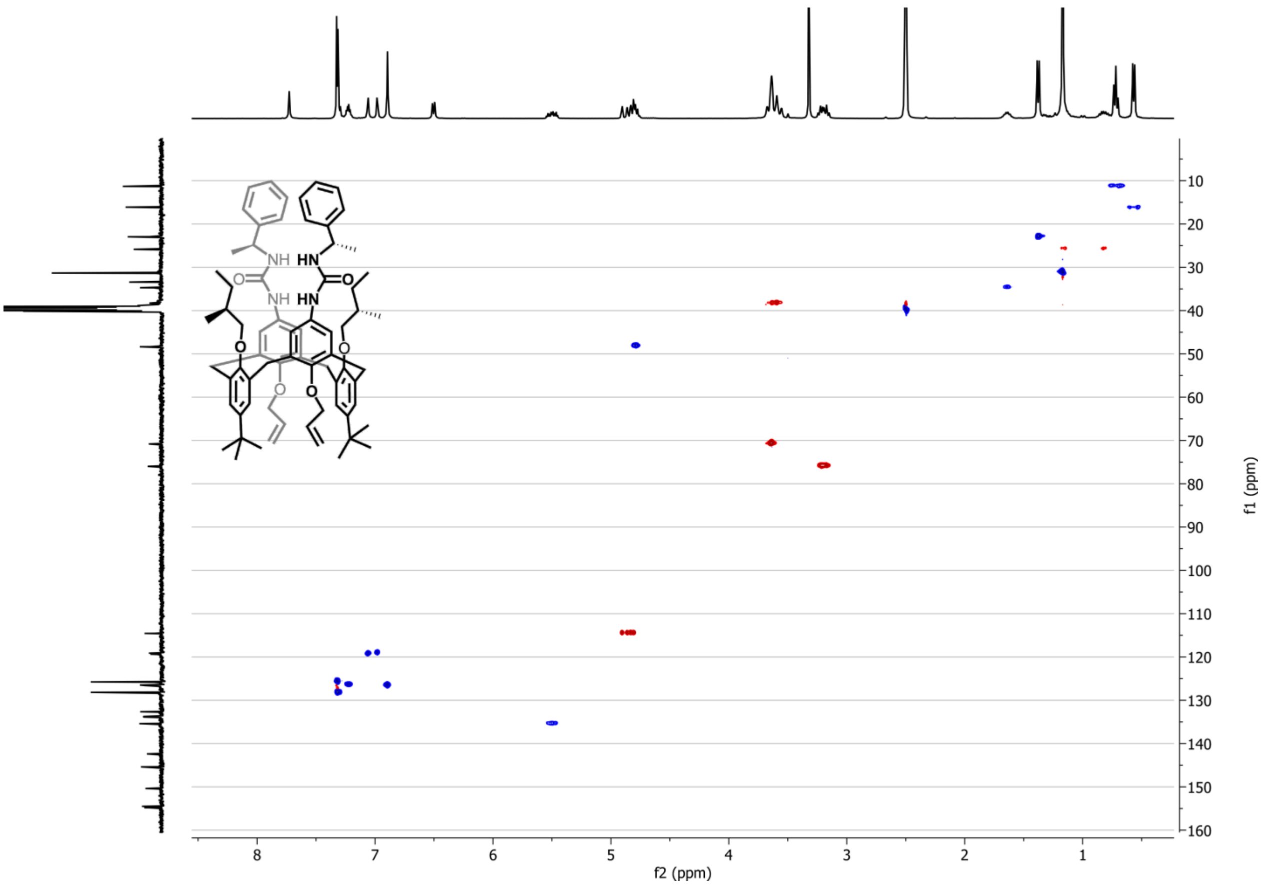

Figure 47. ^{1}H-^{13}C HSQC NMR of compound **7c** (DMSO-d_6, 400.1 and 100.6 MHz, 298 K).

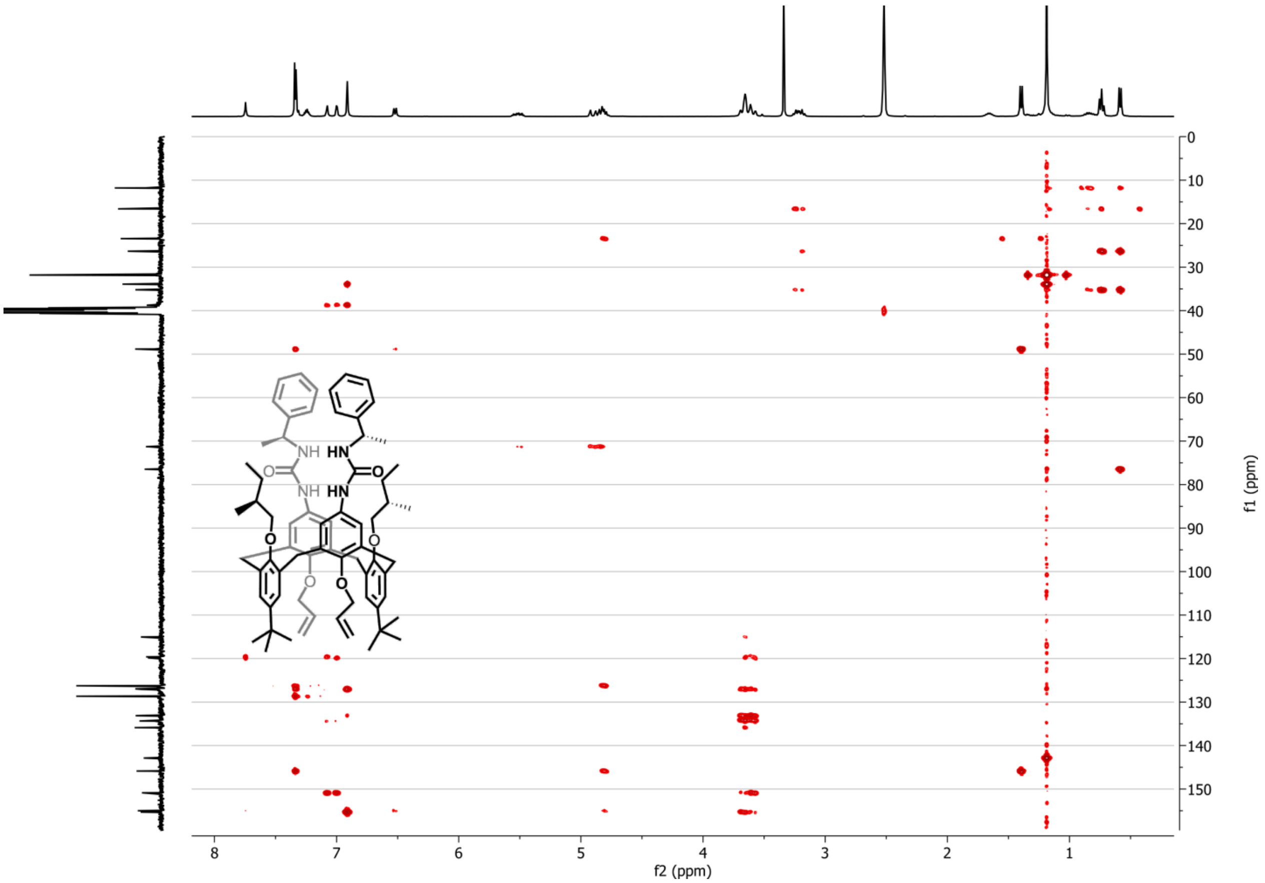

Figure 48. ^{1}H-^{13}C HMBC NMR of compound **7c** (DMSO-d_6, 400.1 and 100.6 MHz, 298 K).

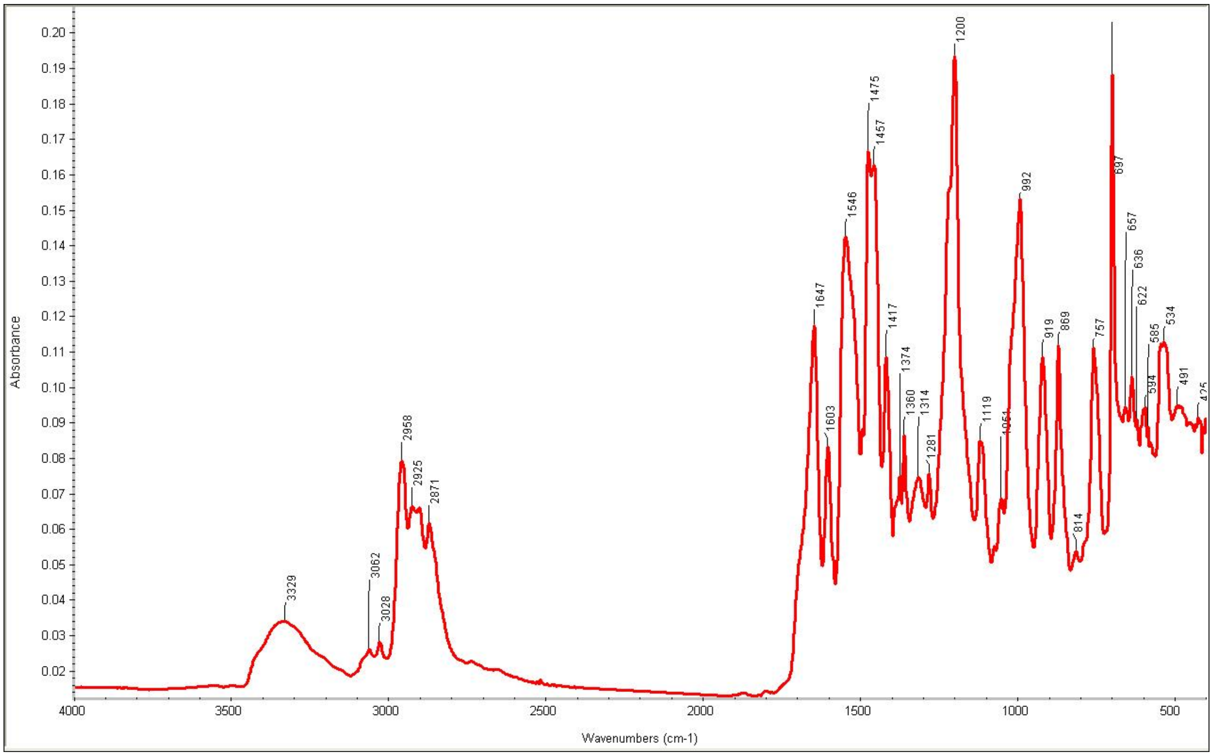

Figure 49. IR of compound **7c** (KBr).

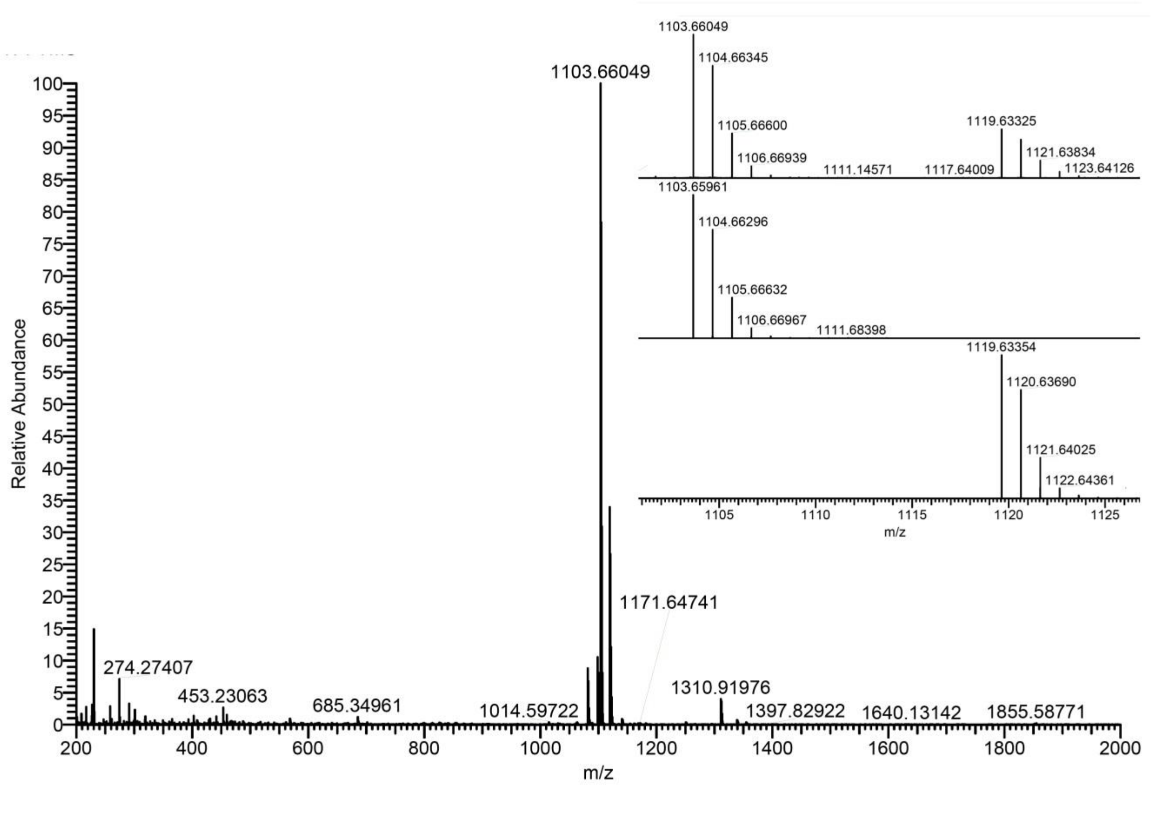

Figure 50. HRMS-ESI of compound **7c** (($C_{70}H_{88}N_4O_6$) m/z calcd: 1103.6596 $[M+Na]^+$, 1119.6335 $[M+K]^+$).

Compound 7d

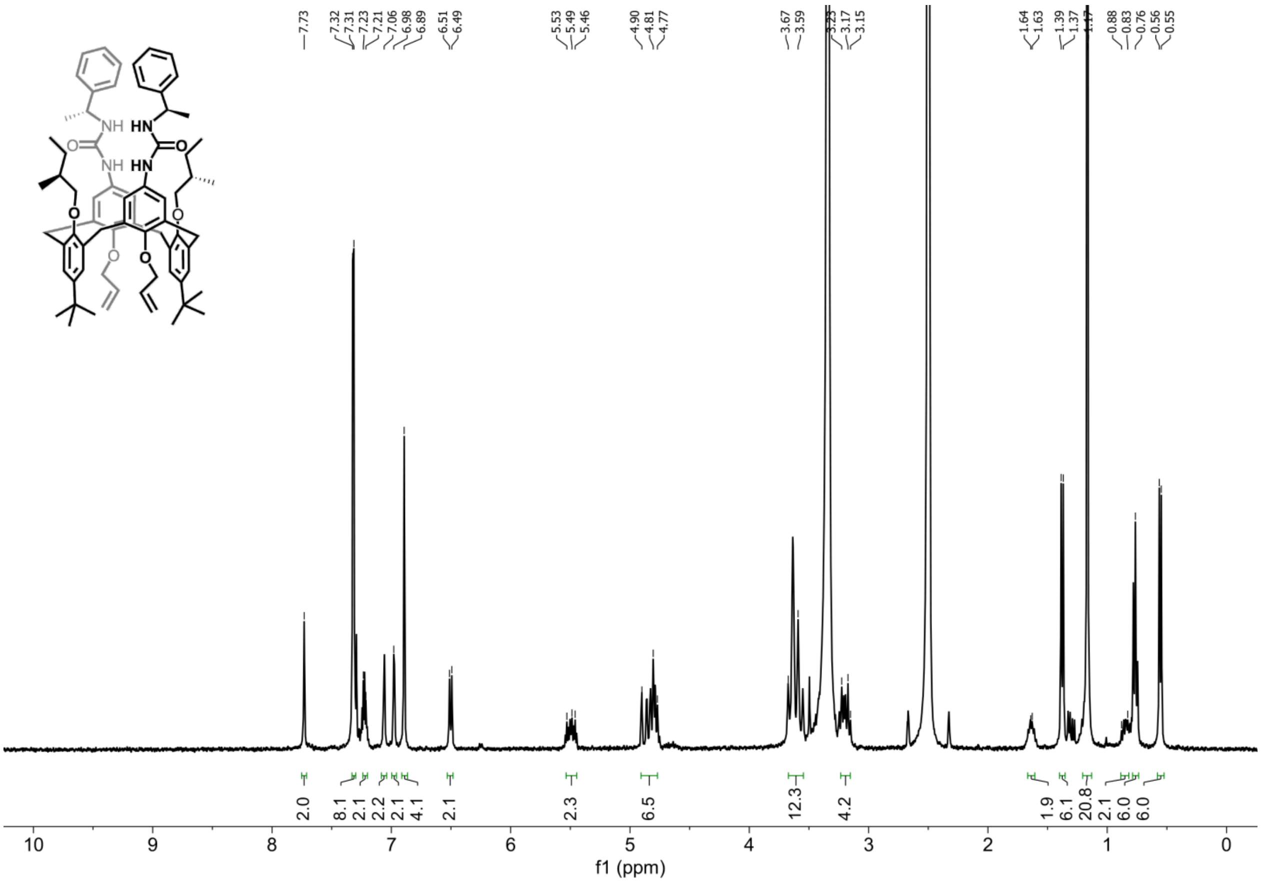

Figure 51. ^{1}H NMR of compound **7d** (DMSO-d_6, 400.1 MHz, 298 K).

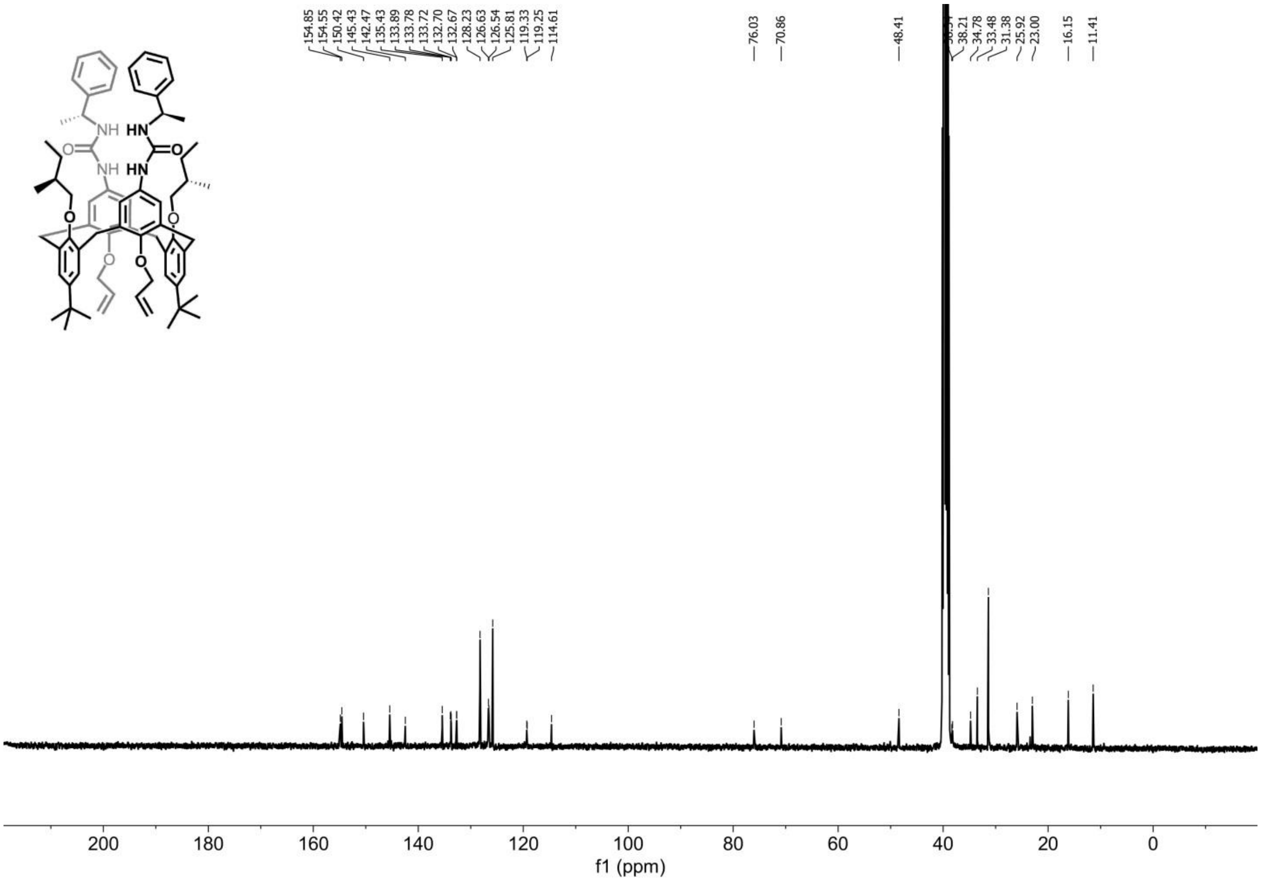

Figure 52. ^{13}C NMR of compound **7d** (DMSO-d_6, 100.6 MHz, 298 K).

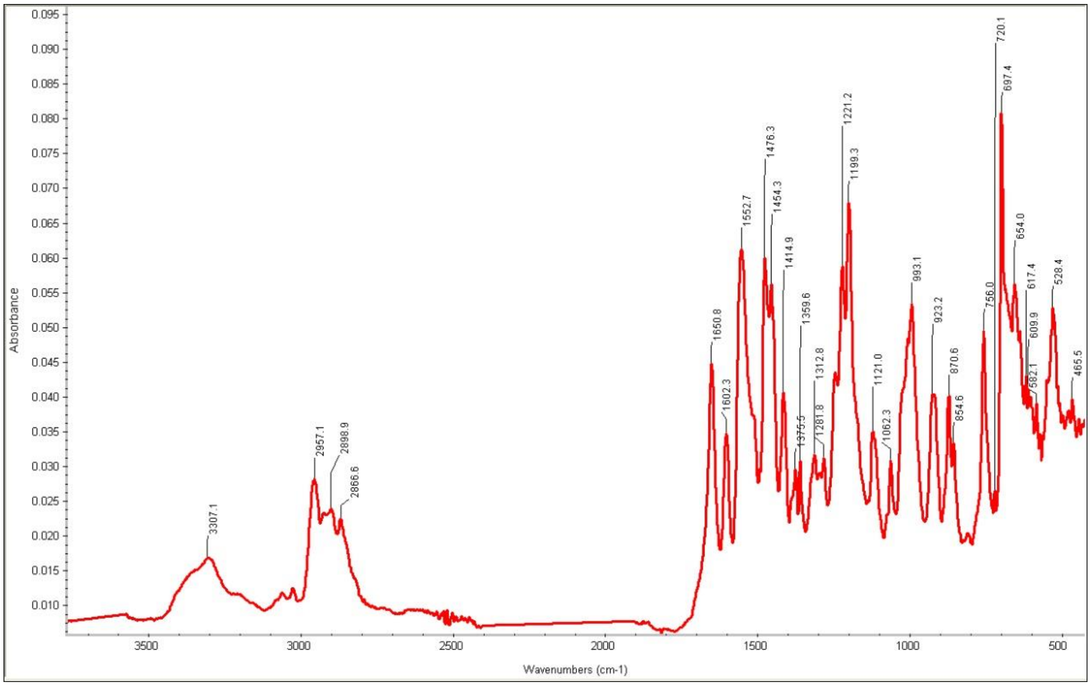

Figure 53. IR of compound **7d** (KBr).

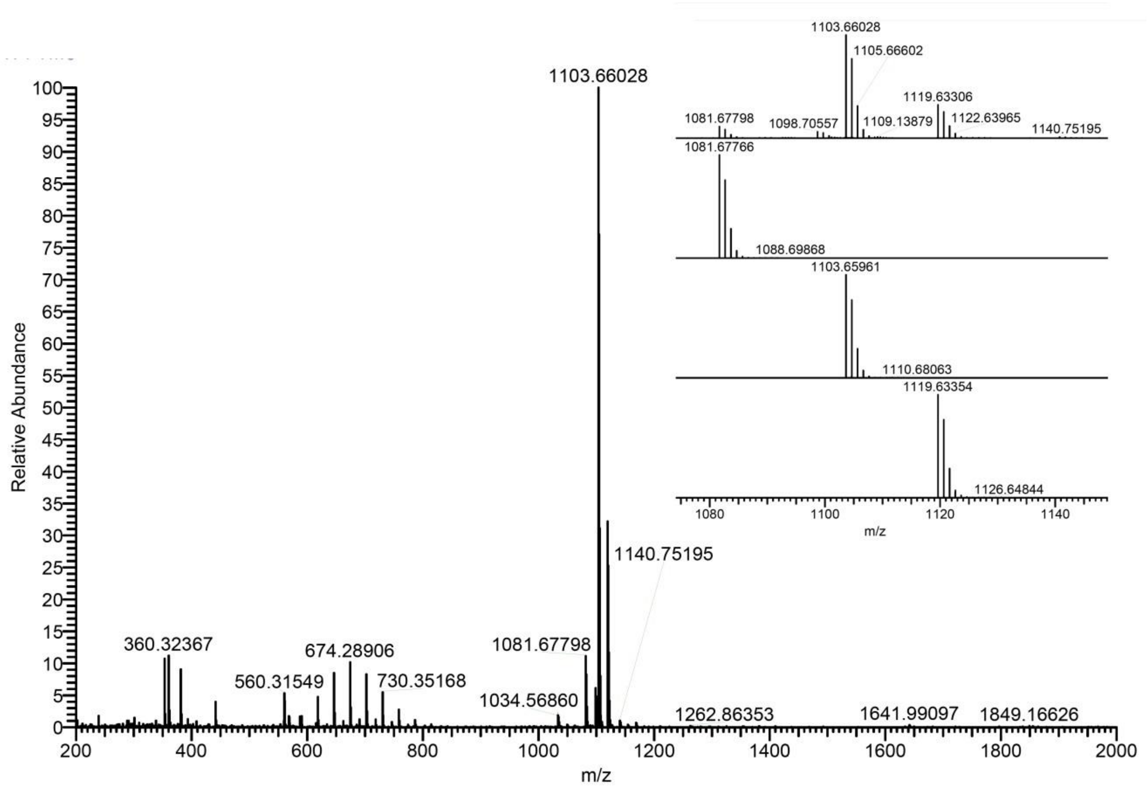

Figure 54. HRMS-ESI of compound **7d** (($C_{70}H_{88}N_4O_6$) m/z calcd: 1081.6777 $[M+H]^+$, 1103.6596 $[M+Na]^+$, 1119.6335 $[M+K]^+$).

Compound 8a

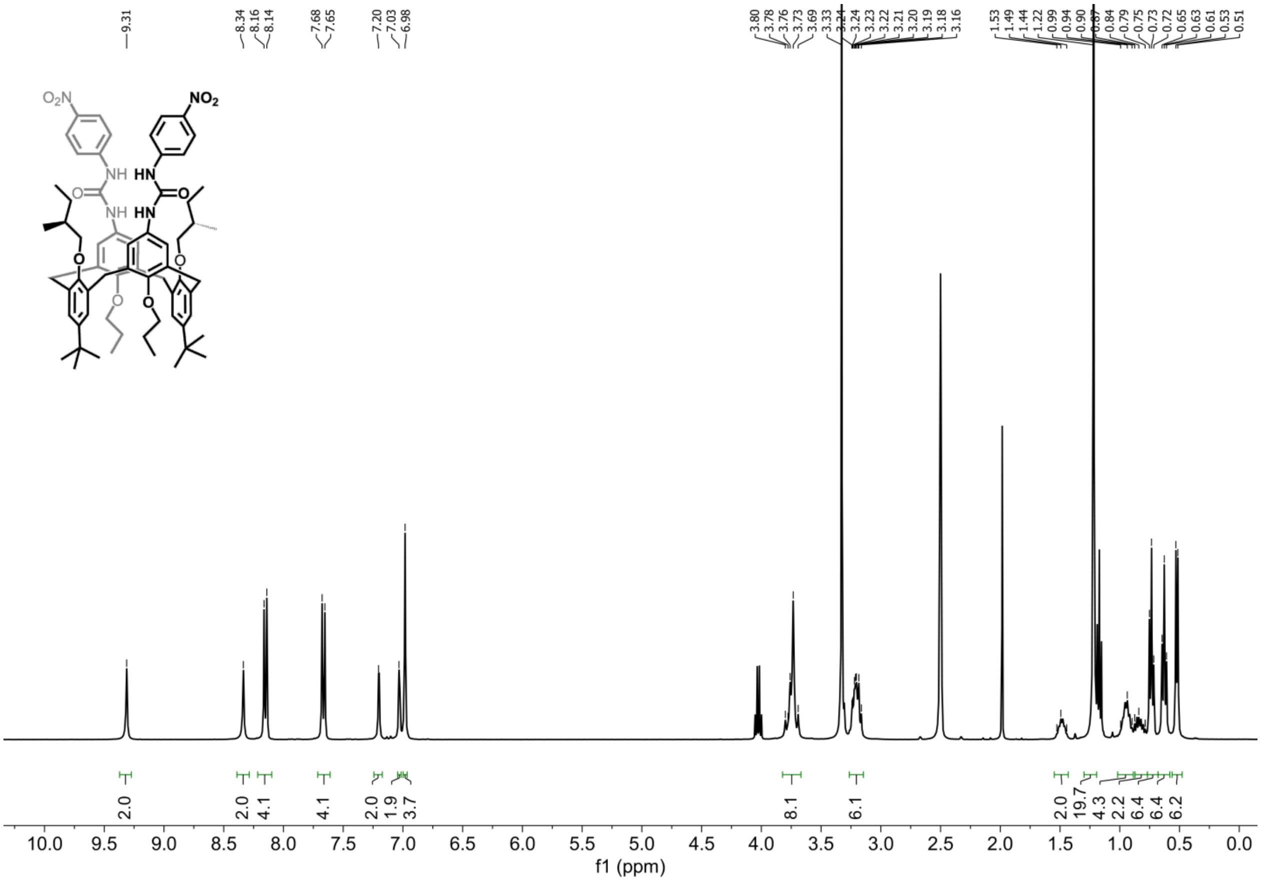

Figure 55. ^{1}H NMR of compound **8a** (DMSO-d_6, 400.1 MHz, 298 K).

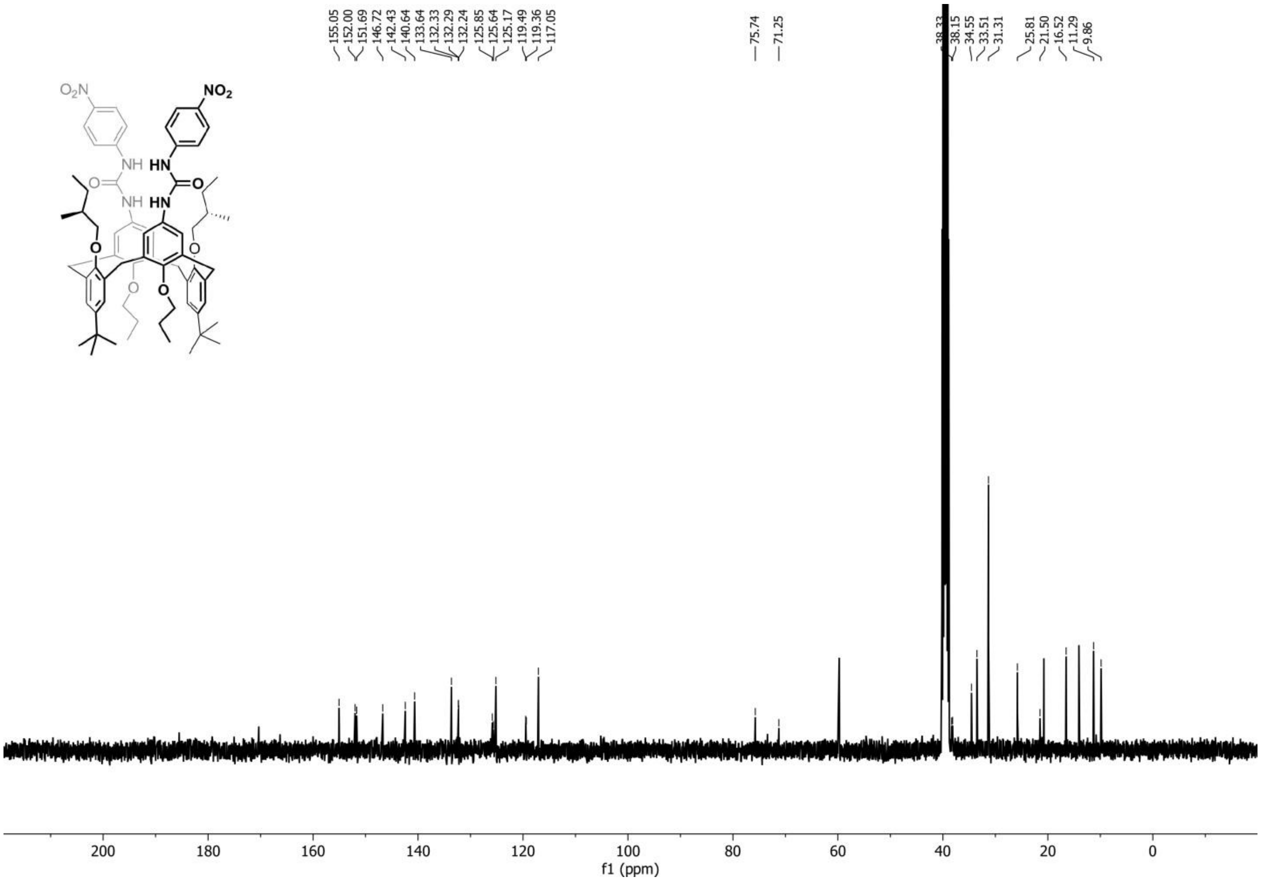

Figure 56. ^{13}C NMR of compound **8a** (DMSO-d_6, 100.6 MHz, 298 K).

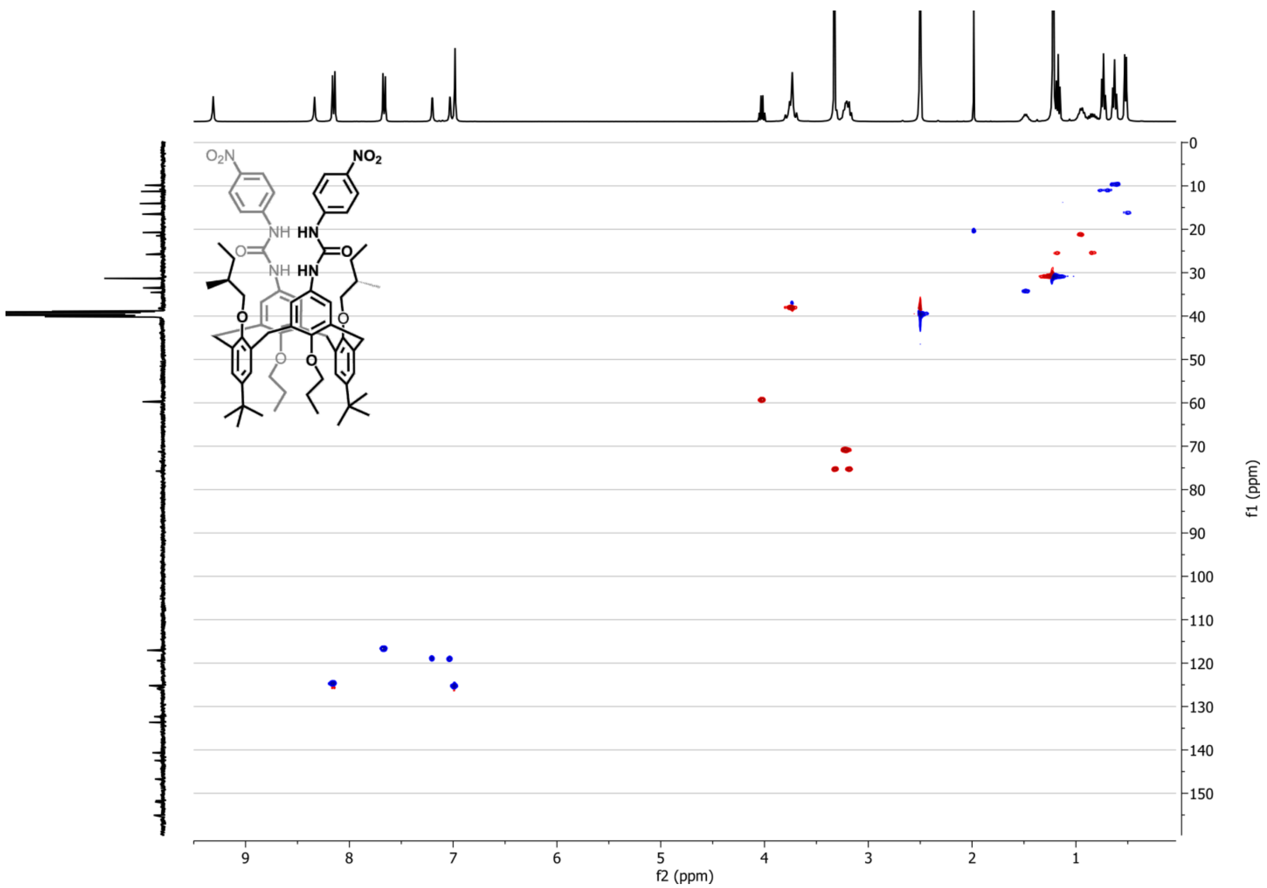

Figure 57. ^{1}H-^{13}C HSQC NMR of compound **8a** (DMSO-d_6, 400.1 and 100.6 MHz, 298 K).

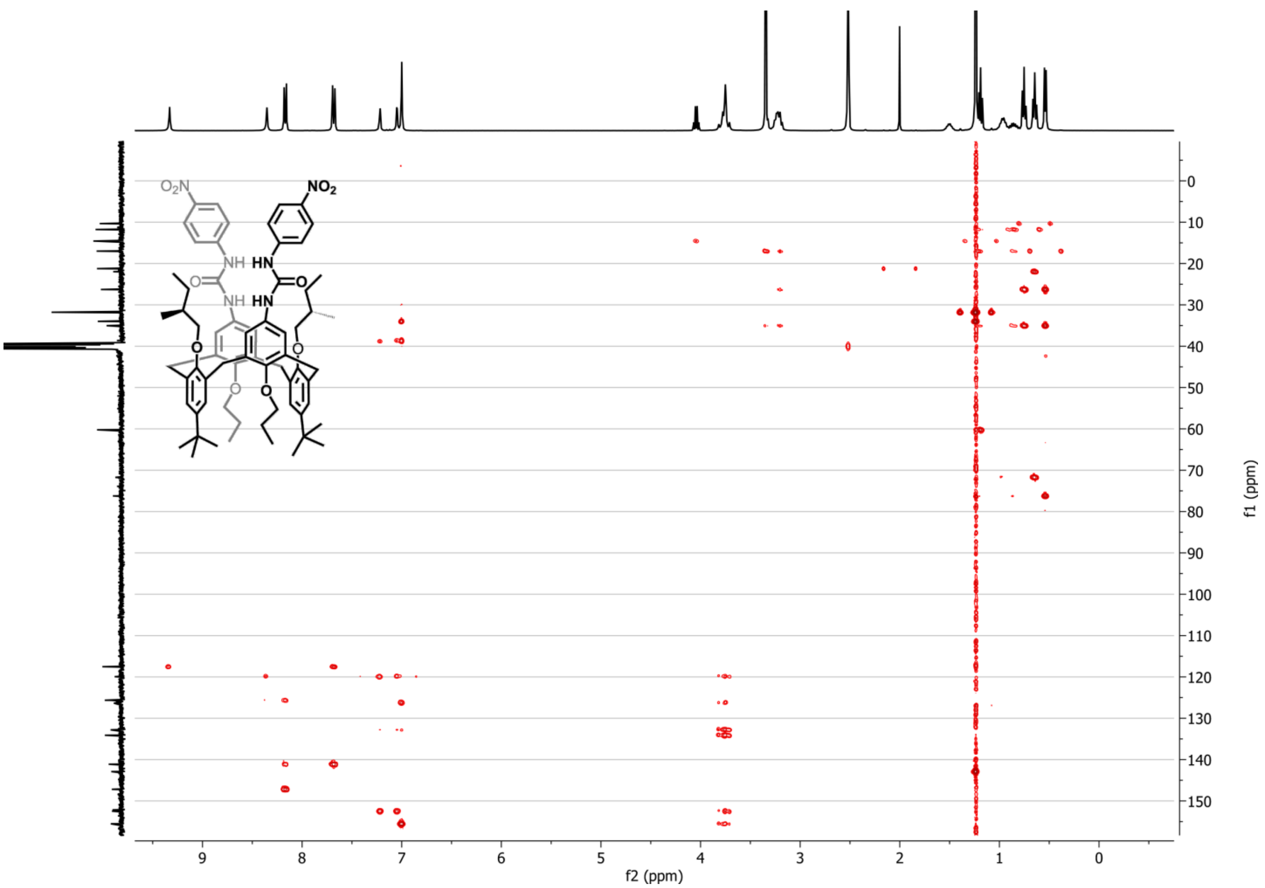

Figure 58. ^{1}H-^{13}C HMBC NMR of compound **8a** (DMSO-d_6, 400.1 and 100.6 MHz, 298 K).

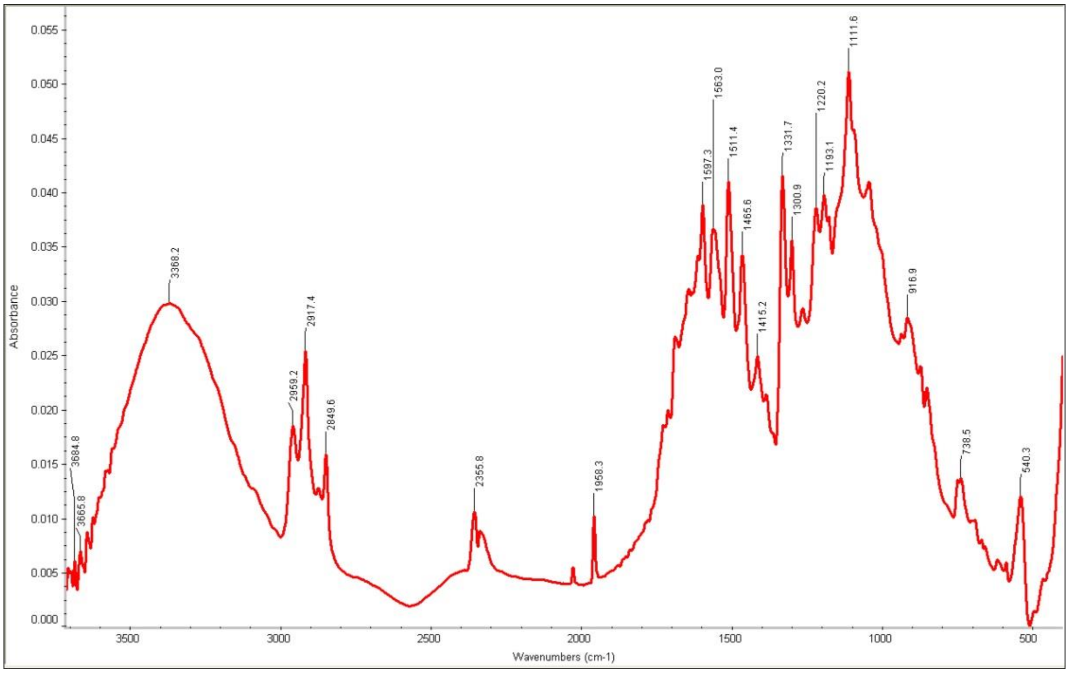

Figure 59. IR of compound **8a** (KBr).

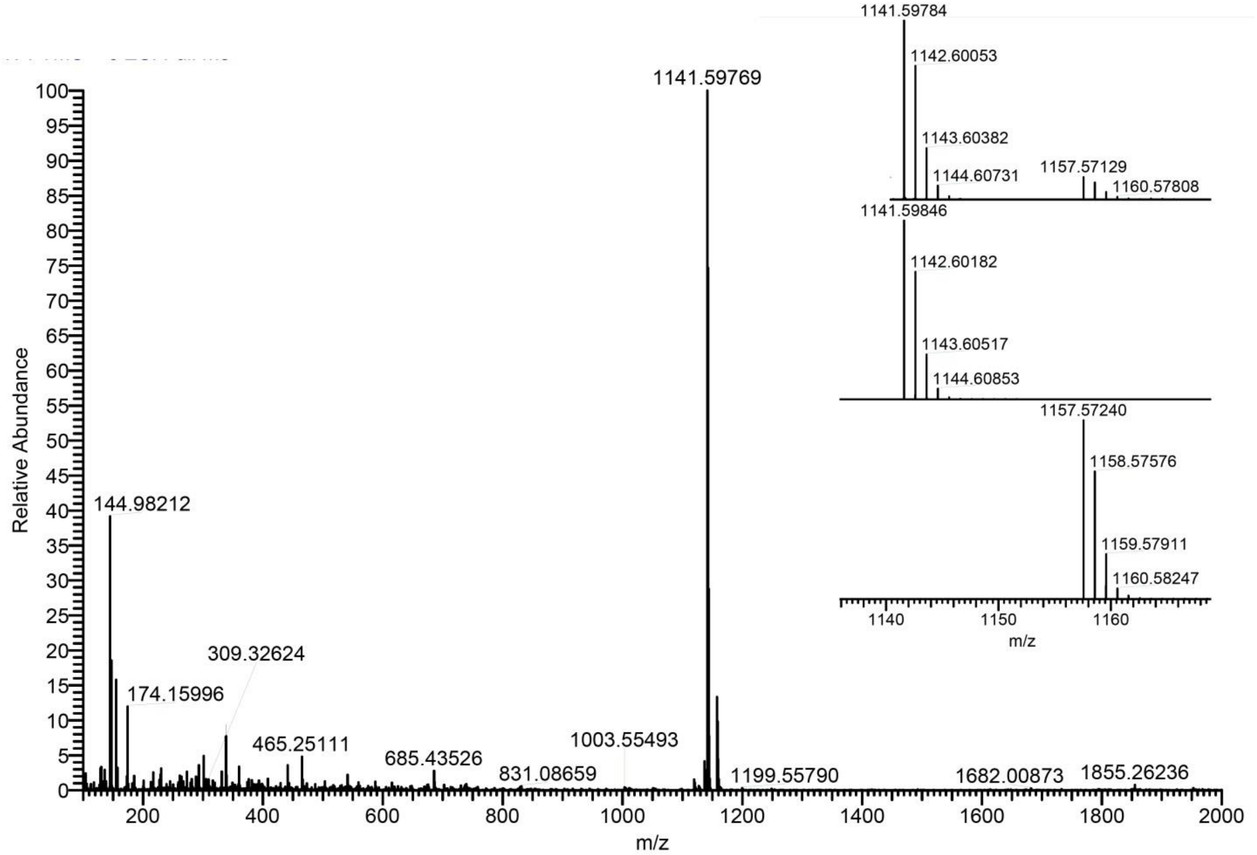

Figure 60. HRMS-ESI of compound **8a** (($C_{66}H_{82}N_6O_{10}$) m/z calcd: 1141.5985 $[M+Na]^+$, 1157.5724 $[M+K]^+$).

Compound 8b

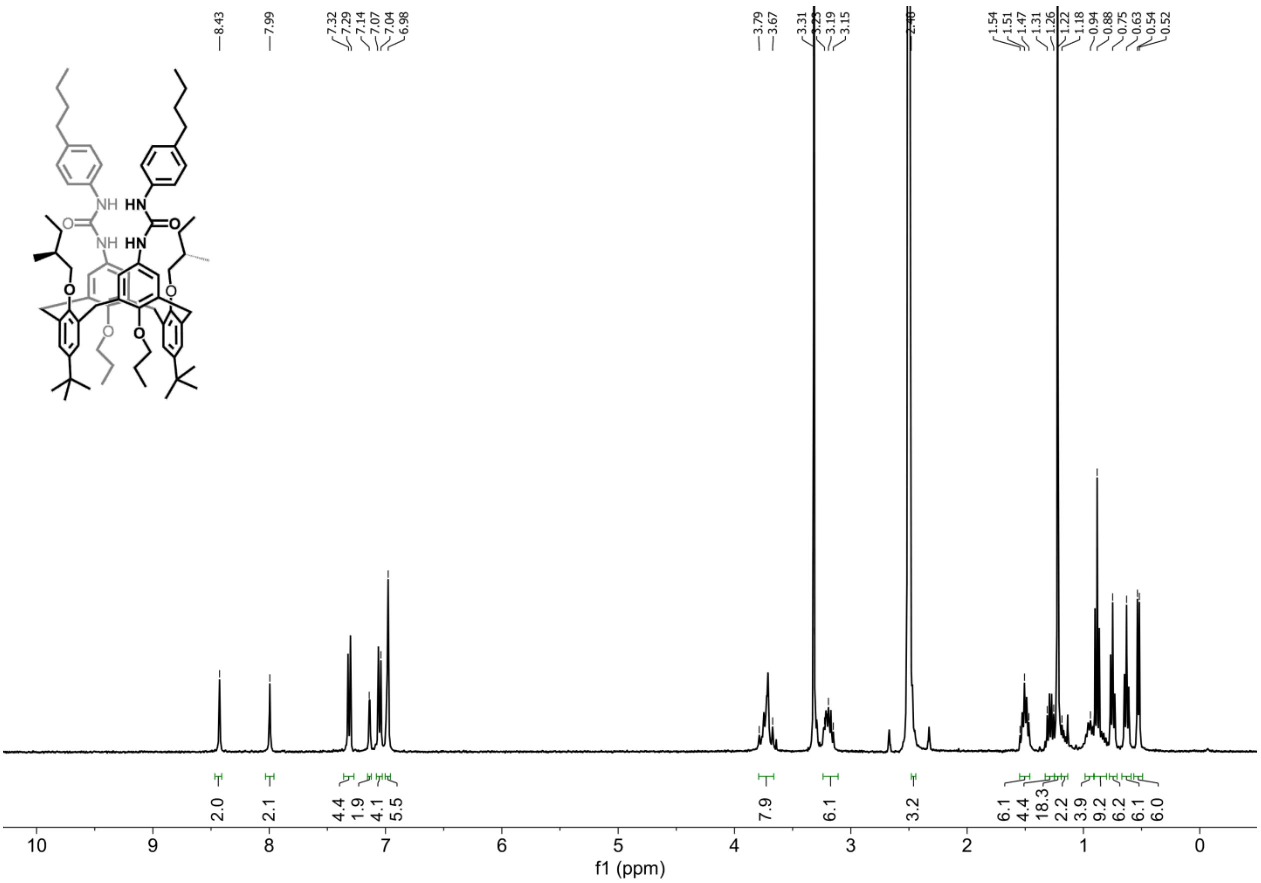

Figure 61. ^{1}H NMR of compound **8b** (DMSO-d_6, 400.1 MHz, 298 K).

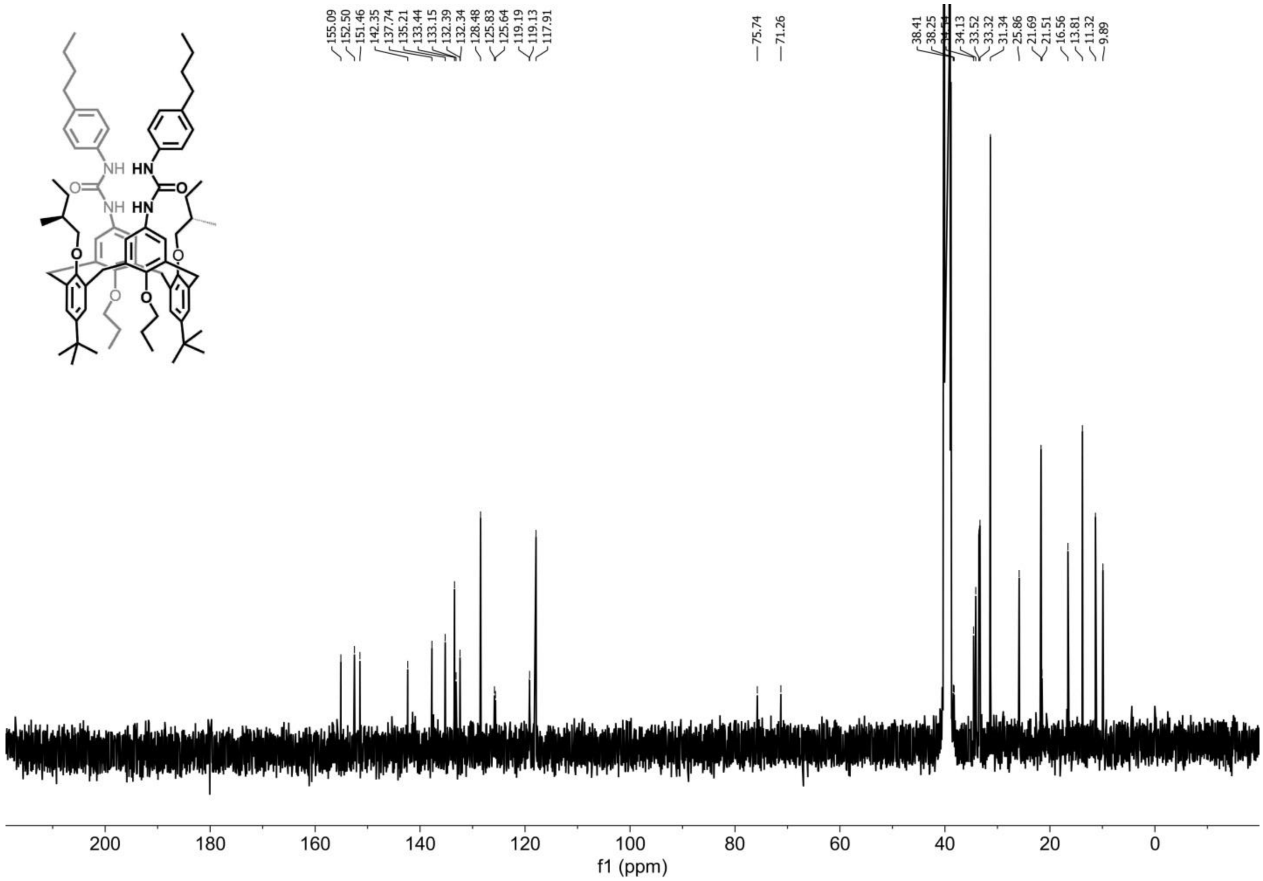

Figure 62. ^{13}C NMR of compound **8b** (DMSO-d_6, 100.6 MHz, 298 K).

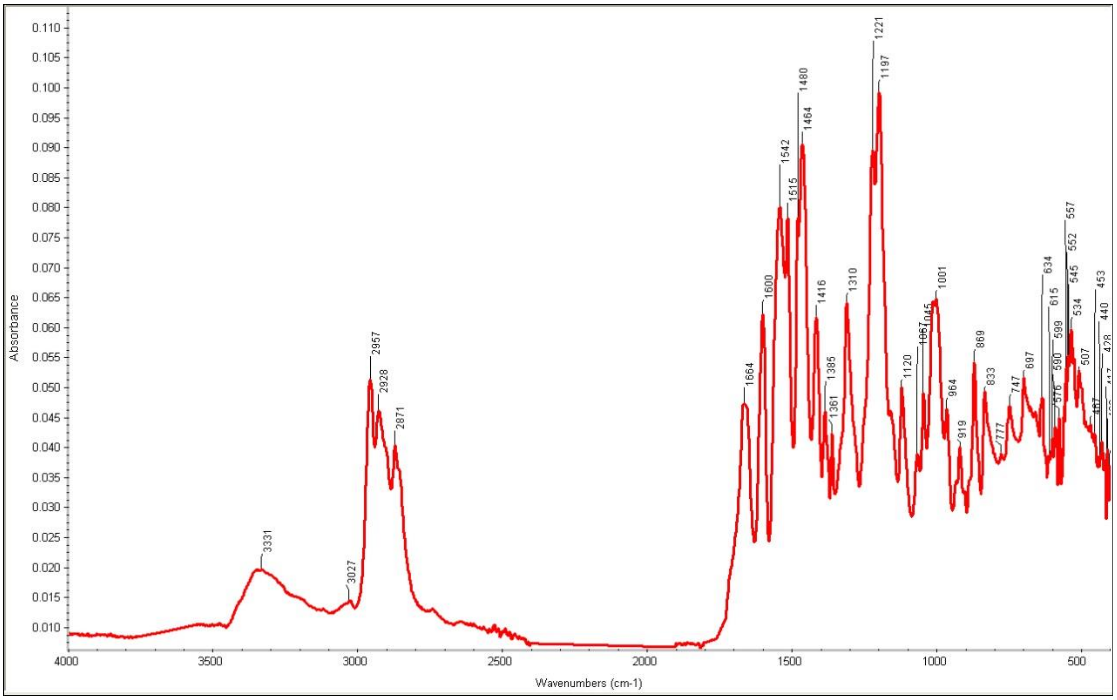

Figure 63. IR of compound **8b** (KBr).

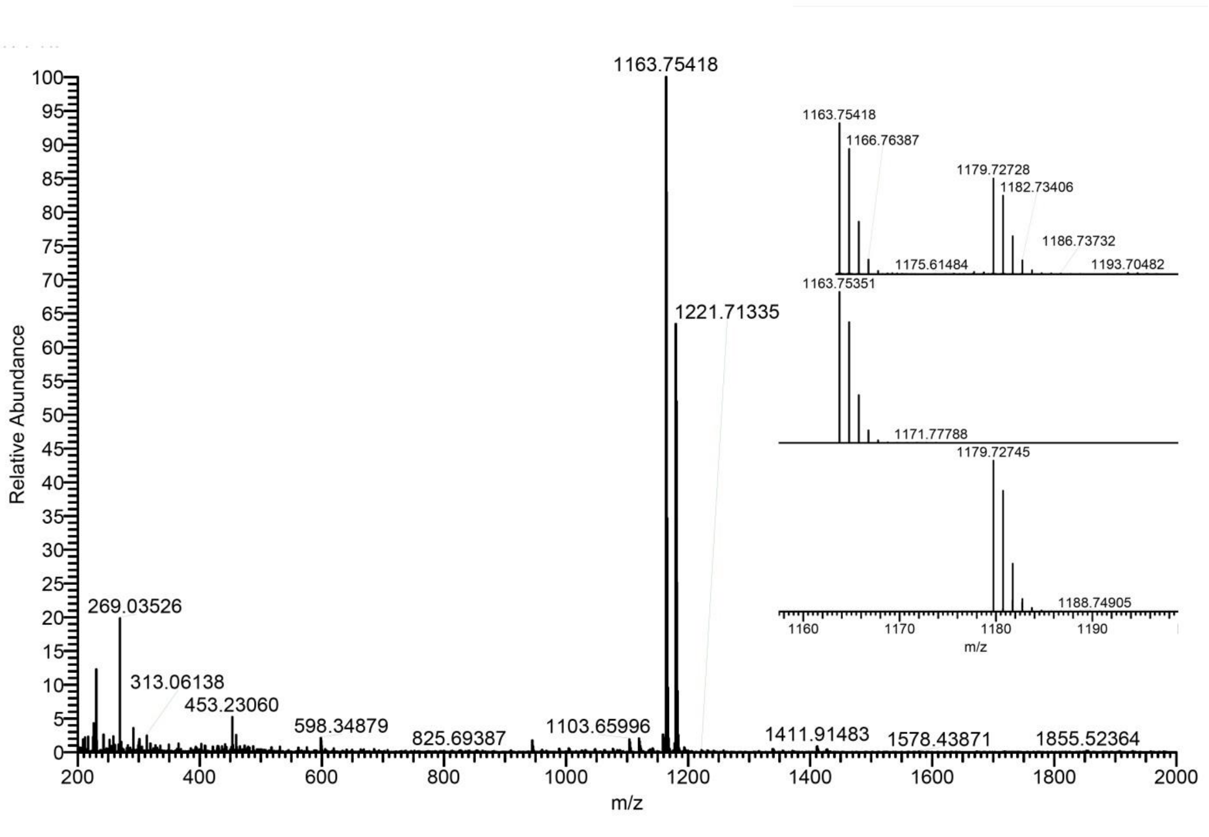

Figure 64. HRMS-ESI of compound **8b** (($C_{74}H_{100}N_4O_6$) m/z calcd: 1163.7535 $[M+Na]^+$, 1179.7275 $[M+K]^+$).

NMR titration data

Figure 65. ^{1}H NMR titration data for compound **7a** (DMSO-d_6, 400.1 MHz, 298 K).

NAcPhe c [mol/L] D-N-AcPhe	c 7a [mol/L] 3.4E-3 Δδ [Hz]	c [mol/L] L-N-AcPhe	c 7a [mol/L] 3.4E-3 Δδ [Hz]
0	0	0	0
0.000765	140.36	0.000764	137.32
0.002208	318.24	0.002204	357.64
0.003547	511.68	0.00354	511.32
0.004792	641.56	0.004782	651.68
0.005952	748.4	0.00594	759.56
0.009004	835.88	0.008986	877.88
0.011148	910.72	0.011125	925
0.014631	987.68	0.01298	999.8
0.017341	1008.24	0.016034	1053.12
0.039017	1190.28	0.038939	1200.2

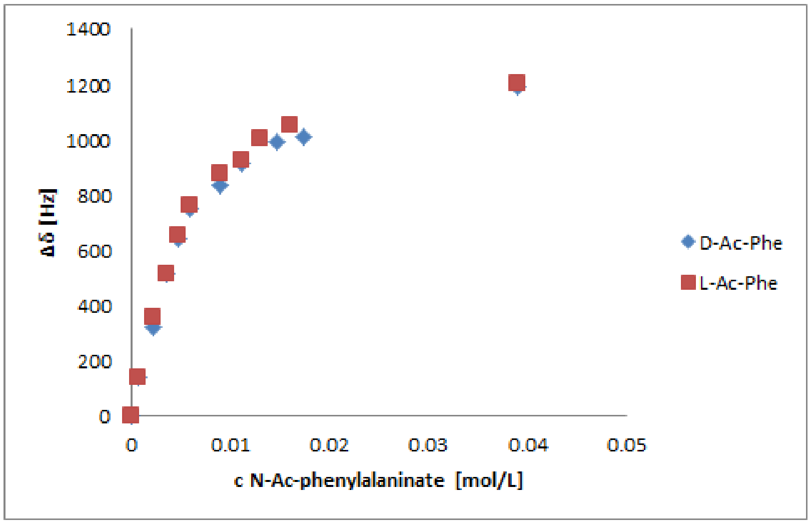

NAcLeu c [mol/L] D-N-AcLeu	c 7a [mol/L] 3.6E-3 Δδ [Hz]	c [mol/L] L-N-Leu	c 7a [mol/L] 3.6E-3 Δδ [Hz]
0	0	0	0
0.000866	52.72	0.000865	52.72
0.002499	163.84	0.002497	193.84
0.004014	272.12	0.00401	322.12
0.005422	362.92	0.005417	382.92
0.007359	463.08	0.007351	493.08
0.010189	564.96	0.010179	584.96
0.012615	626.96	0.012602	636.96
0.016557	691.52	0.01654	699.52
0.019623	743.16	0.019603	743.16
0.044151	885.6	0.044107	865.6

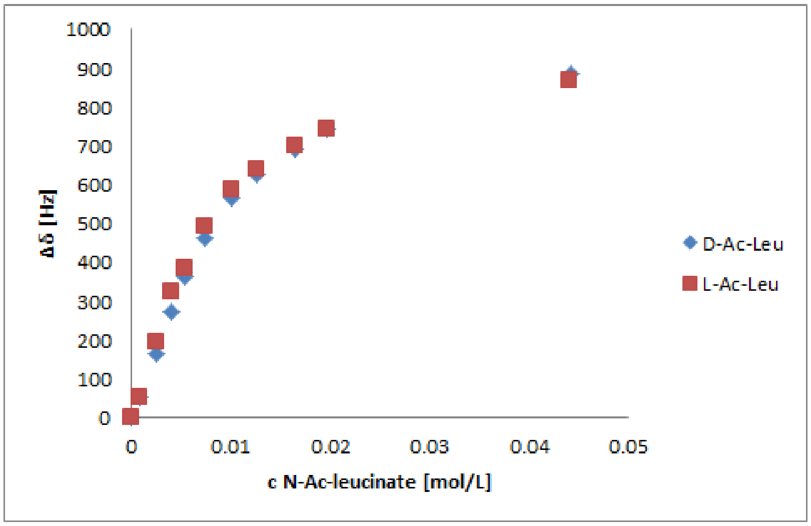

Phe c [mol/L] D-Phe	c 7a [mol/L] 3.4E-3 Δδ [Hz]	c [mol/L] L-Phe	c 7a [mol/L] 3.2E-3 Δδ [Hz]
0	0	0	0
0.000656	137.32	0.000673	128
0.001287	381.24	0.001321	381.24
0.001895	581.24	0.001944	613.24
0.003043	741.24	0.003122	785.24
0.004111	901.24	0.004217	877.24
0.005579	1065.24	0.005724	989.24
0.007725	1165.24	0.007925	1117.24
0.009564	1277.24	0.009812	1185.24
0.012552	1337.24	0.012878	1261.24
0.033473	1465.24	0.015263	1285.24
		0.034341	1424.24

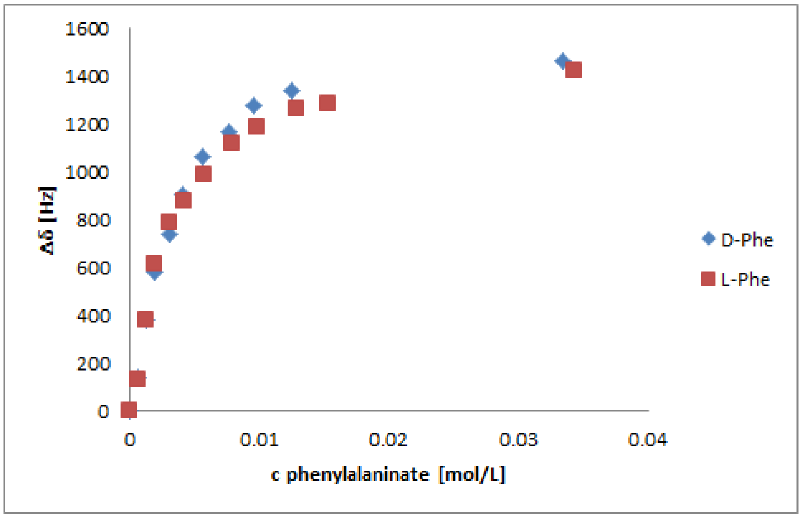

Leu c [mol/L] D-Leu	c 7a [mol/L] 3.2E-3 Δδ [Hz]	c [mol/L] L-Leu	c 7a [mol/L] 3.6E-3 Δδ [Hz]
0	0	0	0
0.001479	377.32	0.001291	229.6
0.00427	821.32	0.003726	741.6
0.006858	1041.32	0.005983	969.6
0.009265	1181.32	0.008083	1133.6
0.012574	1273.32	0.01097	1261.6
0.01741	1349.32	0.015189	1345.6
0.021555	1377.32	0.018805	1397.6
0.028291	1385.32	0.024682	1425.6
0.03353	1397.32	0.029252	1445.6
0.075443	1493.32	0.065818	1545.6

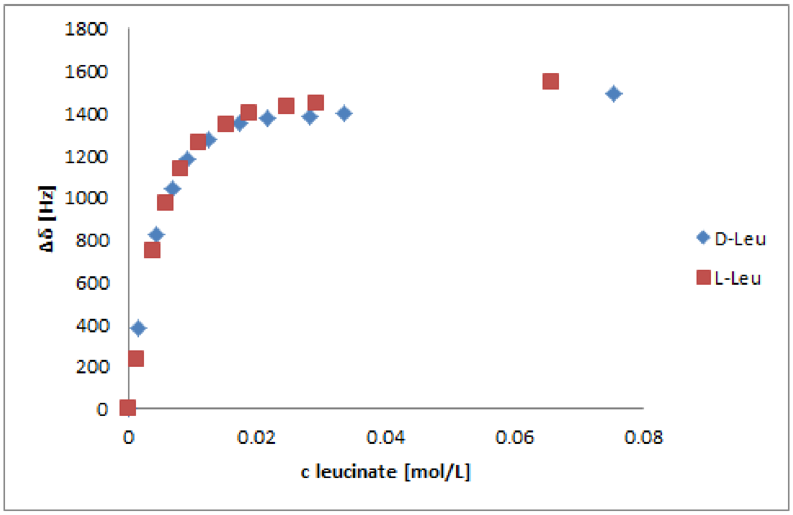

mandelate c [mol/L] R-mand	c 7a [mol/L] 3.2E-3 Δδ [Hz]	c [mol/L] S-mand	c 7a [mol/L] 3.2E-3 Δδ [Hz]
0	0	0	0
0.000837	83.68	0.000849	92.68
0.002415	262.16	0.002452	272.16
0.003879	370.04	0.003938	377.04
0.005241	439.96	0.005319	459.96
0.007112	544.84	0.007219	554.84
0.009848	639.92	0.009995	635.92
0.012192	684.92	0.012375	682.92
0.016002	721	0.016242	751
0.018966	761.96	0.01925	765.96
0.042673	880	0.043313	866

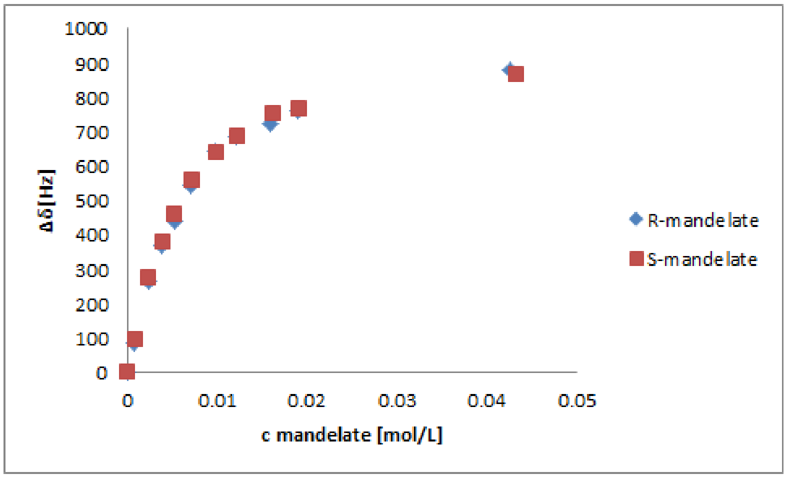

Figure 66. ^{1}H NMR titration data for compound **7b** (DMSO-d_6, 400.1 MHz, 298 K).

NAcPhe	c 7b [mol/L] 4.1E-3		c 7b [mol/L] 4.5E-3
c [mol/L] D-N-AcPhe	Δδ [Hz]	c [mol/L] L-N-AcPhe	Δδ [Hz]
0	0	0	0
0.00051	57.12	0.000533	62.44
0.001473	156.44	0.001539	164.12
0.002365	229.64	0.002472	266.64
0.003195	308.84	0.003339	327.96
0.004336	374.92	0.004532	414.28
0.006003	488.04	0.006275	507.12
0.007433	550.44	0.007769	566.64
0.009756	652.28	0.010197	648.92
0.011562	670.44	0.012085	697.44
0.026015	899.64	0.027192	902.32

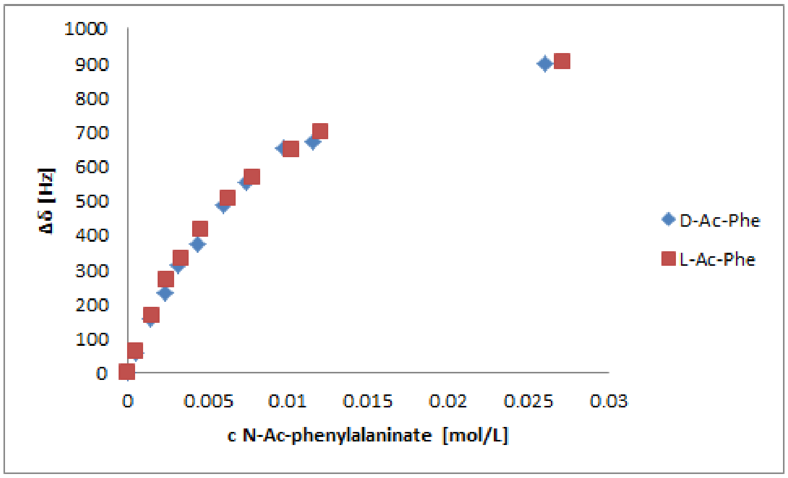

NAcLeu	c 7b [mol/L] 3.6E-3		c 7b [mol/L] 3.6E-3
c [mol/L] D-N-AcLeu	Δδ [Hz]	c [mol/L] L-N-AcLeu	Δδ [Hz]
0	0	0	0
0.001845	3.96	0.001932	8.12
0.005326	8.8	0.005577	24.08
0.008554	13.44	0.008957	34.28
0.011556	16.52	0.012099	43.44
0.014354	20.24	0.015029	52.6
0.018213	24.92	0.019069	60.4
0.023876	27.44	0.024998	71.32
0.031366	32.52	0.032841	76.36
0.038746	38.92	0.040568	82.08
0.094098	59.2	0.098522	114.72

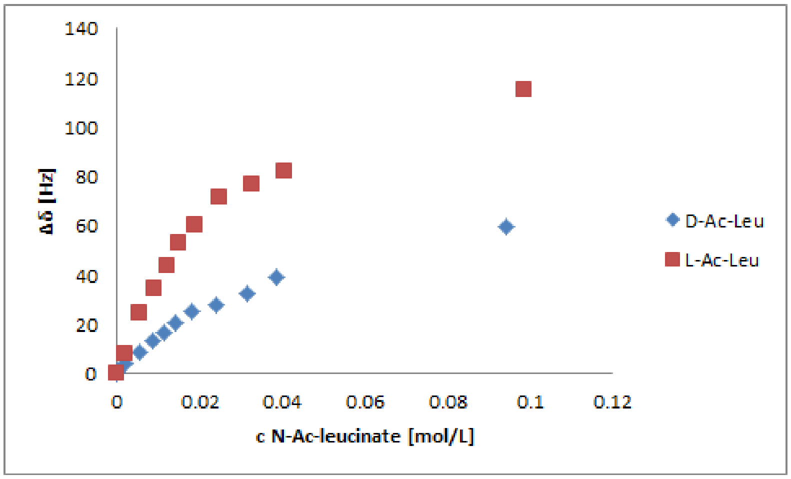

Phe	c 7b [mol/L] 3.5E-3		c 7b [mol/L] 3.5E-3
c [mol/L] D-Phe	Δδ [Hz]	c [mol/L] L-Phe	Δδ [Hz]
0	0	0	0
0.000705	57.48	0.000806	69.04
0.002034	174.52	0.002328	190.64
0.003267	264.12	0.003739	287.36
0.004413	338.12	0.005051	367.04
0.005989	428.48	0.006855	453.12
0.008293	520.8	0.009491	548.8
0.010267	604.64	0.011751	612.8
0.013476	684.8	0.015423	721.28
0.015971	755.68	0.018279	775.88
0.035935	1004.84	0.041129	1001.88

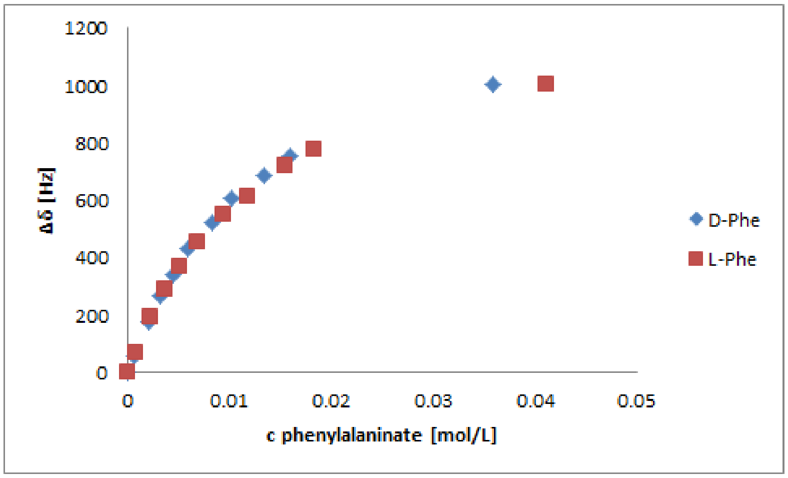

Leu	c 7b [mol/L] 3.3E-3		c 7b [mol/L] 3.3E-3
c [mol/L] D-Leu	Δδ [Hz]	c [mol/L] L-Leu	Δδ [Hz]
0	0	0	0
0.001678	118.96	0.001583	134.8
0.004844	372.64	0.004568	330.28
0.00778	500.8	0.007337	510.28
0.01051	577.84	0.009912	596
0.014264	685.4	0.013451	662.28
0.01975	784.72	0.018625	779.56
0.024452	850.16	0.02306	848.88
0.032094	921.92	0.030266	886.28
0.038037	950.24	0.035871	926.48
0.085584	1141.32	0.080709	1076.24

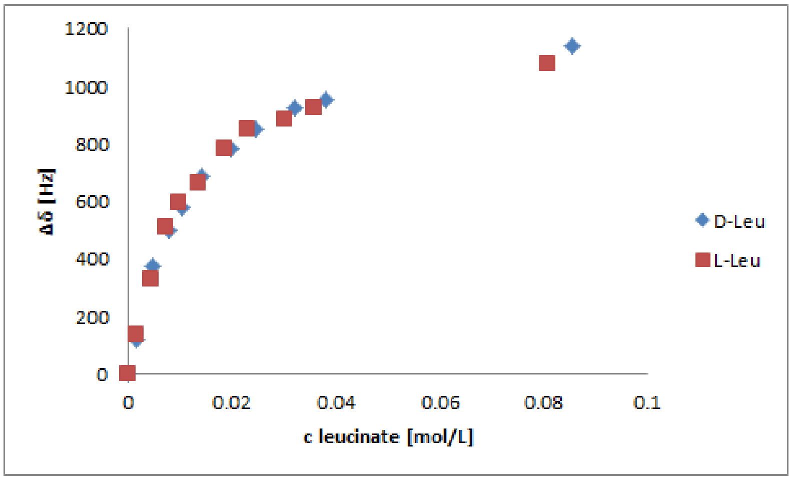

mandelate	c7b [mol/L] 2.7E-3		c 7b [mol/L] 2.7E-3
c [mol/L] R-mand	Δδ [Hz]	c [mol/L] S-mand	Δδ [Hz]
0	0	0	0
0.000765	59	0.000688	66.52
0.002208	155.28	0.001985	151.16
0.003547	225.84	0.003188	212.84
0.004792	290.48	0.004306	268.92
0.005952	338.92	0.005349	327.2
0.007552	388.28	0.006786	372.92
0.0099	440.16	0.008897	455.36
0.013006	495.2	0.011688	501.68
0.016066	523.76	0.014438	545.28
0.039017	655.92	0.035063	698.8

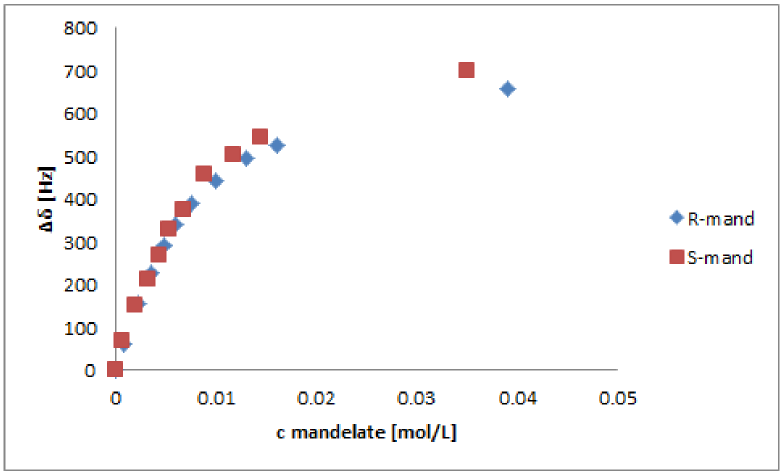

Figure 67. ^{1}H NMR titration data for compound **7c** (DMSO-d_6, 400.1 MHz, 298 K).

NAcPhe	c 7c [mol/L] 4.2E-3		c 7c [mol/L] 4.2E-3
c [mol/L] D-N-AcPhe	Δδ [Hz]	c [mol/L] L-N-AcPhe	Δδ [Hz]
0	0	0	0
0.0005332	1.572	0.0005412	1.372
0.0015392	2.492	0.0015622	3.852
0.002472	3.492	0.0025091	5.772
0.0033393	4.692	0.0033894	7.652
0.004532	6.012	0.0045999	10.092
0.006275	7.652	0.0063692	13.252
0.0077691	9.572	0.0078856	15.852
0.0101969	11.612	0.0103499	19.612
0.0120852	13.372	0.0122665	21.252
0.0167334	18.932	0.0169844	26.132
0.0271918	26.9	0.0275997	34.932
0.0543836	42.132	0.0551993	44.932

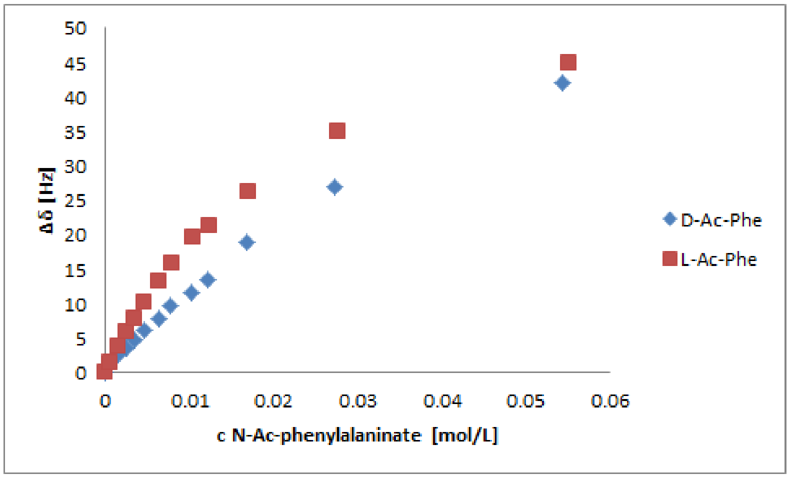

NAcLeu	c 7c [mol/L] 3.4E-3		c 7c [mol/L] 3.4E-3
c [mol/L] D-N-AcLeu	Δδ [Hz]	c [mol/L] L-N-AcLeu	Δδ [Hz]
0	0	0	0
0.0010758	1.0512	0.0010865	2.08
0.0031056	4.1464	0.0031366	6.28
0.0049877	6.1612	0.0050376	9.4
0.0067378	8.5264	0.0068052	12.56
0.0091442	10.9792	0.0092356	16.4
0.0126612	13.9576	0.0127878	20.72
0.0156757	16.206	0.0158325	23.68
0.0205744	19.3888	0.0207801	28.2
0.0243844	21.1408	0.0246283	30.56
0.03465	27.01	0.0349965	34.6
0.054865	35.478	0.0554137	39.84
0.104865	46.574	0.1059137	48.6

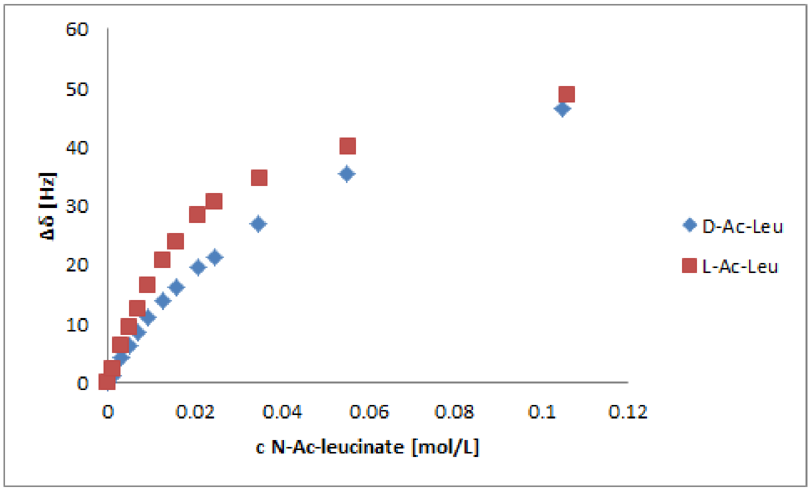

Phe	c 7c [mol/L] 6.5 E-3		c 7c [mol/L] 6.5E-3
c [mol/L] D-Phe	Δδ [Hz]	c [mol/L] L-Phe	Δδ [Hz]
0	0	0	0
0.0013627	11.8	0.0017135	52.36
0.0039338	51.8	0.0049466	132.88
0.0063179	90.16	0.0079445	191.92
0.0085347	118.4	0.010732	238.12
0.0115828	149.6	0.0145649	293.64
0.0160378	187.08	0.0201667	355.8
0.0198563	215.36	0.0249683	400.16
0.0260614	242.08	0.032771	439.8
0.0308875	267.64	0.0359267	477.92
0.069497	382	0.0873892	627.88

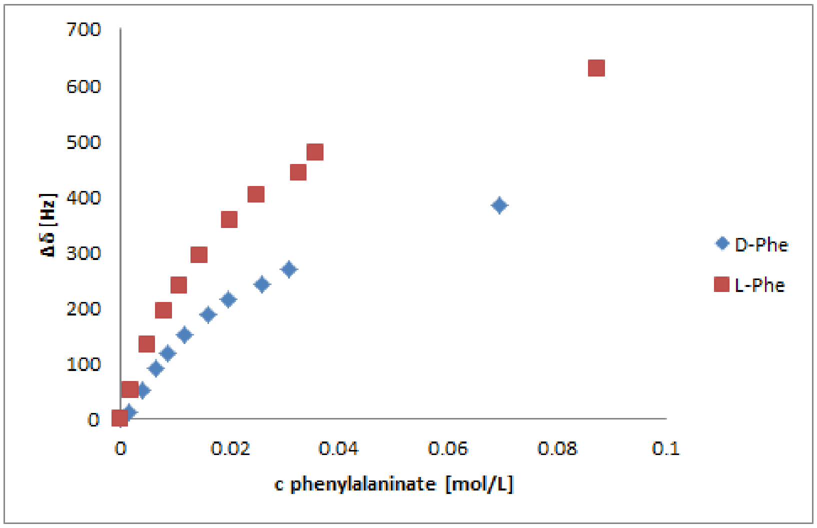

Leu	c 7c [mol/L] 5.1 E-3		c 7c [mol/L] 5.1E-3
c [mol/L] D-Leu	Δδ [Hz]	c [mol/L] L-Leu	Δδ [Hz]
0	0	0	0
0.0012376	31.88	0.0012624	29.8
0.0035727	87.8	0.0036441	90.88
0.005738	119.6	0.0058527	134.24
0.0077513	170.04	0.0079063	183.8
0.0105196	218.08	0.01073	222.92
0.0145656	271.96	0.0148569	283.8
0.0180336	287.4	0.0183943	302.04
0.0236691	352.32	0.0241425	359.8
0.0280522	367.92	0.0286133	371.8
0.0631175	520	0.0643799	500

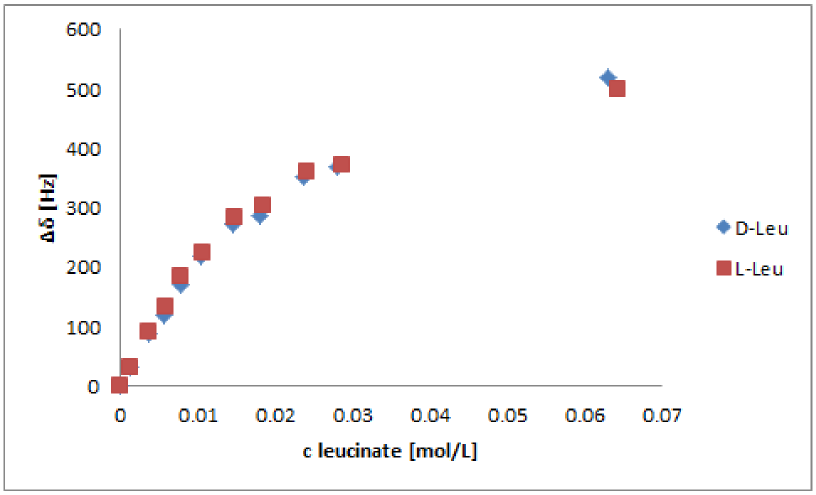

mandelate	c 7c [mol/L] 4.6 E-3		c 7c [mol/L] 4.6 E-3
c [mol/L] R-mand	Δδ [Hz]	c [mol/L] S-mand	Δδ [Hz]
0	0	0	0
0.0011387	1.32	0.0011444	1.64
0.0032873	4.04	0.0033038	3.72
0.0052796	6.08	0.005306	5.64
0.0071321	7.76	0.0071678	9.04
0.0096793	10.08	0.0097277	10.72
0.0134021	14.16	0.0134691	14.16
0.0165931	16.16	0.0166761	16.64
0.0217785	19.8	0.0218874	20.4
0.0258115	23.28	0.0259406	22.04
0.0580759	35.28	0.0583663	31.16

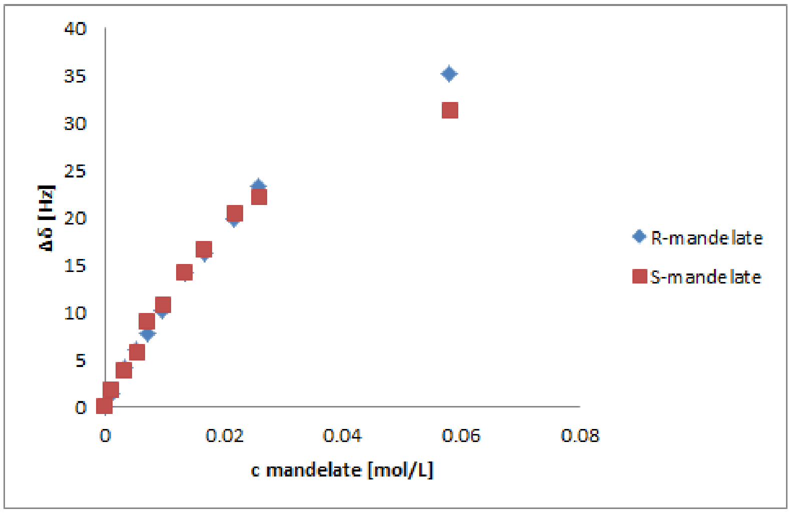

Figure 68. ^{1}H NMR titration data for compound **7d** (DMSO-d_6, 400.1 MHz, 298 K).

NAcPhe	c 7d [mol/L] 6.5E-3		c 7d [mol/L] 6.1E-3
c [mol/L] D-N-AcPhe	Δδ [Hz]	c [mol/L] L-N-AcPhe	Δδ [Hz]
0	0	0	0
0.001363	11.8	0.001276	16.68
0.003934	51.8	0.003682	47.4
0.006318	90.16	0.005914	67.28
0.008535	118.4	0.007989	95.16
0.011583	149.6	0.010843	127.72
0.016038	187.08	0.015013	163.6
0.019856	215.36	0.018588	198.24
0.026061	242.08	0.024396	235.6
0.030888	267.64	0.028914	255.6
0.069497	380	0.065057	418.24

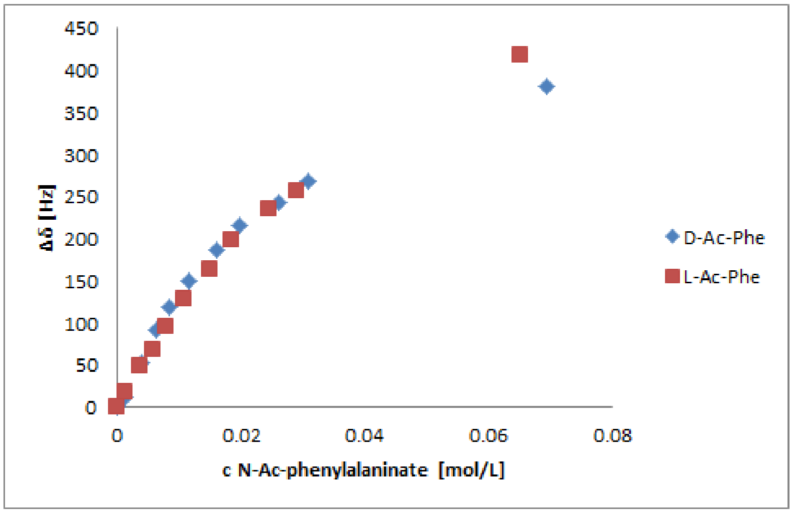

NAcLeu	c 7d [mol/L] 5.1E-3		c 7d [mol/L] 5.1E-3
c [mol/L] D-N-AcLeu	Δδ [Hz]	c [mol/L] L-N-AcLeu	Δδ [Hz]
0	0	0	0
0.001366	51.56	0.001305	26.8
0.003944	96.72	0.003844	64.72
0.006334	133.36	0.006234	112.52
0.008556	168.08	0.008656	144.52
0.011611	198.16	0.011811	180.52
0.016077	251.64	0.016774	228.52
0.019905	279.96	0.019905	260.52
0.023223	309.8	0.026126	310.64
0.028687	341.2	0.030964	341.64
0.033001	362.8	0.069669	539.76
0.069669	551.76		

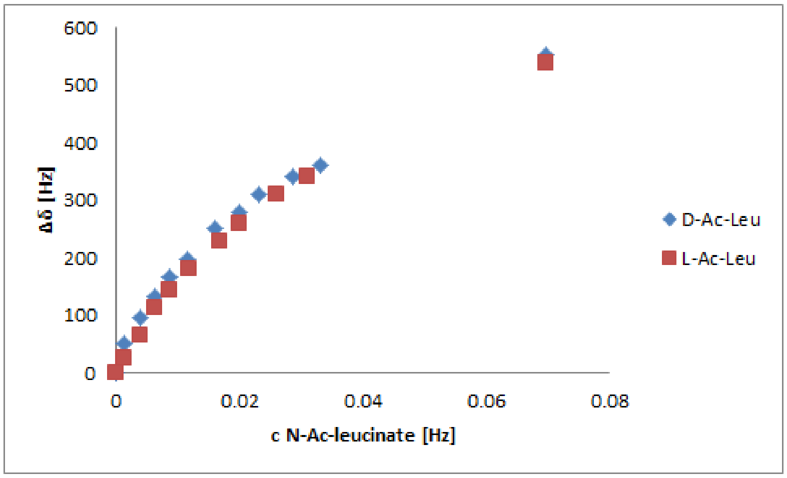

Phe	c 7d [mol/L] 6.5E-3		c 7d [mol/L] 6.5E-3
c [mol/L] D-Phe	Δδ [Hz]	c [mol/L] L-Phe	Δδ [Hz]
0	0	0	0
0.001714	32.16	0.001696	52.36
0.004947	81.36	0.004897	132.88
0.007944	136.96	0.007865	191.92
0.010732	173.96	0.010625	238.12
0.014565	200.6	0.014419	293.64
0.020167	264.32	0.019965	355.8
0.024968	298.84	0.024719	400.16
0.032771	345.6	0.032443	439.8
0.035927	371.04	0.035567	477.92
0.087389	569.76	0.086515	727.88

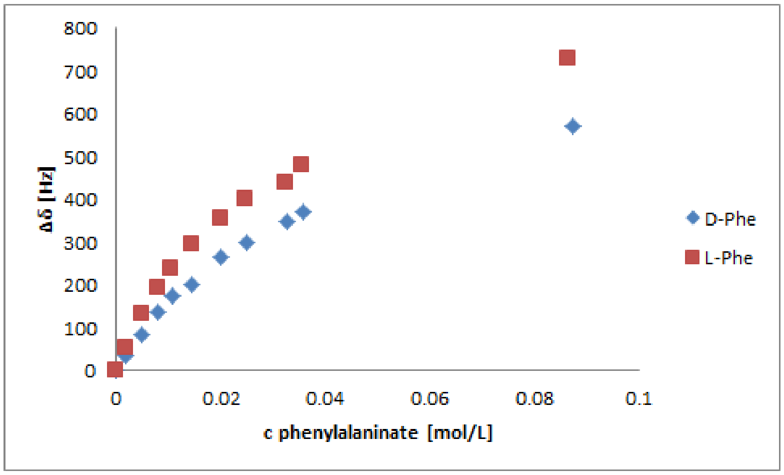

Leu	c 7d [mol/L] 5.1E-3		c 7d [mol/L] 5.1E-3
c [mol/L] D-Leu	Δδ [Hz]	c [mol/L] L-Leu	Δδ [Hz]
0	0	0	0
0.001238	31.88	0.001236	29.8
0.003573	87.8	0.003569	90.88
0.005738	119.6	0.005732	134.24
0.007751	170.04	0.007744	183.8
0.01052	218.08	0.010509	222.92
0.014566	271.96	0.014551	283.8
0.018034	287.4	0.018016	302.04
0.023669	352.32	0.023645	359.8
0.028052	367.92	0.028024	371.8
0.063118	617.48	0.063054	620.76

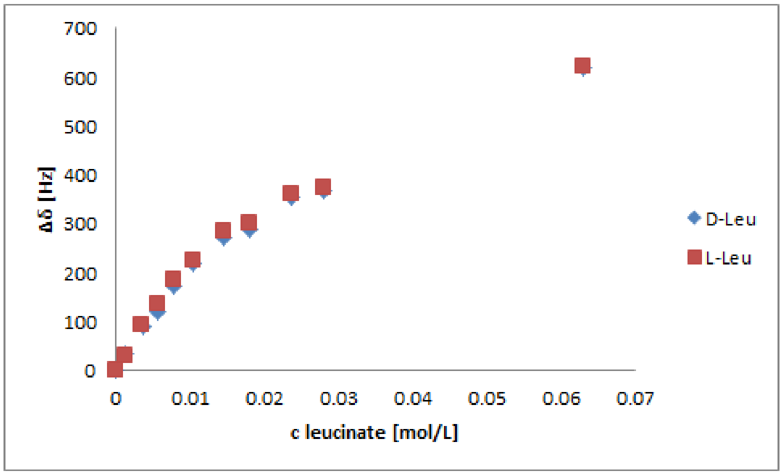

mandelate	c 7d [mol/L] 5.1E-3		c 7d [mol/L] 5.4E-3
c [mol/L] D-mand	Δδ [Hz]	c [mol/L] L-mand	Δδ [Hz]
0	0	0	0
0.001538	9.16	0.001553	10.28
0.004439	26.48	0.004483	25.76
0.007129	40.76	0.0072	40.4
0.00963	49.44	0.009726	51.64
0.013069	63.48	0.0132	69.2
0.018096	83.08	0.018277	89.32
0.022405	99.72	0.022629	103.84
0.029406	119.96	0.0297	123.96
0.032238	130.16	0.03256	139.96
0.078416	251.96	0.079201	252.92

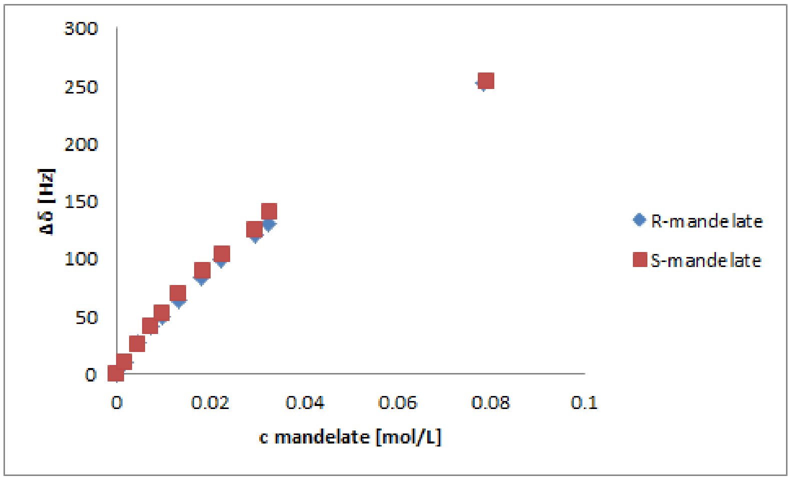

Figure 69. ^{1}H NMR titration data for compound **8a** (DMSO-d_6, 400.1 MHz, 298 K).

NAcPhe c [mol/L] D-N-AcPhe	c 8a [mol/L] 5.9E-3 Δδ [Hz]	c [mol/L] L-N-AcPhe	c 8a [mol/L] 5.9E-3 Δδ [Hz]
0	0	0	0
0.001314	101	0.001393	150.52
0.003793	354.76	0.004023	378.36
0.006092	515.04	0.00646	589.88
0.00823	658.28	0.008727	710.16
0.01117	785.84	0.011844	823.36
0.015466	915.04	0.0164	932.08
0.019148	979.04	0.020304	994.24
0.025131	1039.04	0.026649	1046.88
0.029785	1075.04	0.031584	1087.52
0.067017	1145.04	0.071065	1184.12

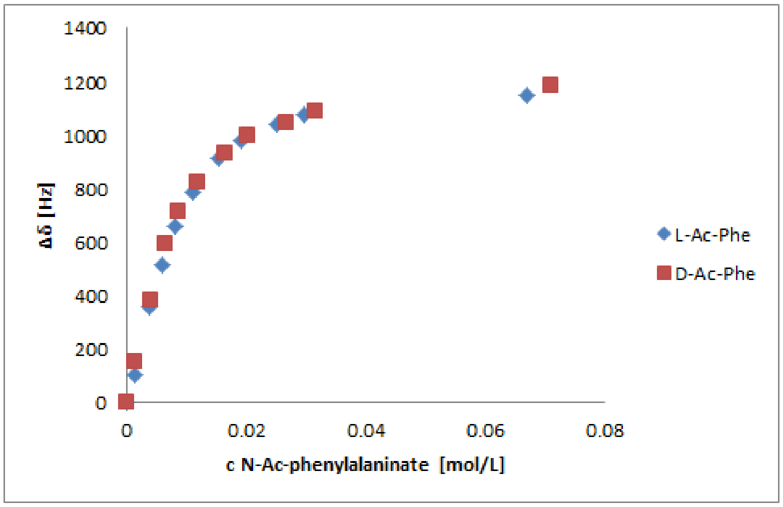

NAcLeu c [mol/L] D-N-AcLeu	c 8a [mol/L] 5.9E-3 Δδ [Hz]	c [mol/L] L-N-AcLeu	c 8a [mol/L] 5.9E-3 Δδ [Hz]
0	0.00	0	0
0.001445	139.86	0.001314	101
0.004173	357.74	0.003793	354.76
0.006702	513.10	0.006092	515.04
0.009053	625.52	0.00823	658.28
0.012287	742.51	0.01117	785.84
0.017012	860.71	0.015466	915.04
0.021063	930.56	0.019148	979.04
0.027645	1008.05	0.025131	1039.04
0.032764	1049.62	0.029785	1075.04
0.060316	1159.45	0.067017	1183.04

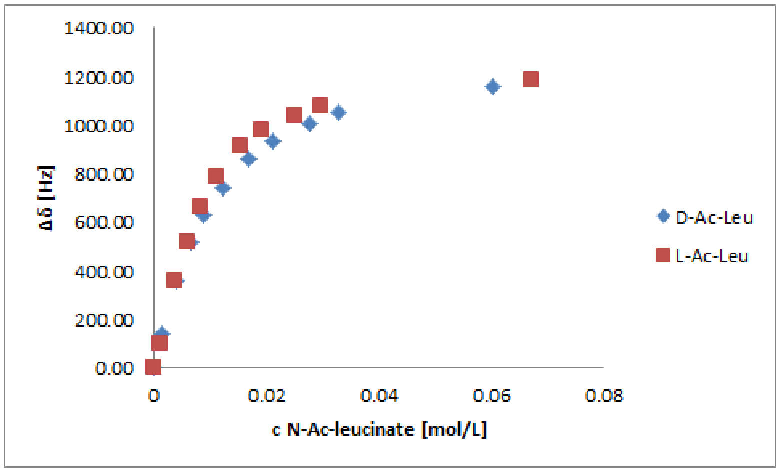

Phe	c 8a [mol/L] 5.9E-3		c 8a [mol/L] 5.9E-3
c [mol/L] D-Phe	Δδ [Hz]	c [mol/L] L-Phe	Δδ [Hz]
0	0.0	0	0
0.001091	190.5	0.001102	181.92
0.00315	504.9	0.003182	486.4
0.005059	732.0	0.005111	696.48
0.006835	886.1	0.006904	858.08
0.009276	1026.6	0.009369	1003.08
0.012843	1142.1	0.012973	1153.68
0.015901	1198.0	0.016062	1217.4
0.02087	1250.9	0.021081	1287
0.055654	1343.6	0.056216	1335

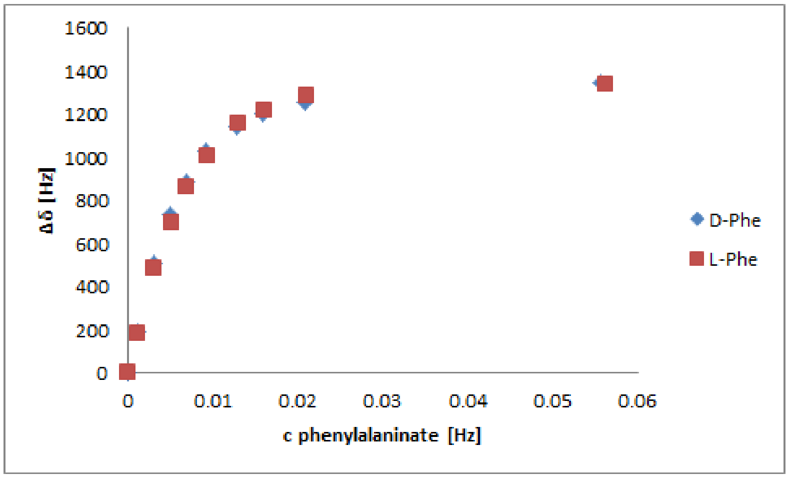

Leu	c 8a [mol/L] 4.5E-3		c 8a [mol/L] 4.5E-3
c [mol/L] D-Leu	Δδ [Hz]	c [mol/L] L-Leu	Δδ [Hz]
0	0	0	0
0.001102	153.8	0.001108	181.8
0.003182	401.8	0.003282	469.8
0.005111	617.8	0.005058	653.8
0.00789	841.8	0.006904	789.8
0.01218	993.8	0.009369	901.8
0.015568	1037.8	0.012973	1017.8
0.022487	1101.8	0.016062	1069.8
0.026703	1117.8	0.021081	1113.8
0.056216	1237.8	0.024985	1145.8
		0.056216	1241.8

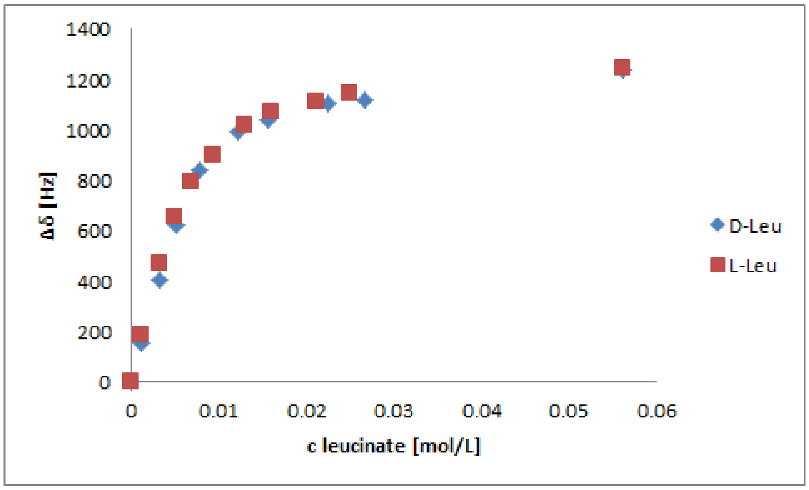

mandelate	c 8a [mol/L] 3.6E-3		c 8a [mol/L] 3.6E-3
c [mol/L] R-mand	Δδ [Hz]	c [mol/L] S-mand	Δδ [Hz]
0	0	0	0
0.000608	76.688	0.000608	79.688
0.001192	206.568	0.001192	186.568
0.001755	301.288	0.001754	241.288
0.002818	365.328	0.002818	355.328
0.003807	467.648	0.003806	457.648
0.005166	550.168	0.005166	540.168
0.007153	623.808	0.007152	633.808
0.008856	704.408	0.008855	714.408
0.011624	768.448	0.011623	776.448
0.030997	1002.928	0.030994	989.928

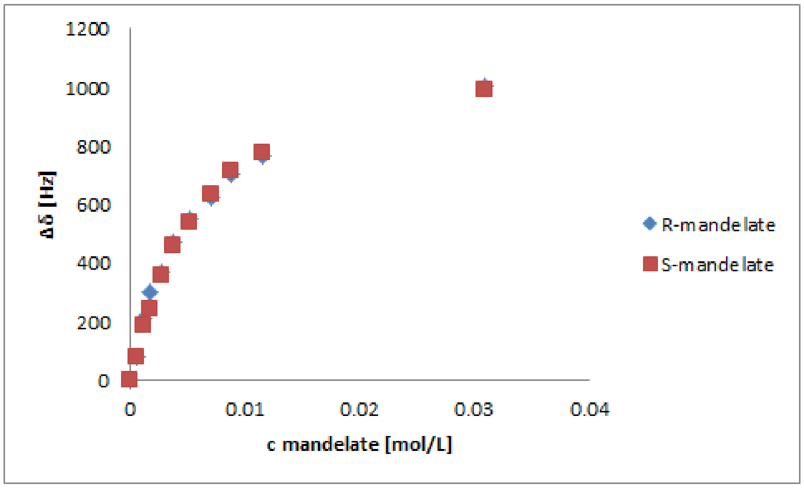

Figure 70. ^{1}H NMR titration data for compound **8b** (DMSO-d_6, 400.1 MHz, 298 K).

NAcPhe	c 8b [mol/L] 3.8E-3		c 8b [mol/L] 3.8E-3
c [mol/L] D-N-AcPhe	Δδ [Hz]	c [mol/L] L-N-AcPhe	Δδ [Hz]
0	0	0	0
0.000456	54.92	0.000479	55.4
0.001317	137.16	0.001384	145.92
0.002116	214.28	0.002223	236.44
0.002858	284.64	0.003002	290.28
0.003879	357.92	0.004075	366.16
0.005371	443.28	0.005642	448.6
0.006649	494.92	0.006985	501.64
0.008727	574.64	0.009168	574.92
0.010344	624.64	0.010866	617.96
0.023273	789.92	0.024448	791.44

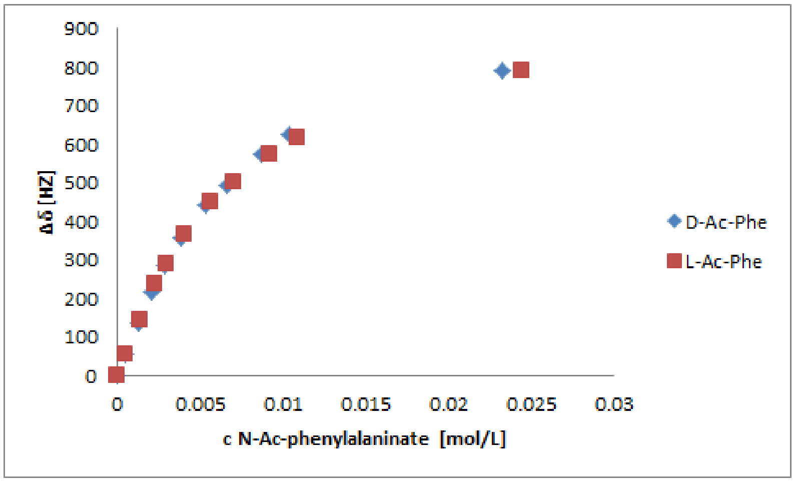

NAcLeu	c 8b [mol/L] 4E-3		c 8b [mol/L] 4E-3
c [mol/L] D-N-AcLeu	Δδ [Hz]	c [mol/L] L-N-AcLeu	Δδ [Hz]
0	0	0	0
0.001759	3.96	0.001672	10.04
0.005077	8.8	0.004828	23.4
0.008153	13.44	0.007754	41.96
0.011014	16.52	0.010474	51.6
0.014948	20.24	0.014215	64.92
0.020697	24.92	0.019682	72.92
0.025625	28.44	0.024368	85.96
0.033632	34.52	0.031983	93.32
0.039861	39.92	0.037906	97.08
0.089687	60.24	0.085289	141.92

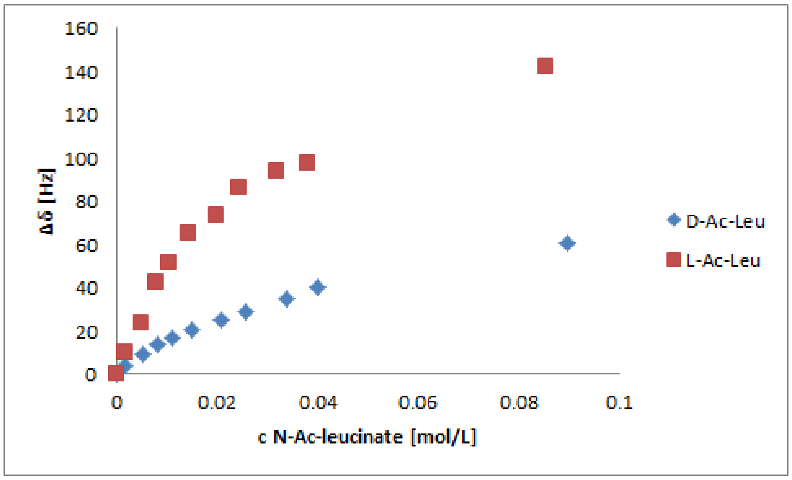

Phe	c 8b [mol/L] 3.5E-3		c 8b [mol/L] 3.5E-3
c [mol/L] D-Phe	Δδ [Hz]	c [mol/L] L-Phe	Δδ [Hz]
0	0	0	0
0.000806	69.08	0.000806	64.68
0.002328	208.76	0.002328	167.52
0.003739	311.96	0.003739	303.52
0.005051	379.48	0.005051	398.88
0.006855	494.8	0.006855	486.96
0.009491	591.52	0.009491	588.28
0.011751	667	0.011751	651.8
0.015423	750.88	0.015423	737.72
0.018279	794.8	0.018279	776.32
0.041129	1030	0.041129	1035.32

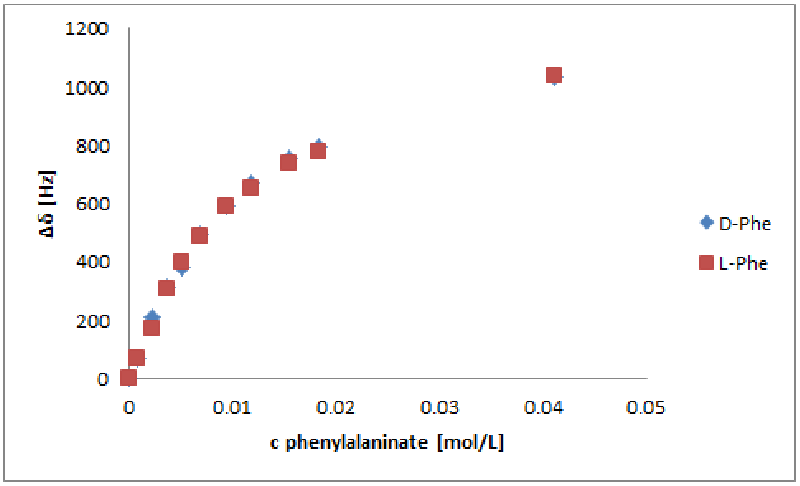

Leu	c 8b [mol/L] 3.9E-3		c 8b [mol/L] 3.9E-3
c [mol/L] D-Leu	Δδ [Hz]	c [mol/L] L-Leu	Δδ [Hz]
0	0	0	0
0.000773	48.92	0.000771	46.92
0.002231	129.12	0.002203	117.16
0.003583	208.96	0.003519	183.28
0.00484	256.84	0.004789	243.16
0.006569	324.44	0.006434	305.92
0.009095	397.16	0.008086	358.96
0.011261	443.92	0.010173	412.64
0.01478	508.2	0.013928	490.64
0.017517	546.16	0.016919	534.08
0.039413	745.8	0.038067	700

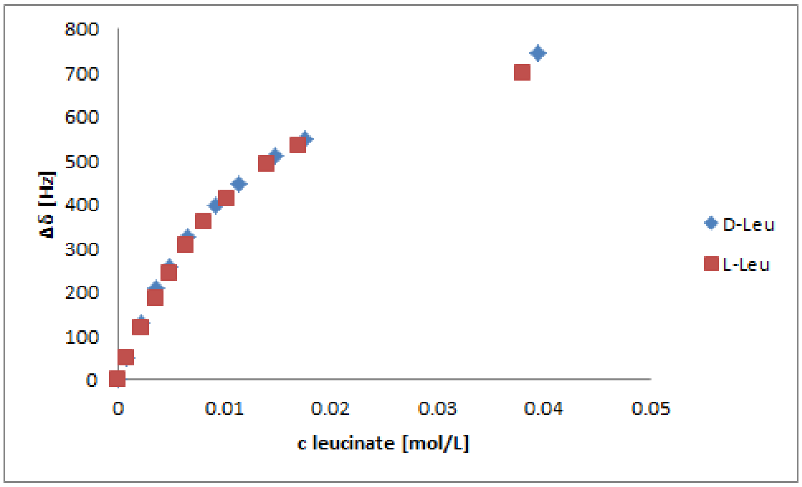

mandelate	c 8b [mol/L] 4.5E-3		c 8b [mol/L] 4.6E-3
c [mol/L] D-mand	Δδ [Hz]	c [mol/L] L-Leu	Δδ [Hz]
0	0	0	0
0.000626	42.36	0.00061	40.2
0.001806	110.88	0.001762	108.2
0.002901	161.8	0.002829	168.88
0.003919	193.8	0.003822	203.2
0.005318	253.24	0.005187	244.24
0.007364	307.44	0.007182	311.12
0.009117	361.64	0.008892	338.76
0.011966	399.44	0.011671	391.16
0.014182	429.88	0.013832	427
0.031909	574	0.031122	553

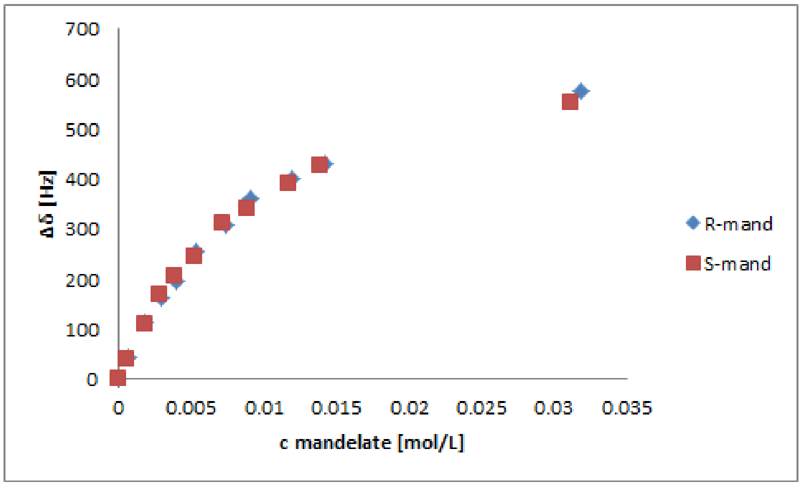

Supplement: File 1 — Experimental details and characterisation data (including X-ray data for 4a, 7a, and 7d, NMR, IR, and HRMS) as well as NMR titration data. [file Beilstein_J_Org_Chem-16-2999-s001.pdf]
